# Human Mental Workload: A Survey and a Novel Inclusive Definition

**DOI:** 10.3389/fpsyg.2022.883321

**Published:** 2022-06-02

**Authors:** Luca Longo, Christopher D. Wickens, Gabriella Hancock, P. A. Hancock

**Affiliations:** ^1^Artificial Intelligence and Cognitive Load Lab, The Applied Intelligence Research Centre, School of Computer Science, Technological University Dublin, Dublin, Ireland; ^2^Department of Psychology, Colorado State University, Fort Collins, CO, United States; ^3^Department of Psychology, California State University, Long Beach, CA, United States; ^4^Department of Psychology, Institute for Simulation and Training, University of Central Florida, Orlando, FL, United States

**Keywords:** survey, mental workload, definitions, theories, measures, models, novel framework, novel inclusive definition

## Abstract

Human mental workload is arguably the most invoked multidimensional construct in Human Factors and Ergonomics, getting momentum also in Neuroscience and Neuroergonomics. Uncertainties exist in its characterization, motivating the design and development of computational models, thus recently and actively receiving support from the discipline of Computer Science. However, its role in human performance prediction is assured. This work is aimed at providing a synthesis of the current state of the art in human mental workload assessment through considerations, definitions, measurement techniques as well as applications, Findings suggest that, despite an increasing number of associated research works, a single, reliable and generally applicable framework for mental workload research does not yet appear fully established. One reason for this gap is the existence of a wide swath of operational definitions, built upon different theoretical assumptions which are rarely examined collectively. A second reason is that the three main classes of measures, which are self-report, task performance, and physiological indices, have been used in isolation or in pairs, but more rarely in conjunction all together. Multiple definitions complement each another and we propose a novel inclusive definition of mental workload to support the next generation of empirical-based research. Similarly, by comprehensively employing physiological, task-performance, and self-report measures, more robust assessments of mental workload can be achieved.

## 1. Introduction

One of the primary goals of building interactive technologies, from a human factors perspective, has always been managing the mental workload (MWL) experienced by users. The primary motivation is the optimization of their performance, the enhancement of their engagement, alongside the minimization of their errors. All human activities include some amount of mental processing and thus, at least some degree of mental workload (Mitchell, 2000). Even the most rudimentary of physical or cognitive tasks involve some degree of mental processing, and consequently a resulting level of mental workload (Longo, 2011; Longo et al., 2012). Technological advances in the last two decades have shaped human-computer interaction in such a way that has reduced the human operator's physical load, while altering necessary cognitive processing in terms of its nature (passive vs. active) and quantity. The ultimate goal of these advances, from the commercialization of systems that support direct manipulation of graphical objects, to automated language translators and gesture recognition systems, has been to reduce and/or regulate the human operator's mental workload (Hancock and Chignell, 1988; Myers, 1998; Miller, 2001; Longo, 2015). The key focus has been to regulate the associated cognitive, visual, auditory, perceptual, psychomotor, and communication contributors to workload (Miller, 2001). However, research in the fields of learning and instructional design has indicated that the use of technology not only increases performance, but also often increases users' frustration (Hove and Corcoran, 2008). A critical evaluation of our current understanding of mental workload and the identification of key areas of progress remains extremely important because it can aid in the design of interactive technologies (Jex, 1988). Specifically, mental workload measurement is vital to the development of new technologies, information-based procedures and user interfaces that maximize human performance (Kantowitz, 1987; Hancock and Meshkati, 1988; Di Stasi et al., 2011; Meshkati and Hancock, 2011; Felton et al., 2012; Moray, 2013; Orru and Longo, 2020; Longo and Rajendran, 2021). Identifying such areas wherein users experience significant levels of mental workload, and trying to regulate it by system re-design, could also minimize human error, and in turn, increase user satisfaction, learning, and other operational advantages (Davenport and Beck, 2001). The significance of mental workload measurement is frequently expressed in the desirability of optimizing human-machine interactions (Ogden et al., 1979; De Waard and te Groningen, 1996; Young and Stanton, 1997; Hankins and Wilson, 1998; Neßelrath, 2013; Kajiwara, 2014; Paxion et al., 2014; Zhang et al., 2015). The key reason for measuring mental workload is to quantify the mental cost of performing tasks in order to predict operator and system responses. Other reasons to measure MWL include the acquisition of specific certifications, or compliance with certain industrial standards (Cain, 2007). For example, Web Content Accessibility Guidelines (WCAG) of the W3C group are today considered the industry standards on the universal design aspects of user-interfaces, and mental workload assessments can objectively facilitate such designs (Kelly et al., 2005).

Despite the manifest reasons for developing interactive technologies that support mental workload regulation across myriad safety-critical application domains including aviation, automobile, and maritime industries to mention just a few, there is, as yet, no universally accepted definition of mental workload (Wierwille, 1979; Wilson et al., 1994; Zeitlin, 1995; Veltman, 2002; Wilson, 2002; Averty et al., 2004; Young and Stanton, 2004; Colle and Reid, 2005; Di Nocera et al., 2007; Elmenhorst et al., 2009; Borghini et al., 2017). This lack of a comprehensive and universally accepted definition has not prevented the proliferation of experimental research about its effects and mitigation. Surveys and reviews have been performed on mental workload, but they are either domain-specific (Pearson et al., 2006), or they focus on one particular aspect of mental workload, such as a single measurement technique (Charles and Nixon, 2019).

This research work therefore presents a comprehensive survey of existing research across three foundational dimensions: i) the theoretical background of mental workload; ii) its current operational definitions and the introduction of a novel, inclusive operational definition synthesized from these disparate sources; iii) and MWL measurement methods and measures, for the recommendation of a new operational framework to guide future research. The precise research question and methods employed to select the body of published materials are first specified, and a novel research framework is then recommended for scholars.

## 2. Research Questions and Methods

In order to conduct a survey, the following objectives were set: i) framing the research question, ii) identifying relevant publications, iii) synthesizing the gathered knowledge, iv) interpreting the findings, and finally v) providing relevant recommendations. The main research question being *what is the state of the art in the study of mental workload and how can this be synthesized into a framework to guide future research?* This larger inquiry is divided into a set of more granular research questions:

- *Theoretical Background* - What is the theoretical background for mental workload? What are the theories that have been proposed and employed to investigate, measure, assess, validate and explain it?
- *Defining Mental Workload* - What are the operational definitions of mental workload coined by various researchers and across different time periods? How can these definitions be aggregated into a more inclusive definition?
- *Measuring Mental Workload* - What are the state of the art measures of mental workload and the relevant methods for assessing it? How can these measures be aggregated into a framework for guiding future research?

To begin, Google Scholar was used as the primary source for identifying and gathering published material. The overreaching search terms used were ‘mental workload' and ‘cognitive workload'. Google Scholar, at the time of querying, displayed 342, 000 results for the term “mental workload” and 311, 000 results for ‘cognitive workload'. The first 100 pages of results were considered, with each page containing 10 results. Thus, 1,000 entries for each keyword were considered. These entries were screened based on title and abstract, and a preliminary set of scientific articles was created. Works that only mentioned mental workload, but did not contribute either to its measurement, definition, or evaluation, nor applied existing assessment methods in specific application fields, were excluded. From remaining articles, bibliographic references were analyzed, and recursively identified work were considered for review. Many of these references had previously been identified via Google Scholar, but a number of additional sources were not. More than 500 relevant articles were consequently added to the set. Though many other manuscripts no doubt exist in the literature, and a systematic review is not feasible, an executive decision was taken that the selected materials were sufficiently representative to conduct a meaningful survey, and they could effectively exemplify the entire population of manuscripts, as well as a robust characterization of the state of the art concerning mental workload research. The selected manuscripts were carefully evaluated, and the following attributes were extracted in order to facilitate classification, synthesis and the production of descriptive statistics. These attributes were:

- year of publication and type (journal/conference/technical report)
- category of research (by type, form, objective, reasoning)
- domain of application, underpinning theory
- proposed definition of mental workload (if existing)
- evaluation of the reliability, validity, sensitivity or diagnosticity of the underlying model
- types of measures employed and details
- experimental sample size (if empirical research), and number of citations, to provide a portraiture of the breath of research on mental workload.

### 2.1. Descriptive Statistics

**Figure 1:**
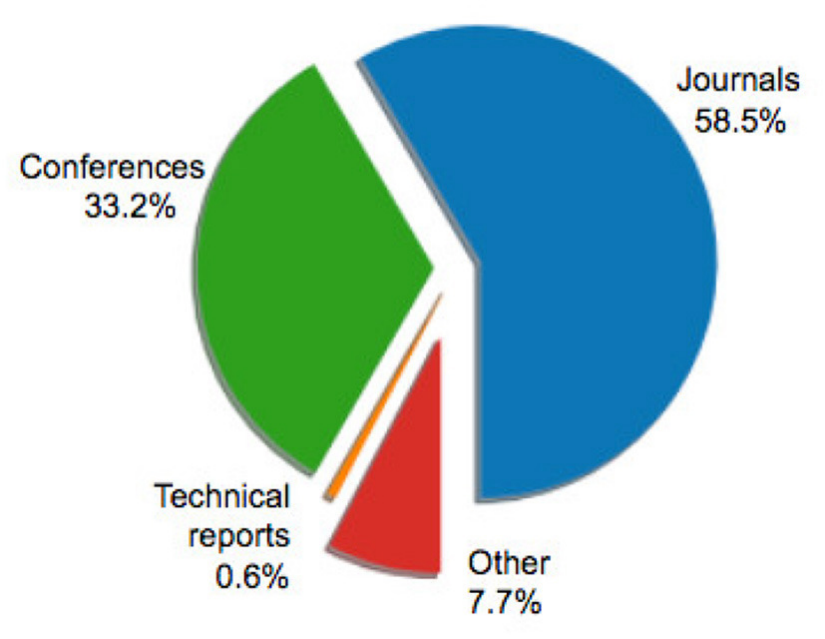
Distribution of sample articles by type.

**Figure 2:**
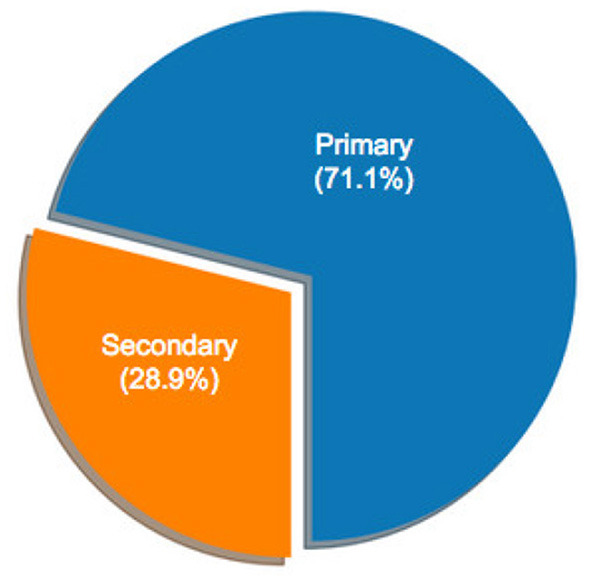
Percentage of sample articles by research type.

**Figure 3:**
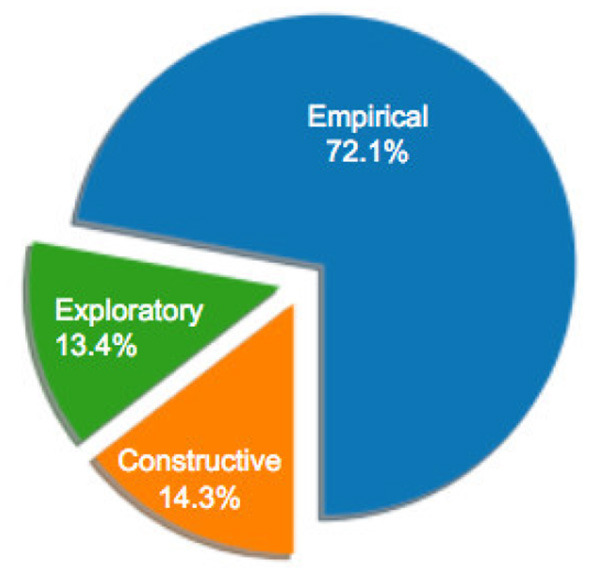
Percentage of sample articles by research form.

**Figure 4:**
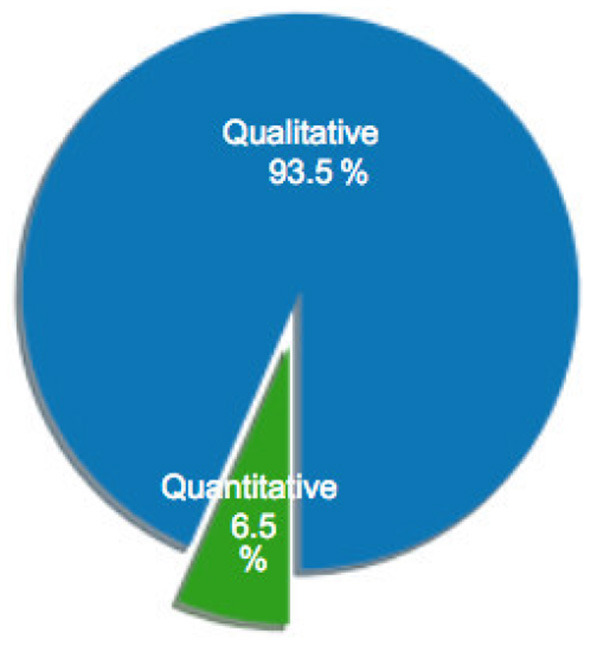
Percentage of sample articles by research objective.

**Figure 5:**
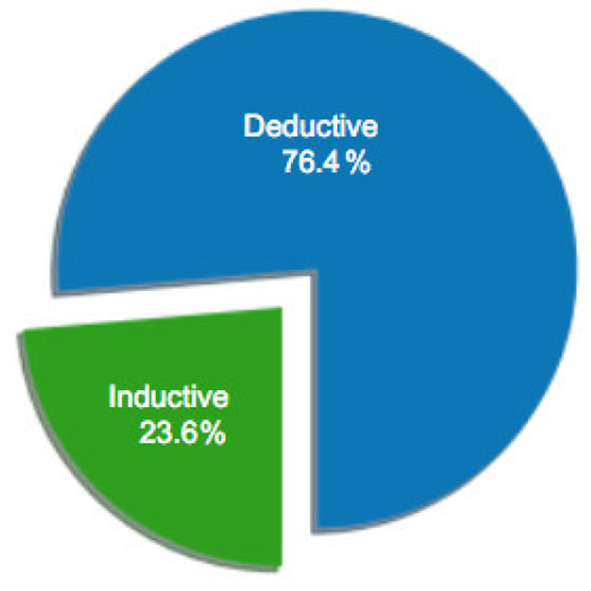
Percentage of sample articles by research reasoning.

**Figure 6:**
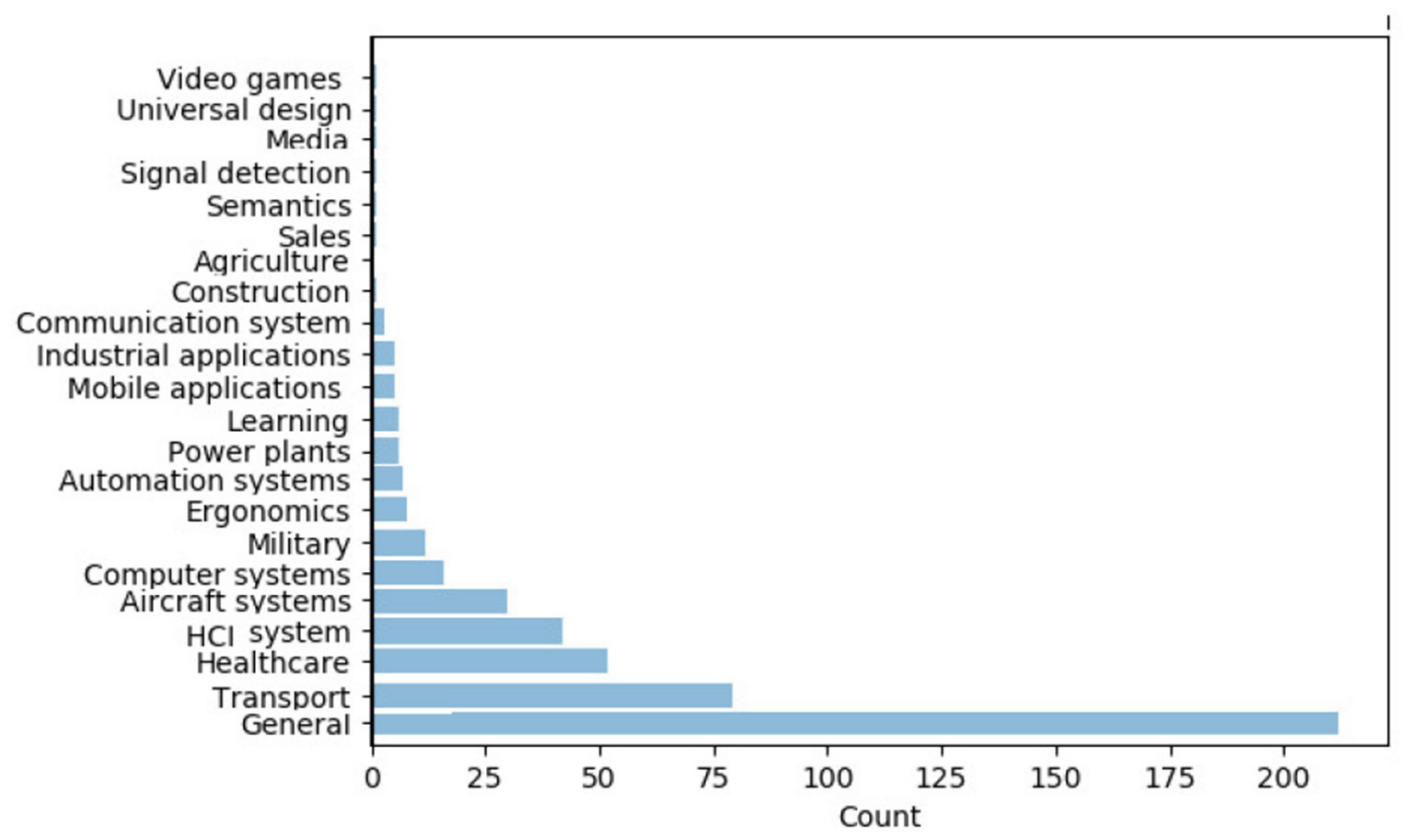
Distribution of the articles by domain of application.

**Figure 7:**
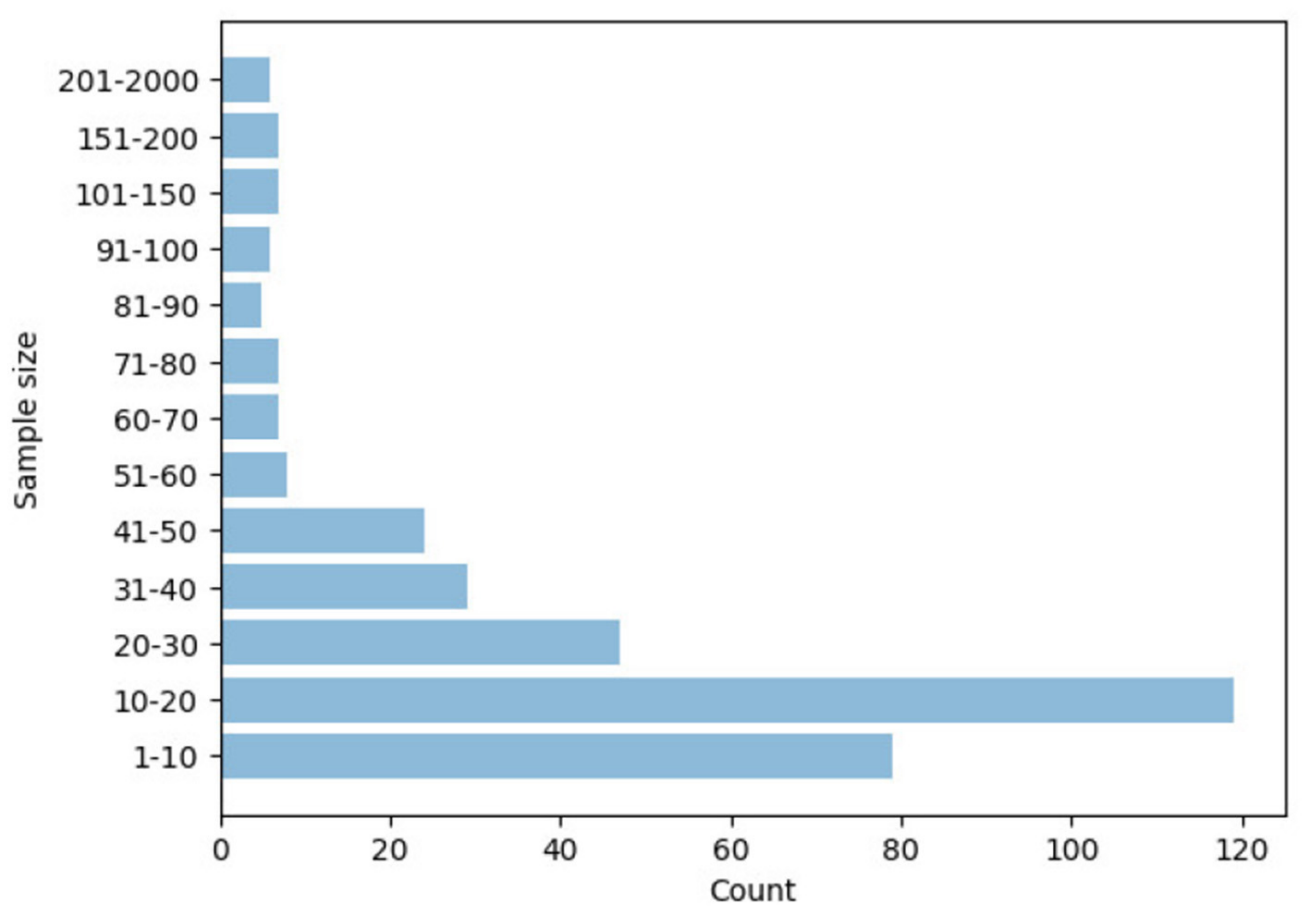
Distribution of the articles by sample size of participants.

Figure 1 illustrates the distribution of selected articles as grouped by publication venue. Figures 2–5 furthermore depicts the breakdown of these articles grouped by a) type, b) form of research, c) its objective and d) reasoning. Similarly, Figures 6, 7 categorizes the articles by a) domain of application and, b) the sample size of the included empirical studies. Exploratory research has a primary objective of structuring what is known and identifying new problems related to mental workload. Constructive research focuses more on developing novel solutions to identified problems. Empirical research focuses on testing the feasibility of a model of mental workload using experimental evidence (Hancock and Caird, 1993; Longo, 2014). Qualitative research is often more flexible, unstructured, and subjective, intended to explore phenomena related to the construct of mental workload. Quantitative research is generally more stable, structured, and objective, being aimed at addressing specific hypotheses about mental workload (Graziano and Raulin, 1993). Research articles based on deductive reasoning follow a top-down approach wherein hypotheses on mental workload, are often based on existing theories, and are supported or refuted through experimental observations. Research articles based on inductive reasoning follow a bottom-up approach where, a tentative hypothesis, is advanced based on patterns observed, and is then, if viable, developed into a full theory of mental workload (Graziano and Raulin, 1993).

## 3. Theoretical Background of Mental Workload

**Figure 8:**
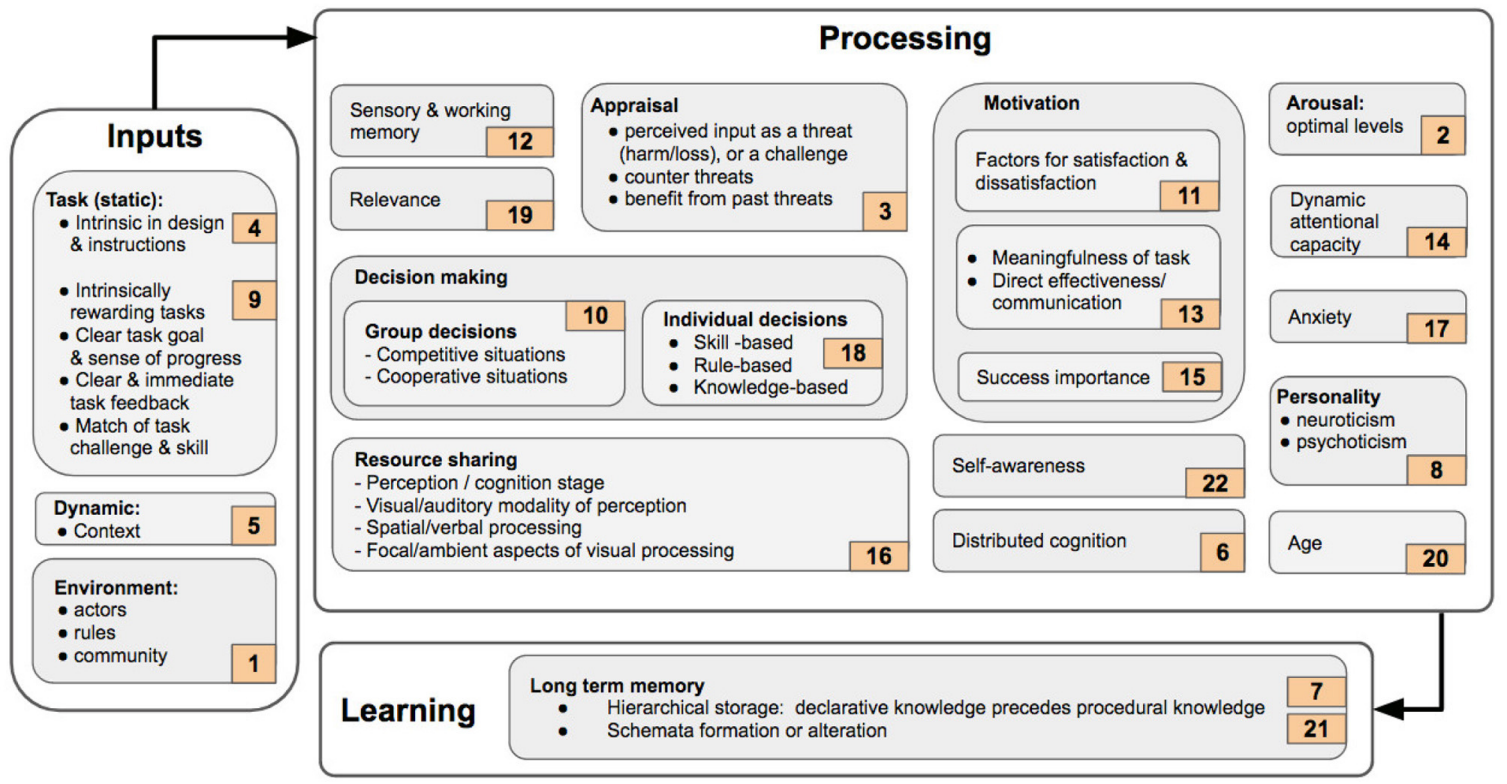
Theories linked to the construct of mental workload organized as a framework.

Mental workload is a complex, dynamic, person-specific, non-linear construct. It is believed by many scholars to be multidimensional (Humphrey and Kramer, 1994; Parasuraman and Hancock, 2001; Recarte et al., 2008; Longo, 2014; Estes, 2015) and intimately connected both to attention (Kantowitz, 2000) and effort (Kahneman, 1973). Many theories proposal exist that have been used to help define, explain, and measure mental workload. These efforts seek to rationalize thinking about mental workload, and are often associated with observational research studies. Theories on mental workload aim to provide a monothetic framework to explain the intrinsic mechanisms and factors that underpin it. Table 1 in Appendix lists the theories that were discussed and/or referenced in the articles considered here, and the seminal work that put forth and explained each theory. The salient features related to mental workload that were discussed or referenced in the corresponding theories, are also described. However, it should be noted that not all the theories identified in the current work, address mental workload exhaustively. Nevertheless, they do address related and relevant factors. Figure 8 collates these different factors into three core blocks of inputs, processing, and learning. In the Figure sub-blocks do not represent any necessary sequential order in which various sub-processes take place. Instead, they show the relations among the different factors that have been established as influencing on mental workload, such as motivation and arousal.

The first block of Figure 8 concerns the *inputs* users perceive. The complexity of a task, its instructions, and its demands are components that, in the terms of *Cognitive Load Theory (4)*, are intrinsic to the task itself (Sweller, 2011). Another model is *Flow Theory (9)* which focuses on circumstances wherein task demands are perceived by the operator to be met by their available resources; a state referred to as ‘flow'. One core characteristic of this state is the extremely high level of operator engagement which is driven by this skill-challenge match, but also bolstered by other task characteristics, such as clarity of goals and feedback, sense of progress and, how intrinsically rewarding the task itself is Csikszentmihalyi (2000). In a wider way, a context is largely driven by the task itself and, therefore, could be considered as influencing the inputs a user can receive. Thus, in turn, effect their response, and experienced mental workload, as put forth by *Contextual Action Theory (5)* (Stanton, 1995). A context is an identifiable configuration of environmental mission-related and agent-related features that help shape behavior (Hoc, 2001). Such factors can be considered static and prior task execution, in the sense that they are thought to be immutable while information while processing task-critical information. However, there are other factors that are dynamic in that they they cannot be anticipated prior to task execution and are stochastic. Examples of such factors include actors, rules, and community in a given environment (Vygotsky, 1980), as proposed in *Activity Theory* (Figure 8, 1).

The second block of Figure 8, the *Processing* block, refers to the way the task-specific inputs are subsequently processed by an operator. These signals are perceived by the sensory faculties, as explained in *Information Processing Theory (12)* (Simon, 1978). Sensory information is then transferred to working memory. Such memory has a limited capacity, and can process and hold only a limited number of bits of information at any given moment. A variety of stimuli may be gathered, experienced, and processed by an operator executing a task. However, the cognitive processing system filters these signals and considers only those that are relevant: a trend central to *Relevance Theory (19)* (Smolka and Pirker, 2018). The input signals are appraised and according to *Cognitive Appraisal Theory (3)*, an input could be perceived as a risk if it threatens the operator's future behavior. In somewhat contrast, a harm/loss is a case in which an operator has already experienced damage in the past (Zajonc, 1984). If a stimulus is perceived as a threat, then working memory processes possible measures to counter it, otherwise it can benefit from it (Zajonc, 1984). Another factor that can influence information processing is motivation. On the one hand, the *Herzberg's Two-factor Theory (11)* presents the hygiene and the motivation attributes, also referred to as factors for satisfaction and dissatisfaction (Herzberg, 1966). Specifically, there are certain independent factors, in the workplace, that can result in job satisfaction or dissatisfaction, and thus in turn can influence mental workload (Simon, 1978). On the other hand, apart from these mechanisms considered of automated feedback, the meaningfulness of the tasks at hand, and the directness and effectiveness of the communication with other participating individuals, have all been identified by the *Job Enrichment Theory (13)* as key factors which influence motivation (Hackman and Oldham, 1976; Cook and Salvendy, 1999). Similarly, according to *Motivational Intensity Theory (15)*, perceived task difficulty and degree of response success can contribute to a person's motivation. In fact, when the importance associated with success is low, then motivation, and consequently effort also drop at lower levels of task difficulty or complexity. Conversely, motivation levels are maintained, despite high levels of task difficulty, if the importance to success is high (Richter et al., 2016). This proposal, when combined with the ideas postulated in *Cognitive Appraisal Theory (3)*, can serve to explain differences in the levels of mental workload experienced by participants during real-world tasks in comparison to simulated tasks (Lazarus and Folkman, 1984; McCarthy et al., 2000). In real-world tasks, operators are more likely to perceive an impending threat. In contrast, in simulated tasks, and a high degree of importance to success, might lead an individual to perceive an impeding threat more as a challenge. Comparative studies that weigh real-world tasks against simulations, such as that reported in Veltman (2002), illustrate that there are clear differences in the physiological responses of humans during real-time tasks.

According to the *Arousal Theory (2)*, an individual's arousal needs to be at an optimum moderate level, neither too low nor to high to facilitate peak performance. Excessively low arousal level results in sleepiness or fatigue, whereas excessively high arousal can lead to stress and anxiety (Cohen, 2011). With regards to task complexity, it was demonstrated that optimum performance was achieved for simpler tasks when the arousal levels were high, whereas for complex tasks, better performance was achieved at a lower level of arousal (Suedfeld and Landon, 1970). Although many additional factors, such as time of day and exogenous stimulants can influence arousal level, the literature focuses on the interaction between the following four factors: i) participant skill ii) task familiarity/past knowledge, iii) personality and iv) task difficulty/complexity (Fontaine and Schwalm, 1979; Revelle et al., 1980; Huber, 1985; Hancock, 1989; Gellatly and Meyer, 1992; Cohen, 2011). The difference between task difficulty and task complexity is that the former is a perceived phenomenon whereas the latter is considered an inherent property to the task. A similar idea is put forth in the *Malleable Attentional Resource Theory (14)*. According to this model, attentional capacity can vary in response to changes in task demand. Thus the negative performance variation, associated to situations of underload, can be justified by the lack of suitable attentional resources (Basahel et al., 2010). Performance can also be influenced by other factors, as explained in the *Processing Efficiency Theory (17)*. This framework explains the influence of anxiety on the performance in a demanding situation is more impactful on processing efficiency than on performance effectiveness. Processing efficiency is defined as performance effectiveness divided by associated effort. Experimental analyses have demonstrated that highly skilled humans can cope with ever higher task complexity levels. However, peak performance for such individuals is also achieved at a higher task complexity in comparison to those less skilled individuals (Revelle et al., 1980; Gellatly and Meyer, 1992). Similarly, it has also been shown that task performance improves as the degree of task familiarity increases (Pena and Quinn, 1997). Optimum performance is consequently reached at higher levels of arousal for familiar tasks in comparison to unfamiliar ones (Fontaine and Schwalm, 1979). With regards to personality, the main factor considered to influence task performance is extraversion (Revelle et al., 1980). Introverts have been found to achieve their optimum performance at lower arousal levels when compared to extroverts. According to *Eysenck's Personality Theory (8)*, there are two other attributes that comprise personality. These are the degrees of neuroticism and psychoticism (Storms and Sigal, 1958). There have been other models that explain personality, and studies have examined the effects of individual personality on task performance (Rose et al., 2002). Others have examined the influence of the dimensions of the ‘big five' or the five factor model (extraversion, neuroticism, openness to experience, agreeableness, and conscientiousness) on task performance (Hurtz and Donovan, 2000). For example, a detailed review has been compiled presenting the impact on seven different aspects of participant personality, namely intelligence, adjustment, extroversion-introversion, dominance, masculinity-femininity, conservation, and interpersonal sensitivity, as well as their relations to human performance in small groups (Mann, 1959). Another important factor which affects mental workload is effort (Garbarino and Edell, 1997). Effort can also influence decision-making (Recarte and Nunes, 2003). Decision-making processes drive the response of an individual to tasks, and they are generally categorized into individual and group norms (Bakr et al., 2008). *Rasmussen's Theory of Skilled Behavior (18)* identifies three levels of expertise, at which participants could act with respect to making task decisions: i) skill, ii) rule, and iii) knowledge-based categories (Woods, 2009). However, the dynamics of decision-making in group scenarios, and the differences between competitive and co-operative groups, are explained by the *Game Theory (10)* (Bakr et al., 2008).

Another important factor influencing information processing is age. According to Salthouse's *Cognitive Theory of Aging (20)*, information processing is affected by age (Bosma et al., 2003) such that various cognitive abilities tend to decline after peaking in the 20–30 s (Fozard et al., 1994; Park et al., 2002). One such cognitive ability crucially linked with mental workload is intelligence, which is often defined as the capacity to acquire and apply knowledge, especially toward a purposeful goal. There are various types including fluid, crystallized (Diggs, 2008) and emotional intelligence, to name only a few (Vickers, 2017). Fluid intelligence refers to that which is used for activities such as problem solving and reasoning, where the need for prior knowledge is largely minimized. Crystallized intelligence uses previously acquired education and skills (Diggs, 2008; Anderson and Craik, 2017). Emotional intelligence concerns the ability to perceive, understand, integrate, and regulate emotions. This parsing of the forms of intelligence represents a theoretical basis for understanding how task performance can be enhanced, and consequently how consequently mental workload is impacted. For example, although aging leads to decline in fluid intelligence, crystallized intelligence remains stable, or can even improve with age. Empirical evidence demonstrates how a significant interaction between task performance and age exists: older adults consistently make more mistakes than younger adults across all levels of mental workload (Vickers, 2017). Similarly, adults with high emotional intelligence are prone to make more errors (Vickers, 2017). These categories of intelligence have been deemed too broad, and several sub-classifications that identify and group specific aspects have bene generated in Johnson et al. (2004), Kane et al. (2004), and Diggs (2008). Another aspect strictly related to mental workload is cognition. Broadly speaking, cognition can be defined as the mental faculty of knowing. Its associated processes include perceiving, recognizing, conceiving, judging, reasoning, and imagining. Cognition is also comprised of constituent components that include intellectual ability, learning, and memory. These abilities have been further divided into more specific components, measurable by various means such as the Comprehensive Ability Battery, the Hawaii Battery and the Weschler Adult Intelligence Scale (Johnson et al., 2004). Abilities, such as cognition and attention are finite and limited. A critical aspect of processing information, using limited attentional resources, is postulated by the *Multiple Resource Theory (16)*. This theory explains the behavior of an operator as he or she concurrently performs multiple tasks that rely on the expenditure of multiple resources shared among these tasks (Wickens, 2002). The theory is comprised of four divisions of resources that can be used for information processing: i) perception/cognition and response stages; ii) visual and auditory perceptual modalities; iii) analog/spatial processes codes, and categorical/symbolic (usually linguistic or verbal), tactile processes codes; iv) focal and ambient vision visual channels. According to this theory, multiple tasks can use different pool of resources, and in case of resource sharing, overload situations can occur, which in turn can impair an operator's performance (Wickens, 2008). Operator performance, with workload optimisation, in turn, may be enhanced with an increase in self-awareness levels. According to *Self-Awareness Theory (22)*, individuals can focus their attention on the self (internal), or on the external environment, at any given moment in time (Hsu et al., 2015). The process of self-evaluation is activated when a person focuses on, and compares the self, with standards of correctness that define the expectation to think, feel, and behave. In turn, this process of self-evaluation enables humans to change their behavior, and to experience pride or dissatisfaction, based on the degree to which they meet their own intrinsic standards (Oldfield, 1954). Another factor that affects information processing, according to the *Distributed Cognition Theory (6)*, is that cognition and knowledge are not attributed to a participant alone. Instead, they are distributed across social groups, the environment, and the time of interaction (Hollan et al., 2000).

The final conceptual category illustrated in Figure 8 is *learning*. This process is connected to the notion of long term memory which has unlimited capacity, and this is where any acquired knowledge is stored (Simon, 1978; Sweller, 2011) According to *Event Perception Theory (7)*, events are perceived and stored in terms of hierarchical structures (Johansson et al., 1980). Here, declarative knowledge precedes procedural knowledge. A similar idea is advanced in *Schemata Theory (21)* which postulates that experience and knowledge that are acquired are stored in the form of building blocks of cognition known as schemas. Learning is thought to take place when new schemas are formed, or existing schemas are altered (Sweller, 2011). Schema and learning are core elements of *Cognitive Load Theory (4)*. According to this theory, the cognitive load that a person experiences can be of one of three types: intrinsic, extraneous, and germane. This theory effectively synthesizes the three blocks of Figure 8 (inputs, processing, and learning). Intrinsic load refers to the demanded effort associated with a specific task, while extraneous load is linked to the way a task is presented (*inputs* block). Germane load refers to the effort and cognitive processing exerted by a human (*processing* block), into the formation of a schema in permanent form of knowledge in long-term memory (*learning* block). These three types of load proceed through a continuous evolution, and it is still not clear whether they are independent, and can be aggregated toward an overall measure of cognitive load (Orru and Longo, 2018).

In summary, many theories exist to explain human behavior and in turn, contribute to the definition of those factors that can affect perceived mental workload, and that in turn, lead to variations in associated performance. As synthesized in Figure 8, a group of these theories seeks to identify various inputs that contribute to mental workload and explain how they influence cognitive processing. These inputs can be static, for instance element related to the design of specific tasks/instructions, or associated with the mental state of an operator prior to task execution. These initially identified factors can be considered immutable during cognitive processing, but they can all influence it. Other inputs are dynamic, such as those related to the context in which tasks are executed. They are dynamic as they are not pre-defined and change during task execution. Thus, they influence human behavior and in turn, perceived levels of mental workload. Another set of theories are related more to the way humans process information. These models are intrinsically associated to the characteristics of an operator such as past experience, ability to process information, and the internal strategies adopted for task execution and resources usage. Similarly, others reflect internal motivational factors such as effort, and the capabilities to cope with environmental influence and interaction with other humans. Another group of theories are focused on investigating the effect of information processing on how learning occurs especially in long-term memory. In synthesis, many theories have influenced the formation of the construct of Mental Workload, each promoting different aspect and influencing factors. On one hand, their aggregation and inclusion in a unified definition of Mental Workload is simply an impossible task. On the other hand, each category of theories provides different meaningful focus on the nature and mechanisms of MWL to enhance understanding, measurement, and regulation of the construct.

## 4. Defining Mental Workload

Defining mental workload has been a major challenge to both theorists and practitioners. This circumstance is especially true given the abundance of theoretical work associated with this construct, the many interpretations of the phenomena, and the contributions from different disciplines. As a result, the term ‘mental workload' is often used to broadly encompassing the demands imposed on users, the effort experienced by operators to meet those demands, as well as the consequences of attempting to meet those demands (Cain, 2007). Although mental workload can be intuitively defined as the total cognitive work needed to accomplish a specific task in a finite time period, it continues to be a challenge to define precisely. Despite many years of research, it remains hard to present a universally/generalisable and acceptable definition of mental workload (Longo, 2014, 2015). This shortfall has likewise been captured in other literature reviews (Miller, 2001; Cain, 2007). The difficulty in defining mental workload is compounded by the diverse methods of measuring it, its own multi-dimensional nature, and its widespread applicability across operational tasks and environments of interest (Hancock and Meshkati, 1988; Veltman and Gaillard, 1993; Wierwille and Eggemeier, 1993). In the following section, we compile and examine a comprehensive list of definitions of mental workload forwarded by researchers from diverse fields (Table 2 in Appendix). They have been organized based on a semantic analysis. They were grouped together when the underlying semantics of the different definitions were similar.

The two basic entities that are involved in the dynamics of mental workload are specified in the first two definitions (Table 2 in Appendix): a *task* and a *subject*. It establishes mental workload as a *multidimensional* construct that originates from the interaction between these two entities. It also serves to show how mental workload represents the load that a particular task imposes on a particular operator/performer (Paas et al., 2003; Haapalainen et al., 2010). Definitions 3 to 5 elaborate on this proposition by identifying the main attributes of this interaction: task *demands* and operator *performance* (Hancock and Caird, 1993; Byrne, 2011; Colombi et al., 2012). Similarly, definition 6 links the cost incurred by the operator to achieve a specific level of performance (Marquart and de Winter, 2015). Definitions 7–12 specify the cost of this interaction and identifies the elicitation of the *internal cognitive resources* of the operator as the cost incurred during the interaction. These characteristics establish that this cost is determined by the dynamics between the internal/cognitive resources, at the operator's disposal, and those demanded by the task (Haga et al., 2002; Mizobuchi et al., 2005; Leung et al., 2010; Palinko et al., 2010; Liang et al., 2014; Lukanov et al., 2016). In consequence, definitions have helped establish a number of key dimensions in defining mental workload: a task and its demands, the operator performance on said task, and the internal limited cognitive resources necessary for successful performance.

The dynamics of resource sharing, which plays a critical role in determining mental workload, are addressed in definition 13. Herein mental workload is described in terms of costs incurred by an operator while performing multiple tasks that use a common pool of resources (Wickens, 2002). Definitions 14–31 provide greater clarity and precision regarding specific additional attributes pertaining to mental workload. In detail, these are *time, cognitive capacity, information processing capacity, mental effort* and *memory*. Definitions 32–47 describe mental workload by establishing a quantifiable relationship between these attributes. In particular, they are based upon the notions of limited information processing capacity or limited cognitive resources, to meet task or system demands. Definition 48 describes mental workload in terms of the degree of the operator engagement with the task (Verwey, 1990; Weinger et al., 2004). It can be argued that the degree of expenditure of internal resources can be considered a reasonable representation of operator engagement. This is further stressed in definitions 33–36 that specify mental workload in terms of the degree to which the internal resources of the operator are used while engaging with the task (Miller, 2001; Weinger et al., 2004; Young et al., 2008; Lim et al., 2015; Wang et al., 2016). On the one hand, definitions 37 to 41 explicates mental workload as a difference between one or more of the aforementioned resources at the operator's disposal, and those demanded by the task (Young and Stanton, 1997; Kum et al., 2007; Lin et al., 2011; Omolayo and Omole, 2013; Harriott et al., 2015). Similarly, definitions 42 to 44 specify mental workload as the proportion of the total mental capacity, that is used at a given moment, to meet the task demands (Alexander et al., 2000; Pierce, 2009; Borghini et al., 2014). On the other hand, definitions 45 to 47 are based on the belief that mental workload can be represented as the ratio of the internal resources available at the operator's disposal, to those required for the task under execution (Haga et al., 2002; Saleem et al., 2009; Hu et al., 2016). Another critical point of distinction, is provided by definition 47. The latter describes mental workload as the ratio between the operator's processing power and the input coming from the *environment* (Frey et al., 2013). The distinction here lies in the use of the word “environment”, as opposed to “task,” implying that there could be stimuli from the environment other than those originated from task itself. For example, students in a noisy classroom are likely to have a higher mental workload in comparison to students in a quiet classroom, despite the fact that the learning task for these two groups is exactly the same (Becker et al., 1995).

One commonality across all the aforementioned definitions of mental workload is the lack of consideration of overload circumstances. That is situations in which the resources demanded by a task are no longer a portion of the operator's mental resources, but actually exceed them. The key distinction here is that the aforementioned definitions establish a mathematical relationship (in terms of differences, proportions and ratios), as opposed to an abstract one between the operator resources and imposed task demands. The idea of expressing mental workload, in terms of differences and ratios is useful from a practical standpoint because it provides equal emphasis on the resources available at the operator's disposal and those demanded by the task. Haga et al. (2002) and Saleem et al. (2009) make an interesting point which could help consolidate these different factors under larger umbrella conceptualization. In their definitions (49 and 50) they argue that mental/cognitive effort, memory, cognitive and information processing capacity can be grouped under one unified aspect, referred to as human attentional resources. This is because when attention is directed at any object or entity, it naturally invokes the use of memory, cognition, and internal processing, and therefore it demands some mental or cognitive effort. Definitions 51 to 57 take the dimension of *time* into consideration, along with the other dimensions discussed above (Brown and Boltz, 2002; Wickens, 2002; Carswell et al., 2005; Kum et al., 2008; Byrne et al., 2014; Longo, 2016; Rizzo et al., 2016; Moustafa et al., 2017). The criticality of time is expressed clearly in Carswell's definition which describes mental workload as the ratio of the mental resources required to the total resources available, on a moment-to-moment basis (Hancock and Caird, 1993; Carswell et al., 2005). This is a key distinction because, it clearly establishes that mental workload varies over time, when the task demand fluctuates on a moment-to-moment basis (Hancock, 2017). Definitions 58 to 61 identify further factors, other than those already discussed, that could influence the level of attentional resources used. These factors include: *situation* (Verwey, 1990; Parasuraman et al., 2008), *task difficulty* (Staal, 2004), *operator skill* (Staal, 2004), and *operator's past experience* (Young and Stanton, 2001). Task difficulty is a factor associated with any task, and thus more than the operator's attentional resources. However, as already discussed, task difficulty can also depend on the operator's perception of the task, as much as its inherent complexity. Therefore, perceived task difficulty is associated with the operator, whereas task complexity is linked to nature of the task itself. Definition 62 alludes to this point when defining mental workload as a concept that serves as an intermediary between imposed and perceived demands (Hancock and Caird, 1993; Young and Stanton, 1997).

An additional element is the analysis of task execution across a group of individuals over a period of time (definition 63) (Xie and Salvendy, 2000b). This is a clearly distinct addition to the other definitions according to Game Theory, an operator's decision-making while executing a task, within a group of people, is different than when executing it individually (Bakr et al., 2008). Definitions 64 and 65 stress this aspect of decision-making from the operator's perspective (Smiley, 1989; Miller, 2001). Specifically, the difficulty and rate of making decisions, along with the rate of information processing, are key factors that mediate mental workload (Smiley, 1989). Definitions 66 and 67 focus on factors that could be considered to comprise the task demands and an operator's performance. Definition 67 expands on task demands by specifying three types: physical, temporal, and environmental demands (Neill, 2011). Finally, definition 68 characterizes mental workload with attributes related to overload. It also describes the consequences that could result if the task demands exceed the operator capacity (Potter and Bressler, 1989).

**Figure 9:**
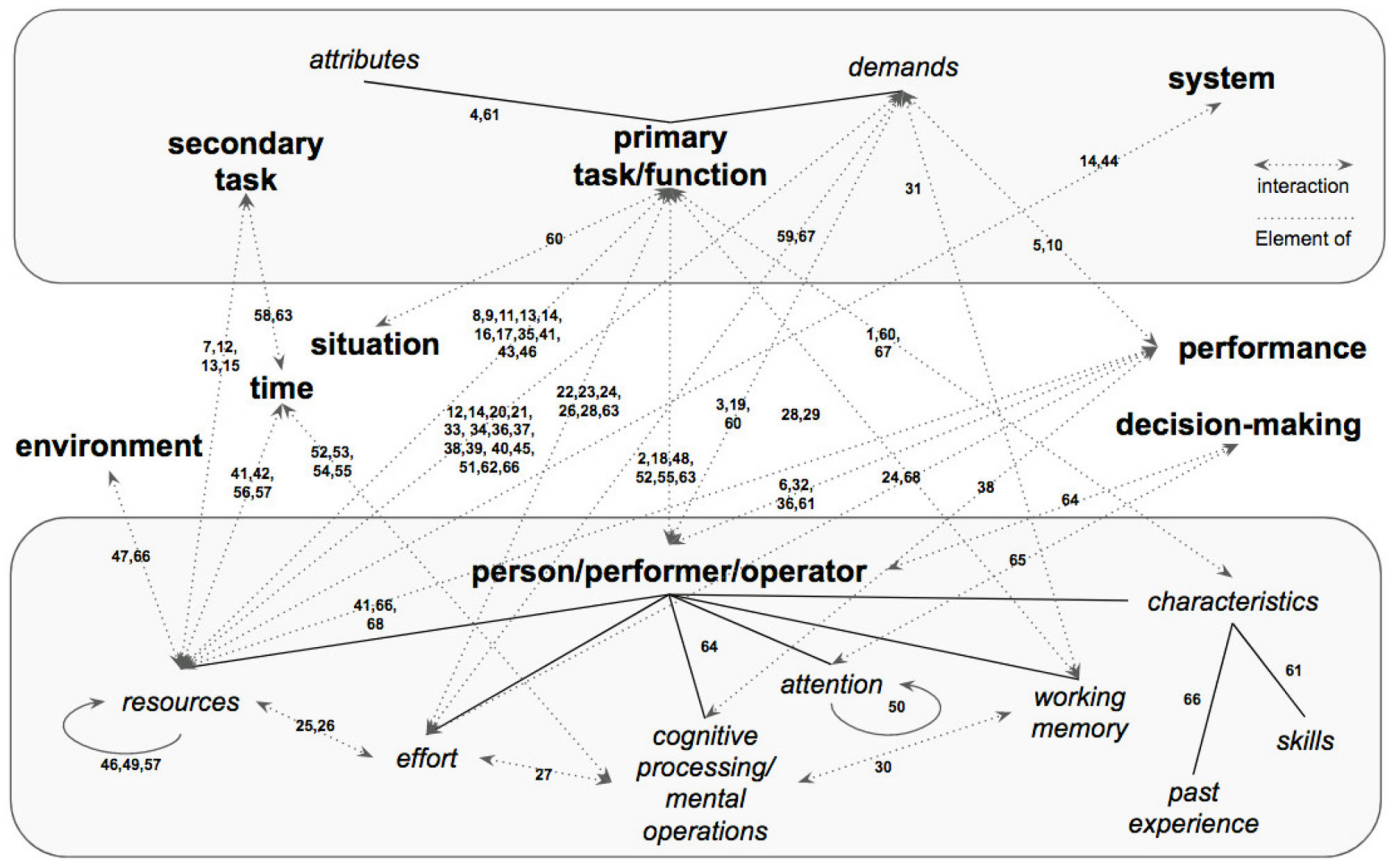
Synthesis of the main concepts, sub-concepts and their interaction associated with the definitions of mental workload found in the literature. The numbers on top of each arrow refers to one or more definitions in Table 2 in Appendix.

In sum, the definitions of mental workload that are found in the literature, are built around a specified number of core concepts and their interaction. Figure 9 synthesizes these concepts and their sub-concepts, with arrows indicating their interaction. The set of numbers, above any arrow, refers to one or more of the definitions provided in Table 2 in Appendix. Firstly, a *primary task* and a *person* are the central notions behind each definition of mental workload. A person, sometimes referred to as *operator*, is the *performer* of a primary task, whose complexity and difficulty can be defined by the concept of *attributes* which require different *demands*. Primary tasks are usually executed in the context of a specific *system*, which can include additional *secondary tasks*. Next, the mental capacity of a performer is limited and, it is composed of a finite number of *resources* that can be invoked to cope with task demands and, thus, to perform the primary task. Similarly, an operator has limited *working memory* to be used during task execution. In sequence, the interactions between a person and a task is not a stationary one, dynamically extending across *time*. It is influenced by the *characteristics* of an operator such as *skills* and *past experience*. During *cognitive processing*, a person executes a number of *mental operations* that are influenced by relevant *attention* and *effort*. In turn, these operations are mediated by the influence of the *environment* in which the task is executed and, the *situation* in which the performer is involved in. These external mediators, along with the internal dynamics of a person, eventually lead to a certain level of *performance* which is the dependent variable that usually needs to be predicted. Performance, in turn, influences and is influenced by human *decision-making*. Definitions vary according to the field of application derived from the orientation of the proposers and their disciplines. Clearly, then a universally accepted definition of mental workload does not presently exist, leaving researchers and scholars to continue to work on this question and propose new definitions that are more precise and inclusive.

## 5. Measuring Mental Workload

It is widely understood that there are three main classes of measures of mental workload: self-report measures, physiological (and neurophysiological) measures, and primary task performance measures. A number of systematic reviews have been already published in this space, including (Kramer, 1991; Cain, 2007; Morris et al., 2007; Whelan, 2007; Antonenko et al., 2010; Byrne, 2011; Lean and Shan, 2012; Marquart et al., 2015; Young et al., 2015; Butmee et al., 2018; Orru and Longo, 2018; Charles and Nixon, 2019; Tao et al., 2019; Hancock et al., 2021; Marchand et al., 2021; Pagnotta et al., 2021). Therefore, conducting a new systematic review for mental workload measures not only is not feasible, but unnecessary. Despite this, we believe that understanding the state of the art in mental workload measurement is necessary to inform a framework that can guide future research. For this reason, a description of each of these classes of measures, and their distribution across the identified articles considered is provided below.

### 5.1. Self-Report Measures

Self-report measures, often referred to as subjective measures, involve a participant or “subject” who usually provides qualitative and/or quantitative reports concerning his/her personal experience while performing either a primary, or secondary task or both (Moray, 1982; Vidulich, 1988; Nygren, 1991; DiDomenico and Nussbaum, 2008; Moustafa and Longo, 2018). In many self-report measures, a user is asked to answer a pre and/or a post-task questionnaire. This strategy aims at identifying possible biases in performance that an operator might exhibit due to their pre-task mental state. Most subjective measures are administered post-task and can be further sub-classified as: i) uni-dimensional, ii) hierarchical, and iii) multidimensional ratings. Uni-dimensional self-reports generally provide a single summary value, usually reported via a numerical/categorical scale with different ranges, provided either in written or verbal form. Although this approach is extremely simple from a data acquisition perspective, as they are non-intrusive, some believe these ratings lack structure and provide limited or sparse information at best for diagnostics purposes (Hart and Wickens, 1990). However, others have demonstrated that they may have good diagnosticity for task demands (Tsang and Velazquez, 1996; Rubio et al., 2004; Longo and Orru, 2019). Uni-dimensional scales represent the concept of workload as one continuum, and examples include the Rating Scale Mental Effort (Zijlstra, 1993) and the Instantaneous Self-Assessment Workload (Tattersall and Foord, 1996). In hierarchical ratings, operators make a set of decisions, and each answer leads to another choice or to a final numerical rating (Hart and Wickens, 1990). Examples of hierarchical ratings include the Modified Cooper Harper Scale (Wierwille and Casali, 1983) and the Bedford Scale (Roscoe, 1987).

Multidimensional ratings operate under the assumption that component factors can be evaluated by operators more reliably than a global summary assessment. Unlike uni-dimensional ratings, these measures provide diagnostic information about the specific sources, as well as providing a global summary (Hart and Wickens, 1990). Examples of multidimensional ratings include the NASA-TLX (Hart and Staveland, 1988; Hart, 2006), the Workload Profile (Tsang and Velazquez, 1996) and the Subjective Workload Assessment Technique (SWAT) (Reid and Nygren, 1988). Table 3 in Appendix lists all of the subjective measures that were used in the articles considered in our review. Although this is is not an exhaustive list, it is representative of the distribution of self-reported measures across different works. As indicated in Table 3 in Appendix, the NASA-TLX is the most used measure to assess mental workload, followed by the SWAT, the Rating Scale Mental Effort and the Workload Profile. Such multidimensional scales have been used across many disciplines, sectors, and domains of application. This is mainly due to their ease of use, and their obvious recognition in this field of research (Cain, 2007). Multidimensional ratings are generally considered to have high sensitivity and diagnosticity, low levels of intrusiveness and convergent validity, as well as moderate concurrent validity (Miller, 2001; Rubio et al., 2004; Fréard et al., 2007). However, certain associated problems have been identified. For instance, the SWAT scale, which is supposed to operate under the assumption of conjoint analysis, violates that assumption in various places (Dey and Mann, 2010).

### 5.2. Performance Measures

Performance measures are used to index mental workload based on the operator's level of task completion efficiency. Although the exact relationship between operator performance and workload has not yet been unequivocally identified, it is generally accepted that the performance of an operator can be maximized by optimizing mental workload (Mitchell, 2000). There have been numerous experimental attempts to objectively quantify the relationship between mental workload and task performance (Paas and Van Merriënboer, 1993; Smith-Jackson and Klein, 2009; Marinescu et al., 2016; Longo, 2017, 2018a; Orru and Longo, 2019). Performance measures can be classified into two broad categories, namely primary task and secondary task measures. Primary task measures represent a direct index of performance, and they have considerably high levels of accuracy in measuring long periods of mental workload (Longo, 2015). They are almost exclusively associated with an operator's capacity on the primary task (Miller, 2001). The key limitation of these measures is their inability to distinguish the source of variations in mental workload, when multiple tasks are executed simultaneously. Due to this limitation, some researchers consider primary task measures somewhat unreliable when used in isolation (Longo, 2015). Additionally, primary performance can be influenced by other non-workload factors (Wickens et al., 2015). This gap can be addressed through secondary task performance measures similarly considered as a metric of an operator's spare mental capacity (Carswell et al., 2005; Wickens, 2008). Therefore, these measures can discriminate between the variations in mental workload due to different influences. However, the main drawbacks of secondary measures is that they are considered intrusive enough to influence the primary task performance, and they are sensitive only to large changes in mental workload (Longo, 2015). Unlike subjective and physiological measures, performance measures recorded in the selected peer-reviewed articles, varied according to the specificity of the domain and experimental tasks. Response time, task completion time, performance efficiency, task engagement, task accuracy and error rate were the most common performance measures observed.

### 5.3. Physiological and Neurophysiological Measures

Physiological measures involve the assessment of mental workload through the analysis of physiological responses of an operator while executing a primary task (Hancock et al., 1985; Kramer, 1991; Backs, 1995; Miller, 2001; Hirshfield et al., 2009a; Miller et al., 2011; Hogervorst et al., 2014). This area of mental workload assessment has seen significant progress in recent years. A number of categories of physiological measures have been identified in the sample articles reviewed. These include electrocardiac and cardiovascular measures, respiration measures, ocular measures, neuroendocrine measures and speech measures. Also the development of neurophysiological measures based upon brain activity, have been seen a recent growth. A brief introduction to each of these measures is provided in this section (Charles and Nixon, 2019) and comprehensive list is provided in Table 4 in Appendix.

The most commonly used *electrocardiac and cardiovascular measures* are heart rate (HR), hear rate variability (HRV), and blood pressure (BP) (Meshkati, 1988; Henelius et al., 2009). This observation follows the same trend reported in this current review. It is generally understood that heart rate increases when experienced experienced mental workload increases. However, while measuring mental workload, it is critical to ensure that physical load remains more or less constant because, increments in physical load almost inevitably increase heart rate. Heart rate variability measures the variability in time between subsequent heartbeats. Blood pressure is less commonly used than other cardiac measures, mainly due to its intrusiveness (Miller, 2001). Heart rate and its variability have been proved less intrusive and more sensitive to changes in mental workload. However, they are readily influenced by factors other than just the mental workload necessary for primary task execution (Cain, 2007).

Another category include *respiratory measures* such as the respiration rate which denotes the number of breaths per unit time. Generally, it increases as the mental workload increases (Miller, 2001; Lean and Shan, 2012). Similarly to heart rate and its variability, respiratory rate is easy to measure and can be minimally intrusive. During experimental work, the physical load should remain constant in as much as is feasible, as change in it will also have an impact on respiratory rate. An additional reported measure is oxygen consumption which also appears to have a generally positive linear relationship with mental workload (Cárdenas-Vélez et al., 2013).

The category of *ocular measures* is well-established and it is based on eye activities including: blink rate, blink closure rate, gaze angle, pupil size, diameter and pupillary responses (Marquart et al., 2015). Blink rate is the frequency of eye closures in a given time period, whereas blink closure rate is the time spent while blinking (Miller, 2001). The main drawback associated with these measures is the difficulty in isolating the effects of visual workload from mental workload (Hancock et al., 2005). However, some have suggested that these measures are only effective at estimating visual workload, and they are very vulnerable to environmental changes (Miller, 2001). Pupil diameter increases with increasing mental workload, and it is sensitive to a number of demands and emotional states. However, a key drawback is that it is unresponsive after overload occurs and is highly sensitive to any changes in environmental illumination (Cain, 2007). Another prominent measure is electrooculography (EOG). This method measures the electrical potential between electrodes placed on facial muscles that contribute to the control of the eyeballs to determine eye movements. A drawback with EOG is that, in some cases, it is hard to distinguish between rapid eye movements and eye blinks (Verwey, 1990). This method has the same limitations as those associated with blink rate, as it is hard to distinguish between the impact of visual workload and fatigue, from that of mental workload (Borghini et al., 2014).

Neuroendocrine measures are more rare. Salivary cortisol has often been associated with mental workload measurement and has reliably been experimentally shown to reflect levels of mental workload (Fibiger et al., 1986). Our findings indicate that few researchers are likely to use this measure. This is probably due to its main drawback of sensitivity, only changing when the primary task demand increases. It does not show much variation, generally exhibiting low sensitivity, in the case of simple tasks (Fibiger et al., 1986). It is increasingly used to measure stress, which is itself often associated to the construct of mental workload (Cinaz et al., 2013). Although increasingly utilized in the literature, salivary cortisol remains in its growth phase, while new ideas are being promoted in this area of research. For example, it has been proposed that salivary amylase activity can be used as an index of mental workload of a ship's navigator while in control of a ship (Hama et al., 2009). Another study, based on Flow Theory, addressed the phenomenon by which users experienced an implicit addiction to the target activity leading to neglect of other significant social activities. This particular psychological state is characterized by behaviors such as intensely focused concentration, loss of reflective self-consciousness, a deep sense of control, distorted temporal experience, and most importantly, the activity feels inherently rewarding, as in the context of gaming (Keller et al., 2011; Sawyer et al., 2021). This phenomenon is at least partly result of the compatibility between the operator's skills and task demands, and this skills-demands-compatibility also had an influence on stress which was demonstrated by the relatively high levels of salivary cortisol recorded (Keller et al., 2011).

Another category includes *skin measures*. Measuring temperature on different regions of the body is also a well-known method of assessing mental workload. Hancock asserted that auditory canal temperature can serve to reflect global changes in mental workload, and this measure could be used, despite its limitations relating to the inertia of the signal (Hancock et al., 1985; Hancock, 1988). Similar to salivary cortisol, the concept of measuring mental workload based on temperature has not been used as extensively as other physiological measures despite its prolonged existence. However, there are a number of experiments that have used different aspects of human body temperature and have been conducted over the last decade. For instance, it has been proposed to use the Nasal-Forehead (N-F) temperature as an effective index to evaluate a navigator's mental workload (Murai and Hayashi, 2008). The experiment that was conducted with this index concluded that the nasal temperature exhibited the broad trend of a navigator's mental workload as effectively as than heart rate variability. However, the latter was better at registering quick responses of mental workload variation (Murai and Hayashi, 2008). Itoh has experimentally showed that the temperature of the nose tip decreases when the operator engages in a secondary task (Itoh, 2009). The experiments performed by Kajiwara also showed that monitoring facial temperature, along with electrodermal activity, was effective in measuring mental workload (Kajiwara, 2014). Ohsuga observed a drop in skin temperature and an increase in heart rate when participants experienced stressful task instructions. Skin temperature reflects peripheral sympathetic nervous system activity which is activated by mental strain, and therefore, it could serve as a viable option to assess mental workload. However, a significant issue in using skin temperature is that it can also be influenced by changes in environmental temperature. Moreover, forehead skin is not as sensitive to various kinds of strain as the skin of the nose (Ohsuga et al., 2001). Apart from skin temperature, several other physiological measures have been developed and identified as potential indices of mental workload. These include electrodermal activity and galvanic skin conductance response (Pierce, 2009; Fritz et al., 2014; Zhang et al., 2014). Electrodermal Activity (EDA) can be classified into phasic and tonic measures. The tonic element of the EDA signal is considered the baseline of skin conductance. The phasic part is the temporary increase in conductance over baseline levels across the performance of a specific task. Phasic signals could be further classified into specific and non-specific categories. Specific signals refer to those that are caused by an exposure to an identified stimulus. Non-specific EDA signals are found to have a weak link with mental workload, and there have been experiments where a correlation has been observed between these measures and operator response time (Pierce, 2009). Due to these factors, EDA has been adopted as a physiological measure only in a few situations.

As shown in Table 4 in Appendix, the category of *neurophysiological measures* is the most utilized in mental workload assessment. The reason for this wide acceptance may be due to the fact that EEG relies on direct measurement of signals from the brain, rather than indirect measurement of other physiological responses initiated by the brain (Miller, 2001; Murata, 2005; So et al., 2017). As noted earlier, Cain suggested that EEG might not be a suitable workload measure for field studies owing to the requirement of sophisticated signal processing equipment (Cain, 2007). However, it has been recently observed that, with advances in sensor-based technologies, this is no longer as impactful a limitation as once it was. For example, the field of passive brain-computer interfaces (passive-BCI) is focused on assessing and interpreting changes in the user state during Human-Computer Interaction (Zander et al., 2010; Zander and Kothe, 2011; Zabcikova et al., 2022). In particular, in the context of neurophysiological measurement, passive-BCI algorithms and biosignal acquisition procedures have allowed the identification and quantification of relevant mental and emotional states of humans. Although technically challenging, these procedures aims to function in ecological, operational, daily life settings, especially for the real-time categorization and evaluation of mental states and those brain dynamics experienced for cognition (Aricò et al., 2018). Passive-BCIs have been used to detect levels of mental workload in real operating environments, including real traffic conditions (Kohlmorgen et al., 2007). Here, mental workload is often induced through the manipulation of task difficulty, and no other aspect of an operator's state is considered. However, different human experiences can occur, even for the same task difficulty, leading to different cognitive states such as various levels of mental workload. One way of assessing these cognitive states is via EEG bands. EEG signals are usually classified into five bands, depending on their frequency: Delta waves (0−4 Hz), Theta waves (4−8 Hz), Alpha waves (8−13 Hz), Beta waves (13−39 Hz), and Gamma waves (>40*Hz*). The ranges associated with these bands can vary slightly according to different standards and applications. Delta waves are generated during deep dreamless sleep, as well as states with loss of body awareness. Theta waves are most prominent during deep meditation and relaxation, for example in the Rapid Eye Movement (REM) phase of sleep. Alpha waves are associated with calm and relaxed, yet alert states. Beta waves are most prominent during active processing, thinking or concentration, cognition, and arousal. Eventually, Gamma waves are observed with higher mental activity, including consciousness, perception, and problem solving. In relation to mental workload, a general observation is that Beta waves increase and Alpha waves decrease as mental workload increases (Hankins and Wilson, 1998). In their review, Frey and colleagues noted that the Alpha band is associated with attention, and the amplitude of these waves increases when a participant experiences fatigue, or when eyes are closed (Frey et al., 2013). A work studied the impact of task demands, age, and working memory load on EEG signals (Borghini et al., 2014). It was observed that younger adults experienced an increase in Theta activity in the frontal mid-line of the brain in response to increased task difficulty, whereas older adults did not experience this same increase. On the other hand, older adults showed a decrease in Alpha activity in widespread areas across the brain, whereas younger adults demonstrated decreased Alpha activity only in their parietal area. Alpha activity, in the parietal lobes, decreases with an increase in working memory load (Borghini et al., 2014). Another indicator of mental workload is represented by the Theta to Alpha ratio (Di Flumeri et al., 2018). This ratio is computed using the Theta band over the EEG frontal channels, and the Alpha band over the EEG parietal channels (Gevins and Smith, 2003; Aricò et al., 2016b; Borghini et al., 2017). A three-level N-back test was run with participants using a fully mobile self-mounted EEG device Kutafina et al. (2021). Findings demonstrated the potential of such setup for detecting changes in cognitive load, as reflected by alterations across lobes in different frequency bands. In particular, it was observed that a decrease of occipital alpha and an increase in frontal, parietal and occipital theta was associated to an increasing cognitive load. Variations in the theta EEG power spectrum was used as an index of mental workload for army drivers performing combat and non-combat scenarios in a light multi-role vehicle dynamic simulator (Diaz-Piedra et al., 2020). In detail, theta EEG power spectrum in the frontal, temporal, and occipital areas was higher during the most complex task conditions. An evaluation of the alpha-to-theta and the theta-to-alpha band rations were investigated as indexes of mental workload (Raufi and Longo, 2022). In details, authors demonstrated the richness of the information in the temporal, spectral and statistical domains extracted from these indexes for the discrimination of self-reported perceptions of mental workload over two task load conditions.

Other methods to tackle the problem of mental workload modeling and assessment exist. For example, in (Qu et al., 2020), Independent Component Analysis (ICA) was performed to obtain components from which energy features are extracted and used for classifying different task conditions. Another study utilized features representing intra-channel and inter-channel information to classify multiple classes of task load conditions based on EEG (Pei et al., 2020). Multi-frequency power spectrum and functional connectivity (FC) were employed for the classification of two task load levels in two working-memory tasks performed by healthy participants (Kakkos et al., 2021). Beside achieving good accuracy, the spectral and localization properties of designated features revealed common task-independent patterns in the neural mechanisms governing workload. A study tried to tackle the issue of cross-task mental workload generalization, and a cross-task performance-based feature selection coupled with a regression model, that was trained with data gathered from a working memory task, was developed (Ke et al., 2014).

Another study employed microstates and a newly proposed dynamic brain network analysis method based on it to explore the changes in dynamic functional connectivity properties over four task load conditions (Guan et al., 2022). Six microstate topographies labeled emerged and were used to describe the task-state EEG dynamics. A dynamic brain network analysis revealed that a number of nodes and pairs of connectivity from the Frontal-Parietal region were sensitive to mental workload in all the four conditions, demonstrating how these nodal metrics can contribute to the assessment of mental workload in the cross-task scenario.

EEG is often used in conjunction with subjective measures and machine learning classifiers to predict mental workload (Laine et al., 2002; Arico et al., 2015). For example, SWLDA (StepWise Linear Discriminant Analysis) has been used to select a low number of EEG spectral features to aid in Air Traffic Management (Arico et al., 2015). In a similar approach, the features selected by SWLDA were fed to a non-linear Artificial Neural Network (ANN) in order to classify different levels of mental workload (Laine et al., 2002). This latter approach takes advantage of SWLDA's ability to identify features, and of the ANN to attain good predictive accuracy (Laine et al., 2002). Other works have addressed the shortcomings of EEG-based mental workload estimation from the task demand perspective (Ke et al., 2014). A convolutional neural network to classify EEG features across different task load conditions in a continuous performance task test was created in Hernández-Sabaté et al. (2022). The goal was to partly measures working memory and working memory capacity, as an indicator of mental workload. Existing studies that focused on estimating workload, based on EEG measures, have generally produced good results for discriminating task conditions, but only for the specific experimental selected primary tasks. Rarely, developed methods for assessing mental workload are generalisable and usable across tasks. The performance of cross-task mental workload assessment based on physiological metrics remains highly unsatisfactory.

A novel neuro-physiological method that is gaining attention in the field of mental workload modeling is functional near-infrared spectroscopy (fNIRS). This is a non-invasive, brain imaging technology that employs low levels of non-ionizing light to record variations in cereblal activity. Through the application of optical sensors placed on the scalp, similarly to electroenchepalography, it records changes in blood flow that can be used to investigate the evolution of brain activation during various tasks. As a consequence, it has been deemed a promising method for the discrimination of various task conditions, each supposed to lead to different levels of experienced mental workload (Sassaroli et al., 2008; Karim et al., 2012; Durantin et al., 2014; Sibi et al., 2016; Li et al., 2019; Parshi et al., 2019; Galoyan et al., 2021). However, as in the case of application of Electroencephalography, the performance of cross-task mental workload assessment using fNIRS remains highly unsatisfactory.

### 5.4. Advantages, Disadvantages of Measures and Their Integration

**Figure 10:**
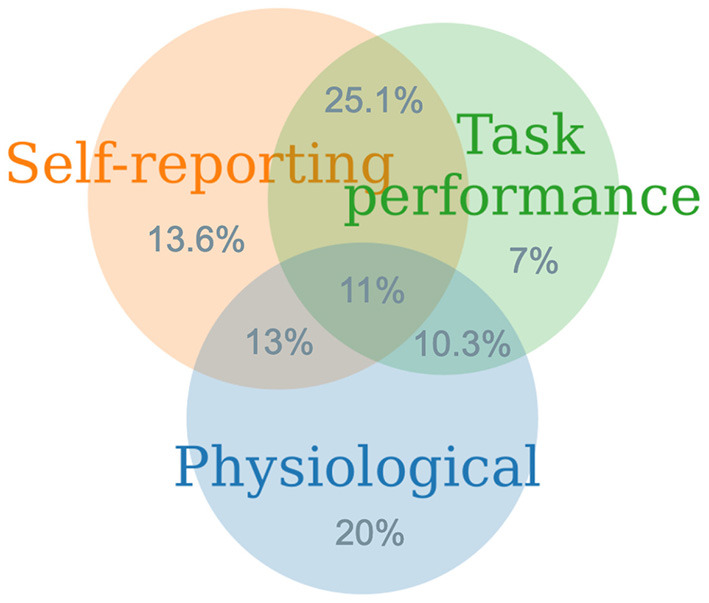
Distribution of the classes of mental workload measures and their joint application.

In summary, each class of mental workload measure is associated with a number of advantages and disadvantages. Self-report measures are widely used, mainly for their ease of administration across contexts and domains. They are also computationally inexpensive, even if they are multidimensional. Thus, they can be easily derived with basic mathematical operators by non-experts. Unfortunately, they are mainly administered post task. They do not interfere with the primary task, and they provide an overall assessment of mental workload, with low granularity over time but also with lower reliability for long tasks. As scores are derived from subjective perceptions, it is difficult to use them for comparison across participants on an absolute scale (Longo, 2014). Task performance measures are the most direct indicators of human performance, and if gathered continuously can be reliable indicators of mental workload, even for long tasks. On the one hand, they possess little diagnosticity in terms of spotting sources of workload and if taken in isolation, they have low utility. On the other hand, if considered in conjunction, they can be useful as dependent variables because they can be explained by other measures. As a result, they would help scholars establish cause and effect relationships. Physiological measures are becoming more widely adopted in experimental settings, thanks to advances in sensor-based and processing technologies. They are very useful at monitoring physiological responses of the body, and neuro-physiological responsed of the brain on a continuous basis, thus representing sensitive measures that often do not interfere with primary task execution. However, they are more expensive to administer than the other classes of measures. This is because the pre-processing pipeline applied to gathered signals is often required to reduce internal and external artifacts, namely those generated by the body, and those coming from the environment. Additionally, pre-processing is often computationally expensive, and it is often conducted offline, thereby limiting their application in real-time assessments of mental workload. Figure 10 synthezises the application of these three classes of measures described above, both individually and jointly. Among these, physiological measures are those most employed individually, followed by self-reports and task-performance measures. This trend is explained by the increasing precision that physiological measures can now reach when compared to the others. Task-performance measures are more difficult to gathered and operationalize in many contexts, and they are not widely applied in isolation. However, their application with self-report measures is the most frequent combination. This is because task-performance measures are most often considered post-task, as a summary of the overall profile of responses. Thus, their correlation can be easily investigated. However, the application of task-performance measure with physiological measures is more problematic, as the former are mainly computed post-task, while the latter are mainly continuous. Thus, understanding their inter-relationship is more arduous (Hancock and Matthews, 2019). Only within the last 15 years has the joint application of the three classes of measures proliferated. Early days of application of mental workload saw mainly individual applications of measures, and only in the most recent decade has combinations of two measures seen evident growth.

### 5.5. Evaluation Criteria for Mental Workload Measures and Methods

**Figure 11:**
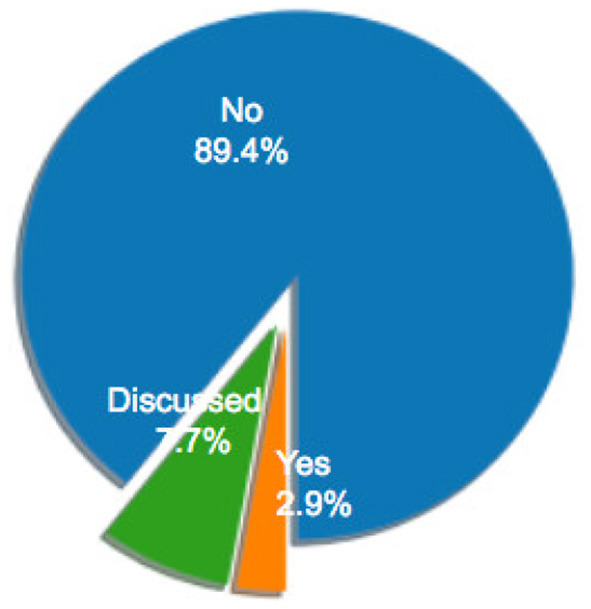
Distribution of the application of the sensitivity criteria.

**Figure 12:**
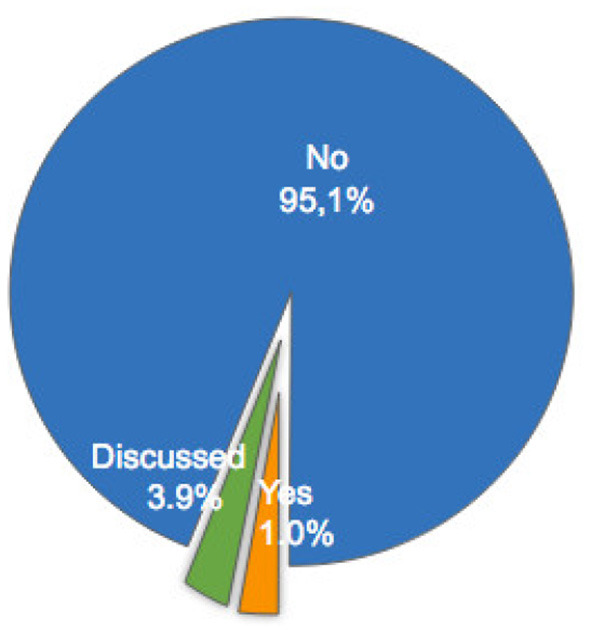
Distribution of the application of the diagnosticity criteria.

**Figure 13:**
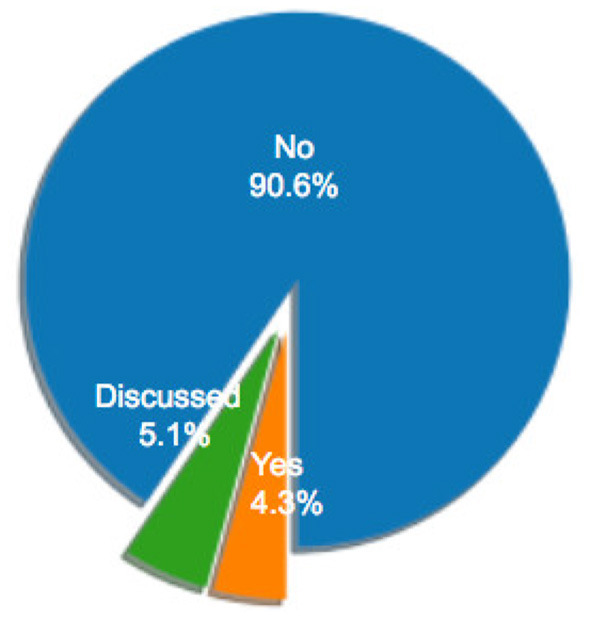
Distribution of the application of the reliability criteria.

**Figure 14:**
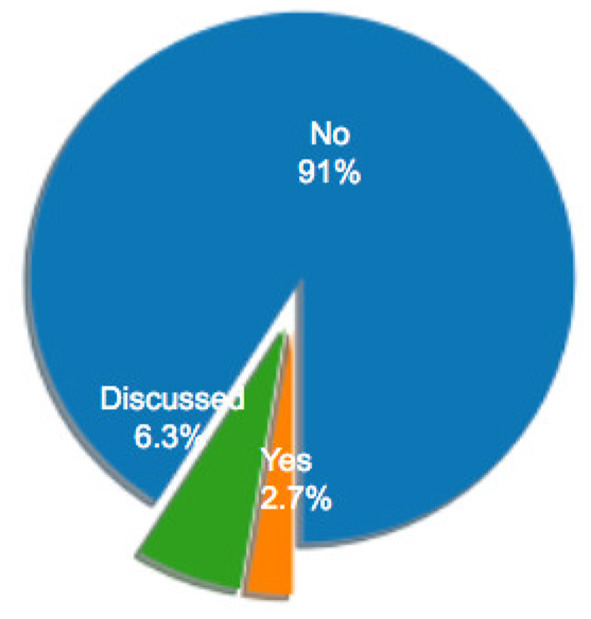
Distribution of the application of the validity criteria.

Several criteria have been used to assess different measures of mental workload. The most frequent are sensitivity, validity, reliability and diagnosticity (Longo, 2018b; Longo and Orru, 2019). Table 5 in Appendix lists the articles that have used and/or discussed these criteria in their experiments and Figures 11–14 depicts the distributions of their application. Tables 7–10 in Appendix list the methods used in these articles to calculate each specific criterion. The characterization of each criterion is designed to evaluate a particular property of a measure of mental workload:

- *Sensitivity* - Here, a measure should be responsive to variations in task difficulty, resource demands, and other factors that potentially influences mental workload. The measure should be able to discriminate between levels of variation in mental workload (Cain, 2007; Moustafa et al., 2017);
- *Diagnosticity* - A measure should be capable of identifying not only changes in workload, but also the causes of said changes by indicating the source of variation (Cain, 2007; Moustafa et al., 2017);
- *Reliability* indicates that a measure shows repeatability, with small variance compared with main effects (Cain, 2007);
- *Validity* - It indicates the actual capacity to measure the intended construct, that means mental workload itself. This could be further divided into component forms. *Convergent validity* is the extent to which a measure to actually assesses what it is supposed to measure, by comparing it to similar measures of mental workload. *Concurrent validity* is the extent of a measure to explain task performance measures (Longo, 2015; Moustafa et al., 2017). *Face validity* is the capability of a measure of mental workload to be subjectively viewed as covering the constructs it aims to measure (Longo, 2014).

Measures are derived from the application of different methods. These methods can be developed by employing certain criteria, and as it emerged from the reviewed sample articles, they are:

- *Agility* - Agile methods should be timely, and sufficiently rapid to apply, as well as capturing transient mental workload changes (Cain, 2007);
- *Intrusiveness* - A method should not be intrusive or interfere with the performance of the operator while executing tasks, and consequently become a significant source of workload itself (Cain, 2007);
- *Requirements* - A method should present minimally intrusive equipment to avoid influencing performance during primary task execution (Longo, 2014);
- *Acceptability* - A method of mental workload should achieve high acceptance from applicable scientific disciplines (Longo, 2014);
- *Selectivity* - A method should be selectively sensitive to factors that affect mental workload, and not sensitive to other factors that are not related to it Cain (2007), Longo (2014), and Moustafa et al. (2017).

Although many criteria exist, it has been difficult to develop a mental workload measure and assessment method that satisfies all of them. Only a small proportion of studies have taken these criteria into account, both for measures and methods. The majority works rely on previous studies, assuming that, if the validity of a measure has already been demonstrated, then that measure can be successfully used without the need to re-evaluate its validity. A primary example would be the NASA Task Load Index a popular multi-dimensional measure without a re-assessment of its validity and other criteria (Noyes and Bruneau, 2007; de Winter, 2014; Grier, 2015). such associated error can be propagated across such studies, making it widely applicable, but not necessarily reliable.

### 5.6. Further Evaluative Approaches

Self-report, physiological, and task performance measures, are the three main categories that fall under the broad umbrella of *empirical measures* (Xie and Salvendy, 2000a). They are empirical as they all involve an ‘operator-in-the-loop' and direct observation of human experience. In contrast, another category is *analytical measures* which are based on analysis or logic (Longo, 2012). The latter can be used without an ‘operator-in-the-loop' for prediction purposes (Xie and Salvendy, 2000a). Analytical measures can be further divided into five sub-categories (Xie and Salvendy, 2000b):

- *Comparative* - Measures that are primarily aimed at estimating task difficulty based on data from prior experience;
- *Expert Opinion* - Measures that project data from comparable systems based on expert opinion. Both comparison and expert-opinion measures are referred to as projective (Xie and Salvendy, 2000a; Rizzo and Longo, 2017, 2018);
- *Simulated* - Measures that are based on simulation models and that take into consideration detailed attributes related, for example, to the task, resources, time and operations among others;
- *Mathematical Techniques* - Measures developed upon a theory or multiple theories such as, control, information or queuing theories;
- *Task Analytical Approaches* - Analytical measures in which the activity of an operator, performing a task, is expressed as a function of time and phase of performance. Such activity refers to a specific set of sub-tasks that an operator needs to perform in sequence, or within a specific hardware system.

Both empirical and analytical measures use various approaches and aggregating functions in order to express their perspective on mental workload. Table 11 in Appendix lists these approaches and strategies that were used in the sample articles considered here.

## 6. Synthesis and Recommendations

The synthetic overview we have presented, while cataloging the current state of the art, permits us also to look toward future research directions. Here, the main objective is to establish the prospects for mental workload and its assessment, in a world ever more dominated by advanced technologies (Hancock, 2014; Longo, 2014). It might seem somewhat contradicting that, just as many of the various techniques to measure workload are being advanced, the potential need for this information is potentially diminishing. However, we believe that this trend is neither necessarily true or necessarily ubiquitous, and mental workload assessment plays a crucial role. It is true that as humans, we stand on the cusp of a radical change in our relationship with advanced interactive technologies. It cannot be disputed that, nowadays, we use many automated systems in which the human contribution is a diminishing one. For example, among others, air-bag deployment systems require no active human intervention at the time of their activation, nor should they for maximum effectiveness. However, despite human contribution and intervention is diminishing, the mental workload experienced by operators can still be high: even boring work can be taxing due to the increase in continued demand of attention by automated systems. There are many other, equally pertinent examples, of where the time-horizon of system response is so far below human response capacities that it is immaterial, and even directly counter-productive to encourage, or advocate any possible form of operator intervention. These are designed to be human-less systems (Hancock, 2020). To discuss the application of human mental workload in such systems is largely without effect. However, humans are still integrally involved in the design, manufacture, and maintenance of such systems, but the active operator here has now sometimes become redundant (Parasuraman et al., 2008; Hancock, 2014). However, not all systems, which employ differing forms of automation remove the human completely from the control loop. It is these that most benefit from the application and assessment of human mental workload. In technological terms, we are in the midst of nothing less than a revolution. There is a degree of reciprocity between control and the nature and function of work itself. At the present, humans still direct much of the ‘actions', in terms of both goal setting and system's operations. However, work functions are themselves often split, or at least spread amongst many human contributors. Similarly, it is often the case that it is not possible to know the cognitive processing limits experienced by any of these individual contributors, without some form of reliable assessment. In this respect, the need for mental workload assessment remains vibrant and vital (Longo, 2014).

The identification of any critical episodes of human shortfall, as revealed by workload assessment techniques, encourages designers to eliminate human participants at this juncture. This is the case since, clearly, the human of the overall system has been shown to fail under the driving forces of extreme operational stress. In this sense, mental workload assessment techniques can be useful as diagnostic instruments, but also the actions taken following their assessment might be too drastic. The rate at which human work loss will be witnessed, in favor of automated machines, such as robots, might well depend upon the ever more accurate assessment of human mental workload. While such concerns must be viewed in terms of the coming decades, the shorter-term needs of accurate workload information will be heavily bound up in the issue of human-automation interaction. For example, contexts that require workload information include the automobile industry, especially with the proliferation of automated and autonomous on-road vehicles (Hancock et al., 2019). It is here that the advancing capacities to measure, and potentially to regulate mental workload, promise to exert their greatest impact. Both the theoretical dimensions of physiological and neuro-physiological assessment, as well as the capacity to apply these measures in the real-world, have expanded beyond all recognition, even across only a limited number of decades (Hancock and Chignell, 1987). Advances in sensor-based technologies have produced tools that can now be used by operators to monitor many of their vital signs, and express brain functions in real-time. Furthermore, through feedback loops, these signs can be employed to regulate imposed task demand.

This trend, especially that of combining real-time primary task performance reflections with an operator's momentary physiological and/or neuro-physiological state, is one which will occupy and challenge workload assessment scholars in the immediate coming decade. Despite considerable research on mental workload, and the increasing number of scientific materials produced every year, a single reliable and valid framework to measure mental workload does not yet appear to exist. Based on the information collected as part of the present literature survey, it seems that one reason for this gap can be attributable to the fact that, all three classes of mental workload measures have been mostly frequently used in isolation, or in conjunction with another class of measures. It is intuitive to argue that these measures should complement each another, and therefore ought to be used together (Charles and Nixon, 2019). However, triangulating findings across different classes of measures is not trivial. In fact, as pointed by Hancock & Matthews, associations, dissociations, and insensitivities among measures exist, and dissociative patterns make mental workload modeling a challenging task (Hancock and Matthews, 2019). In this regard, the issue of dissociation of different mental workload measures has to be recognized and addressed (Matthews et al., 2020; Muñoz-de Escalona et al., 2020). The reasoning is that each of these categories of measures answer only a part of the question, and therefore, correspond to one part of the challenge of measuring mental workload. For example, although subjective measures could be used as the baseline for calibration, in the absence of any other objective measure of task performance (Cain, 2007), self-report measures alone are still only the operator's perception of their mental workload while executing a task. As pointed to the literature, an operator is still largely an unreliable measuring instrument (Cain, 2007). The reason being that it could be difficult for an operator to remember intrinsic, and even explicit details of a task, after executing it. Therefore, this may lead to a lot of considerable subjectivity, bias, and even preconceptions. Self-report measures, as single indices, are believed to have low diagnosticity, as they may not indicate either the source or type of workload (Cain, 2007). Instead, if multi-dimensional and with high reliability, they can lead to the identification of the reasons for a certain overall level of mental workload for a given primary task. However, they are still based on human perceptions that can be influenced by many confounding sources.

Another reason concerning the difficulty in modeling mental workload is represented by its dependency on the attributes of any primary task, such as its complexity and demand. Additionally, its subjective essence complicate modeling efforts as mental workload is a person-specific construct, and it is influenced, for instance, by the skill set, past knowledge, mental capacity and alertness of each individual (Fontaine and Schwalm, 1979; Revelle et al., 1980; Huber, 1985; Gellatly and Meyer, 1992; Liou and Wang, 1994; Makishita and Matsunaga, 2008; Cohen, 2011; Galy et al., 2012). Physiological and neuro-physiological measures are believed to posses a higher degree of sensitivity to changes in workload, especially when compared to self-reports, but they can be influenced by many external or internal factors, other than just those related to a task, hampering their diagnostic power (Cain, 2007; Charles and Nixon, 2019). Primary task measures are not adequate to measure workload since it is difficult to induce changes in task performance when the variability in task demands is minimal. Similarly, they are poor in sensitivity and diagnosticity when other factors, such as strategy and individual subjective mechanisms for cognitive processing, affect performance (Cain, 2007). Primary task measures also run the risk of being dissociated from other measures of mental workload, if the task demands cause fatigue due to long periods of exposure to a primary task (Cain, 2007). Similarly, secondary task measures may themselves induce an undesirable change in strategy, and consequently, may distort primary task performance (Caggiano and Parasuraman, 2004). The above observations allude to the idea of triangulation of the three categories of measures to better understand an operator's mental workload (Charles and Nixon, 2019). This is reflected in the works published across the last two decades, where the number of articles, that use a combination of these categories, has proportionately increased in comparison to only those using one category (Cegarra and Chevalier, 2008). Despite many theories having been conceived and employed to explain different aspects of mental workload, a comprehensive theory has not been agreed. The main reason for this is the volume of knowledge and data acquisition, experimental procedures, and technical skills required to fully explicate a multidimensional construct such as mental workload (Longo and Barrett, 2010a,b). Additionally, to further complicate things, the majority of scholars in various disciplines, not strictly focused on modeling the construct of mental workload, use the term ‘mental workload' to refer to the ‘task demands', ‘difficulty' or ‘complexity' of their experimental task conditions, defined prior the actual recording of the human responses over them, and thus static. For these reasons, a universally accepted definition of mental workload does not exist, despite many decades of research in this field. Standardized, domain-specific procedures, to accurately measure mental workload, are yet to be finalized. This is due to the diversity of factors that affect mental workload, and the measures that have been and continue to be developed in specific fields and disciplines.

### 6.1. A Novel, Operational Definition of Mental Workload

**Figure 15:**
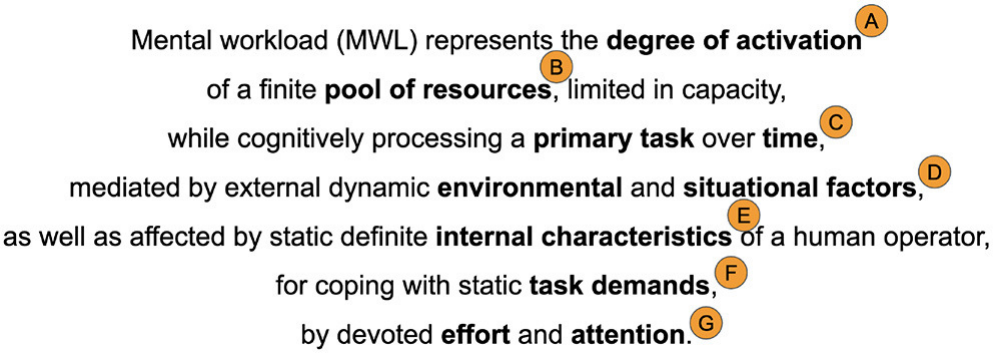
A novel, inclusive and operational definition of human mental workload.

Unless the individuality in the definition of mental workload and the application of measures in different fields does not change, a breakthrough in research is not probable. However, with an attempt to circumvent this tendency, and in order to increase generalisability, a novel definition is presented here. It is achieved by taking into consideration all the concepts that have emerged while doing this literature survey, and by building upon each of the extracted definitions (Table 2 in Appendix). This definition aims to be more inclusive than current ones, but more operationalisable and modelable: *Mental workload (MWL) represents the degree of activation of a finite pool of resources, limited in capacity, while cognitively processing a primary task over time, mediated by external stochastic environmental and situational factors, as well as affected by definite internal characteristics of a human operator, for coping with static task demands, by devoted effort and attention* (breakdown of sub-components in Figure 15).

This novel definition explicitly acknowledges that mental workload is a multidimensional construct which is influenced by differing factors. These include environmental influence and situational factors (D) that originate outside the internal cognitive information processing system. These factors are dynamic and stochastic, and their initiation is not under the conscious control of the performer. However, each individual has different internal characteristics (E) for dealing with these external factors. These include skills and past experience, influencing the amounts of exertable attention and effort (G) required. These person-specific characteristics also affect the execution of a primary task over time (C), leading to the employment of different strategies to cope with such task demands (F). Additionally, the internal cognitive processing system is split into a finite pool of resources (B) that are limited in their capacity and they can be evoked and activated (A) for executing the primary task (C).

The presented definition should be seen as a foundation for the future mental workload research. It contains high-level classes of notions and concepts that can be individually modeled, operationalized and investigated. Firstly, it clearly points to an amount of finite pool of resources (B) that compose the human cognitive processing system. Therefore, research should be devoted to establish this value in a way that the pool of defined resources covers the entire spectrum of functionalities that the brain can perform. An example includes the Multiple Resource Theory (Wickens, 2002, 2008). Each resource is limited in its capacity, suggesting that an upper bound and a lower bound of its activation (A) exist. Such bounds should be defined for each person. The activation of a resource implies that these bounds can be actually quantified, and should be treated as quantitative attributes, that can and ought to be established empirically. For example, by using neuro-physiological measurement techniques, such as EEG, brain responses can be collected over time from an individual operator executing a task, and once properly cleaned from artifacts, the average local maxima and mimina of the Global Field Power (Skrandies, 1990) (or the power at specific scalp locations) could be used as upper and lower bounds respectively. These bounds can subsequently be normalized across humans to support meaningful comparisons. Another class of notions refers to environmental influence (D), the circumstances and situational factors (D) under which a task is performed. Thus, researchers should focus on designing, modeling, gathering and quantifying a set of factors that can influence the execution of the primary task (C), but whose initiation is outside the immediate control of the performer. This is important because it can allow scholars to establish whether temporary shifts in attention to secondary tasks occur and how much they influence the execution of the primary task. Similarly, a focus on the definition of a definite set of quantifiable factors related to the internal characteristics (E) of the human operator should be engaged as these also contribute to the execution of the primary task. These latter factors are often considered static and immutable during the execution of a task. They can indeed evolve after the execution of many tasks across the life of each human, but they are deemed to be immutable for the immediate primary task performance. Example includes skills, prior knowledge and motivational levels. The proposed novel definition of mental workload also explicitly acknowledges time (C) as a critical variable to model. As tasks are executed over a period of time, the quantifiable activation of each resource and each external factors must be considered and modeled continuously across time. The definition moreover includes static task demands (F), suggesting that another class of quantifiable attributes should be formed around the description of the primary task. Their quantification should be considered as immutable over its execution, thus being similar to the static characteristics of a person. Indeed real-world tasks can have varying demands, but since they are often designed for research purposes, thus controllable, these demands are considered static. In case a designer would like to define a primary task with incremental/decremental of varying complexity, s/he can split it into sub-tasks at those points in time where their individual demands actually change. This will allow scholars to specifically investigate how the non-linear level of attention and effort (G) devoted by the human operator, is actually fluctuating, thus understanding coping strategy, especially for prolonged tasks.

**Figure 16:**
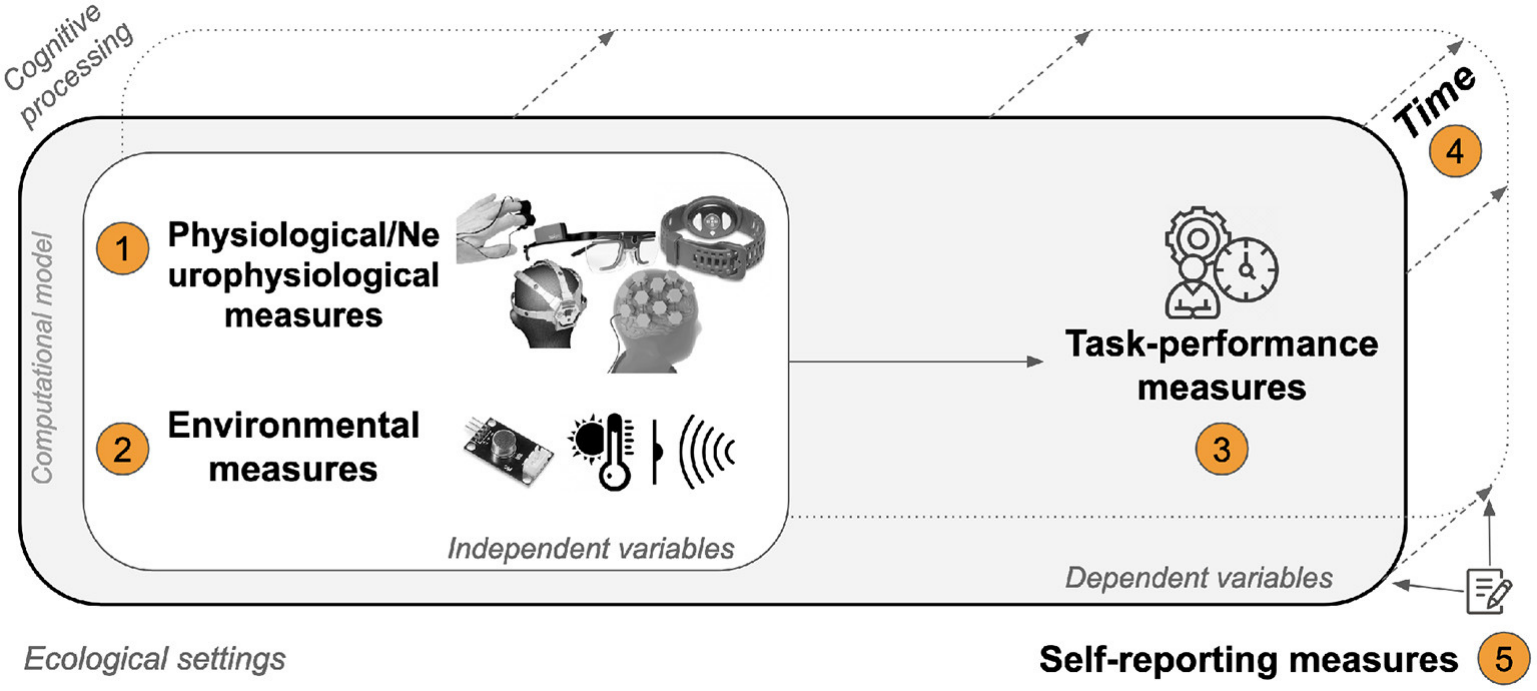
An envisioned framework toward the development of a robust multidimensional mental workload model with high reliability, sensitivity, diagnosticity and validity, with different classes of measures and methods.

The overall rationale behind the presented definition is mainly a practical one. It provides scholars with an operational characterization of mental workload that can be parsed into sub-components that, in turn, can be individually modeled. This is designed to motivate empirical-based research and the use of quantitative methods for greater replicability, reproducibility and falsifiability (Longo, 2014, 2015). Progresses in triangulating measures, theoretical development of hypotheses and testing, are the first step to bringing us closer to solving this important goal (Hancock, 2017; Wickens, 2017). According to this consideration, and taking into account the above interpretations, we propose a five-dimensional framework, as depicted in Figure 16. It aims to provide scholars with recommendation for advancing mental workload. This framework is also centered around our new operational definition of mental workload proposed earlier.

One recommendation is to use physiological and neuro-physiological measures (point 1 in Figure 16), to continuously monitor an operator as these measures are sensitive to changes in workload variations over time (Rusnock and Borghetti, 2018). A parallel can be drawn between mental workload measures and passive (implicit) Brain Computer Interfaces (passive-BCI) since the latter are aimed at assessing and interpreting changes in the user state during the processing of a task. In detail, these measures can help in quantifying the degree of activation of the finite pool of resources that are employable for cognitively processing the primary task. Environmental measures should be added to model development (point 2 of Figure 16) in conjunction with these physiological and neuro-physiological measures because, mental workload can be affected by signals coming from the environment in which a primary task is executed. The increasing availability of tools for measuring brain responses, such as portable, wireless EEG caps, as well as wearable sensors, can facilitate data collection, and the monitoring of physiological and neuro-physiological responses in the field. Similarly, various sensor-based technologies, now available on the market at a low cost, can help continuously gather data from the environment itself. Data gathering, should thus be conducted in ecological settings and not only within laboratories and other highly controlled environments. This is because real-world activities prove more natural, and therefore may represent different behavioral responses that often, are not evident in lab-based experiments. On the one hand, both physiological/neuro-physiological and environmental measures should be used as independent variables. On the other hand, a set of primary-task performance measures should be designed, and used as dependent variables (point 3, Figure 16), since they are the closest form of ground truth that can be objectively gathered. Dependent and independent variables should be continuously gathered across time (point 4 of Figure 16). In turn, cause and effect between these variables can be extracted, and this will support the development of a sensitive measure of mental workload. Self-report measures should be used mainly as a form of triangulation (point 5 of Figure 16). These should be gathered prior to and following task execution in order to avoid confounding the operator's natural execution. A priori self-report measures help define the individual characteristics of an operator, such as motivation, in advance of task execution. Similarly, post task self-report measures help establish the effort and attention devoted to coping with the static demands of such a primary task. These self-report measures support triangulation and facilitate the identification of the circumstances in which the subjective assessments of an operator dissociate with the gathered physiological and neuro-physiological responses. This ultimately allows the development of novel hypotheses which lead to novel findings. These hypotheses should be systematically tested against sensitivity, validity, reliability, and diagnosticity, and only those that maximize these criteria should be retained.

Physiological and neuro-physiological measures can support the creation of an accurate model of mental workload which is variations in task difficulty and resource demands. Along with self-report measures, they can permit the formation of a diagnostic model from which to identify sources and causes of workload variation. For example, electroencephalography-based (EEG) measures, through source localization algorithms, can help identify when parts of the brain and cerebral cortices are activated at a particular point in time. These can then be triangulated to multidimensional self-reports of overall resource activation, such as the Workload Profile instrument (Rubio et al., 2004) grounded on the Multiple Resource Theory (Wickens, 2008). Self-reports measures can also serve to validate and support the formation of a valid model that is also reliable. Regarding data collection, physiological and neuro-physiological methods can meet the agility requirement, and rapidly capture transient mental workload changes. Modern data collection technologies should also allow for the development of non-intrusive physiological and neuro-physiological measures, with minimal equipment and requirements. They can accompany self-reporting methods which already have high acceptance, low intrusiveness, low practical requirements and are receptive to factors that actually influence mental workload. Similarly, task performance measures have good selectivity, low intrusiveness, and often require minimum equipment. In this regard, future work should be focused on the development of a taxonomy of task performance measures, aimed at covering a wide range of real-world tasks and establishing a reference that can be used across empirical studies by different domains. Over systematic empirical research attempts across these tasks, and by employing the three classes of measures, a growing set of valid hypotheses may be formed, and a more generally applicable and consistent multidimensional and prospective model of mental workload can be generally agreed.

## Author Contributions

LL designed the study, conducted the literature review, created the figure, and reviewed the manuscript. CW provided advisory feedback on the manuscript. PH provided advisory feedback, contributed to the article, and reviewed the manuscript. GH provided comments on all the sections, reviewed, and proof-read the manuscript. All authors contributed to the article and approved the submitted version.

## Conflict of Interest

The authors declare that the research was conducted in the absence of any commercial or financial relationships that could be construed as a potential conflict of interest.

## Publisher's Note

All claims expressed in this article are solely those of the authors and do not necessarily represent those of their affiliated organizations, or those of the publisher, the editors and the reviewers. Any product that may be evaluated in this article, or claim that may be made by its manufacturer, is not guaranteed or endorsed by the publisher.
