# Corrigendum: Human mental workload: A survey and a novel inclusive definition

**DOI:** 10.3389/fpsyg.2022.969140

**Published:** 2022-07-26

**Authors:** Luca Longo, Christopher D. Wickens, Gabriella Hancock, P. A. Hancock

**Affiliations:** ^1^Artificial Intelligence and Cognitive Load Lab, The Applied Intelligence Research Centre, School of Computer Science, Technological University Dublin, Dublin, Ireland; ^2^Department of Psychology, Colorado State University, Fort Collins, CO, United States; ^3^Department of Psychology, California State University, Long Beach, CA, United States; ^4^Department of Psychology, Institute for Simulation and Training, University of Central Florida, Orlando, FL, United States

**Keywords:** survey, mental workload, definitions, theories, measures, models, novel framework, novel inclusive definition

In the published article, the name of Gabriella Hancock was incorrectly written as “Gabriela M. Hancock.” The correct name is “Gabriella Hancock.”

In the published article, there was also an error in the author list as published. Gabriella Hancock was listed as the last author, but should have been listed as third author. P. A. Hancock was listed as third author but should be listed as the last author. The corrected author list appears below.

Luca Longo^1*^, Christopher D. Wickens^2^, Gabriella Hancock^3^ and P. A. Hancock^4^

The authors apologize for this error and state that this does not change the scientific conclusions of the article in any way. The original article has been updated.

## Publisher's note

All claims expressed in this article are solely those of the authors and do not necessarily represent those of their affiliated organizations, or those of the publisher, the editors and the reviewers. Any product that may be evaluated in this article, or claim that may be made by its manufacturer, is not guaranteed or endorsed by the publisher.

