# Supplementary material for: Human Mental Workload: A Survey and a Novel Inclusive Definition
Source: Front Psychol. 2022 Jun 2;13:883321. doi: 10.3389/fpsyg.2022.883321 (PMC9201728; doi:10.3389/fpsyg.2022.883321)
Supplement: Supplementary file 1 [file Data_Sheet_1.pdf]

Supplementary Material

Order	Theory	Articles referencing it	Original work
1	Activity theory	(Salmon et al., 2006; Jones, 2009)	(Vygotsky, 1980)
2	Arousal theory	(Staal, 2004)	(Cohen, 2011)
3	Cognitive appraisal theory	(Besson et al., 2013)	(Zajonc, 1984)
4	Cognitive load theory	(Albers, 2011; Byrne, 2011; Dirican and Göktürk, 2011; Galy et al., 2012; Gwizdka, 2010; Paas et al., 2003; Schmutz et al., 2009, 2010) (Tracy and Albers, 2006; Wiebe et al., 2010; Whelan, 2007; Arguel and Jamet, 2009; Antonenko et al., 2010; Byrne et al., 2014; Neßelrath, 2013; Walter et al., 2013) (Camp et al., 2001; Gavas et al., 2017; Tavares and Eva, 2013)	(Sweller, 2011)
5	Contextual Action Theory	(Young and Stanton, 1997)	(Stanton, 1995)
6	Distributed cognition	(Saleem et al., 2009)	(Hollan et al., 2000)
7	Event perception theory	(Adamczyk et al., 2005; Bailey and Konstan, 2006)	(Johansson et al., 1980)
8	Eysenck's theory	(Rose et al., 2002)	(Gray, 1981)
9	Flow theory	(Keller et al., 2011)	(Csikszentmihalyi, 2000)
10	Game theory	(Li et al., 2011)	(Bakr et al., 2008)
11	Herzberg's two-factor theory	(Simon, 1978)	(Herzberg, 1966)
12	Information processing theory	(Athènes et al., 2002)	(Simon, 1978)
13	Job enrichment theory	(Cook and Salvendy, 1999)	(Hackman and Oldham, 1976)
14	Malleable attentional resources theory	(Basahel et al., 2010)	(Young and Stanton, 2002a)
15	Motivational intensity theory	(Venables and Fairclough, 2009)	(Richter et al., 2016)
16	Multiple resource theory	(Gwizdka, 2010; Longo, 2015; MacDonald, 1999; Mitchell, 2000; Prewett et al., 2010; Schulte et al., 2015; Verwey, 1990)<br>(Wickens, 2008; Wu et al., 2008; Wickens, 2002; Stanton et al., 2004; Bommer, 2013; Morrison, 1993)<br>(Moustafa et al., 2017; Lukanov et al., 2016; Mehler et al., 2012; Brown, 1997; Caggiano and Parasuraman, 2004; Colombi et al., 2012; Schneider et al., 2011; Usui and Egawa, 2002)<br>(Zheng et al., 2010; Iwata et al., 2010; Wu et al., 2008; Rusnock and Geiger, 2017; Brand and Schulte, 2017; Britt et al., 2015; Young and Stanton, 2002b)	(Wickens, 2002)
17	Processing efficiency theory	(Mandrick et al., 2016)	224 (Eysenck and Calvo, 1992)
18	Theory of skilled behaviour	(Morris et al., 2007)	(Rasmussen, 1983)
19	Relevance theory	(Ismail and Grivard, 2015)	(Smolka and Pirker, 2018)
20	Cognitive theory of ageing	(Reimer et al., 2008)	(Salthouse, 2000)
21	Schemata theory	(Byrne, 2013)	(Oldfield, 1954)
22	Self-awareness theory	(Ma and Kaber, 2005)	(Oldfield, 1954)

Table S1: Different theories and the number of times they were discussed in the sample considered for the current literature review.

Table S2: Definitions of mental workload found in the literature

#	Definition
1	Mental load is the aspect of cognitive load that originates from the interaction between task and subject characteristics (Paas et al., 2003)
2	Cognitive load is defined as a multidimensional construct representing the load that a particular task imposes on the performer (Haapalainen et al., 2010)
3	Mental workload expresses the task demands placed on an operator (Colombi et al., 2012)
4	Mental workload is recognized as a multi-dimensional concept that is largely driven by the characteristics of local task demands (Hancock and Caird, 1993)
5	MWL can be seen as an interaction between the demands of the task and the performance of the operator (Byrne, 2011)
6	The cost incurred by a human operator to achieve a particular level of performance (Marquart and de Winter, 2015)
7	The concept of workload is fundamentally defined by the relationship between resource supply and task demand (Haga et al., 2002)
8	Mental workload is defined as the demanded resources of human information processing for performing a task (Liang et al., 2014)
9	Depletion of human internal resources to accomplish the presented work (Leung et al., 2010).
10	Mental workload is the relationship between primary task performance and the resources demanded by the primary task (Lukanov et al., 2016)
11	Mental workload is a related construct that refers to the amount of resources consumed by a task (Mizobuchi et al., 2005)
12	Cognitive load (also referred to as mental workload) is commonly defined as the relationship between the cognitive demands placed on a user by a task and the user's cognitive resources (Palinko et al., 2010)
13	The cost of performing a task in terms of a reduction in the capacity to perform additional tasks that use the same processing resource (Cain, 2007)
14	Workload can be defined as how the operators can do the required work (their capacities) and how they can manage the task (task demands) to satisfy the operating system demand (Basahel et al., 2010)
15	The relative capacity to respond, the emphasis is on predicting what the operator will be able to accomplish in the future (Cain, 2007)
16	MWL has been defined as the amount of cognitive capacity required to perform a given task (Di Stasi et al., 2013a)
17	Mental workload may be described as the use and temporary expenditure of a finite amount of information processing capacity (Wästlund, 2007)
18	Mental workload is usually associated with information processing tasks, but any human activity includes mental processing and thus, mental workload (Mitchell, 2000)
19	...a measurable quantity of the information processing demands placed on an individual by a task (Annett, 2002)
20	The concept of mental workload is often used to describe how much of someone's information-processing capacity is needed during task performance and how this is influenced by task demands (Stuiver et al., 2014)
21	Mental workload refers to the ability of the operator to meet the information processing demands imposed by a task or system (Wilson and Eggemeier, 2006)
22	Mental workload is used to describe the amount of mental effort involved in performing any given task (Byrne et al., 2010)
23	Mental workload refers to the cognitive effort expended during a particular task (Tavares and Eva, 2013)
24	Mental workload is defined as the overall cognitive effort a person invests in his performance while carrying out a task (Baldauf et al., 2009)
25	The mental effort that the human operator devotes to control or supervision relative to his capacity to expend mental effort (Cain, 2007)
26	Mental workload is described as a noticeable relationship between the human cognitive capacity and an effort required to process a particular function (Hou et al., 2015).
27	The intensity of mental effort can be considered as an index of mental workload. It may be defined as the total amount of controlled cognitive processing in which a subject is engaged (Paas and Van Merriënboer, 1993)
28	Mental workload or cognitive load refers to the total amount of human mental effort or memory that is required for the execution of a task (Chen et al., 2016)
29	Cognitive load or the mental workload is characterized by the amount of memory resources utilized to ascertain a task (Gavas et al., 2017)
30	Mental workload refers to the amount of information and/or the complexity of mental operations that are held in or processed by working memory which depends on the prefrontal cortex (Stock et al., 2016)
31	Mental workload can be described by the demand placed on user's working memory during a task (Fréard et al., 2007)
32	Workload is a construct used to describe the extent to which an operator has engaged the cognitive and physical resources required for task performance (Weinger et al., 2004)
33	Mental workload is commonly defined as the extent to which human mental resource is able to meet the cognitive demands of the task (Lim et al., 2015)
34	Mental workload is a hypothetical construct describing the extent to which the cognitive resources required to perform a task that has been actively engaged by the operator (Miller, 2001)
35	Mental workload describes the level of mental resources utilized when a person is performing a task (Wang et al., 2016)
36	Workload is commonly defined as the degree of processing capacity that is expended during task performance, and it reflects a relationship between resource supply and task demand (Young et al., 2008)
37	Mental workload has been generally defined as the amount of resource difference between task demands and capacity of an individual (Lin et al., 2011)

38	MWL is defined as the level of processing capacity while performing the task or the difference between the capacity to affect the usable real performance and human-information processing system (Kum et al., 2007)
39	Mental workload is related to the difference between the amount of resources available within a person and the amount of resources demanded by the task situation (Young and Stanton, 1997)
40	Mental workload can be defined as the difference between the amount of available mental processing resources and the amount required by a task (Harriott et al., 2015)
41	Mental workload is the difference between the capacities of the information processing system that are required for task performance to satisfy performance expectations and the capacity available at any given time (Omolayo and Omole, 2013)
42	Mental workload is commonly defined as the proportion of a person's total mental capacity in use at a given moment (Pierce, 2009)
43	The term workload refers to that portion of the operator's limited capacity actually required to perform a particular task (Kim et al., 2014; Alexander et al., 2000)
44	Mental workload refers to the portion of operator information processing capacity or resources that is actually required to meet system demands (Cain, 2007; Borghini et al., 2014)
45	Mental workload is usually defined as the ratio between task demands and a person's capacity (Brouwer et al., 2012)
46	Mental workload is defined more generally as the ratio of the resources required to complete a series of tasks to resources available to complete as series of tasks (Lodree Jr et al., 2009)
47	The ratio between processing power and data coming from the environment determines mental workload (Frey et al., 2013)
48	Workload is the extent to which an operator is occupied by a task (Verwey, 1990)
49	The mental workload of any given task is the ratio of mental resources required to the total resources available (Hu et al., 2016)
50	The operator's evaluation of the attentional load margin (Haga et al., 2002)
51	Mental workload is related to the difference between the amount of finite resources (attention or mental effort) available within a person and the amount of resources demanded by the tasks being performed (Saleem et al., 2009)
52	Intuitively, it can be described as the amount of cognitive work expended to a certain task during a given period of time (Rizzo et al., 2016)
53	MWL can be defined as the amount of cognitive work required for a person to complete a certain task over time (Longo, 2016; Longo and Dondio, 2015)
54	It can be intuitively described as the total cognitive load needed to accomplish a specific task under a finite period of time (Moustafa et al., 2017)
55	Workload is the amount of work that expected to be done by an operator in a specified time. In another words, it is the interaction between the operator and assigned task (Kum et al., 2008a)
56	Mental workload can be seen as the % of mental capacity in use at any time-point (Byrne et al., 2014)
57	Mental workload for a given task is the ratio of mental resources required to the total resources available, on a moment-to-moment basis (Carswell et al., 2005)
58	Workload can be defined as the ratio of the time required to perform the tasks to the time available (Wickens, 2002)
59	Mental workload would seem to be some combination of mental effort, information processing and emotion in response to task demand (Sheridan and Simpson, 1979)
60	The term workload will be used to refer to the integrated effects on the human operator of task-related, situation-related, and operator-related factors that occur during the performance of a task (Verwey, 1990)
61	Mental workload is an inferred construct that mediates between task difficulty, operator skill, and observed performance (Staal, 2004)
62	Mental load as a concept now serves as an intermediary between imposed and perceived demands (Young and Stanton, 1997)
63	Put most simply, mental workload is the amount of mental work or effort necessary for a person or a group to complete a task over a given period of time (Xie and Salvendy, 2000a)
64	Mental workload associated with a task has been described as relating to the rate at which information is processed by an operator, the rate at which decisions are made, and the difficulty of making the decisions (Smiley, 1989)
65	Mental workload is related to the amount of attention required for making decisions (Miller, 2001)
66	the level of attentional resources required to meet both objective and subjective performance criteria, which may be mediated by task demands, external support, and past experience (Young et al., 2015; Young and Stanton, 2001)
67	Mental workload can be defined as the amount of thinking, level of cognitive demand, or thought processing effort required by the worker to meet the physical, temporal, and environmental demands of the defined task (Neill, 2011)
68	When we speak of mental workload, we are referring to some sense of mental effort, The basic idea is that we have a finite capacity for performing mental work; and if we exceed this capacity, then we will begin to make a large number of errors or experience total performance breakdown (Potter and Bressler, 1989)

Table S3: Self-report measures of mental workload and the reported articles that used them

Subjective measure	Studies that used these measures
Stress survey	(Carter et al., 2005)
Rating Scale Mental Effort	(Olsson and Burns, 2000; Brookhuis et al., 2009; Johnson and Widyanti, 2011; Tørnros and Bolling, 2005; Veltman and Gaillard, 1998; Lin et al., 2003)
Usefulness score	(Brookhuis et al., 2009)
NASA-TLX	(Athènes et al., 2002; Banerjee et al., 2011; Britt et al., 2015; Brouwer et al., 2012; Byrne et al., 2010; Carswell et al., 2005; Chaouachi et al., 2011; Cinaz et al., 2013; Matthews et al., 2015; Cook and Salvendy, 1999; Darvishi et al., 2016; Gómez-Gómez et al., 2015; Haga et al., 2002) (Hancock, 1988, 1989; Hancock and Caird, 1993; Hancock et al., 1995; Harris et al., 1995; Hoover et al., 2012; Hwang et al., 2007; Jahn et al., 2005; Johnson and Widyanti, 2011; Jones, 2009) (Jou et al., 2009; Kataoka et al., 2011; Kawakita et al., 2010; Kiselev and Loutfi, 2012; Kjeldskov and Stage, 2004; Kokini et al., 2012; Longo and Dondio, 2015; Mayes et al., 2001; Mayser et al., 2003; Mitchell, 2000) (Miyake, 2001; Miyake et al., 2009; Moroney et al., 1992; Noyes and Bruneau, 2007; Tremoulet et al., 2009; Trujillo, 1998; Vera et al., 2017; Vitório et al., 2012; Wiebe et al., 2010; Wu and Liu, 2007) (Piechulla et al., 2003; Riccio et al., 2011; Safari et al., 2013; Schmutz et al., 2009, 2010; Shinohara et al., 2002; Singh et al., 2009, 2010; Stefanidis et al., 2007; Svensson et al., 1997) (Wu et al., 2008; Xie and Salvendy, 2000b; Jou et al., 2009; Harbluk et al., 2007; Ikuma et al., 2014; Knaepen et al., 2015; Liang et al., 2014; Huber et al., 2006; Chen et al., 2011a; Kang et al., 2004) (Bommer, 2013; Tokunaga et al., 2001; Marquart and de Winter, 2015; Moustafa et al., 2017; Barnard et al., 2007; Haapalainen et al., 2010; Dey and Mann, 2010; Colle and Reid, 1998; Fréard et al., 2007; Moustafa and Longo, 2018; Harriott et al., 2015) (Lukanov et al., 2016,?; Tungare and Pérez-Quiñones, 2009; Mark et al., 2008; Schneegass et al., 2013; Basahel et al., 2010; Adamczyk and Bailey, 2004; Nielsen et al., 2006; Haapalainen et al., 2010; Fritz et al., 2014) (Fairclough et al., 2005; Newell and Mansfield, 2008; Arguel and Jamet, 2009; Wang and Dunston, 2006; France et al., 2005; Colligan et al., 2015; Rani et al., 2007; Baulk et al., 2007; Bradley and Dunlop, 2005; Byrne et al., 2014; Caggiano and Parasuraman, 2004) (Cinaz et al., 2013; Engelmann et al., 2011; Epling et al., 2016; Guznov et al., 2011; Helton et al., 2005; Hu et al., 2016; Hubert et al., 2013; Fisher and Ford, 1998; Kajiwara, 2014; Kataoka et al., 2011) (Liang et al., 2009; Lin et al., 2011, 1998; Luz et al., 2014; Rebetez et al., 2010; Rose et al., 2002; Rubio et al., 2004; Ruiz-Rabelo et al., 2015; Shinohara et al., 2002; Di Stasi et al., 2013b; Venables and Fairclough, 2009; Zheng et al., 2010, 2012; Putze et al., 2010; Lin and Wu, 2011; Zhang et al., 2014; Makhtar et al., 2011; Iwata et al., 2010; Besson et al., 2012a) (Besson et al., 2013; Gentili et al., 2014; Borghini et al., 2015; Zhang et al., 2015a; Liang et al., 2018; Entin et al., 1998; Wu et al., 2008; Won et al., 2011; Yanghua and Fansen, 2011; Besson et al., 2012b; Bodala et al., 2014) (Durkee et al., 2015; Rusnock and Geiger, 2017; Krausman, 2017; Villa and Halvey, 2013; Lan et al., 2010; Young et al., 2009; Leung et al., 2010; Stefanidis et al., 2010)
SWAT	(Baldauf et al., 2009; Carayon and Gürses, 2005; Carswell et al., 2005; Colle and Reid, 2005; Hancock et al., 1995; Luximon and Goonetilleke, 1998, 2001; Mitchell, 2000) (Pickup et al., 2005; Wittmann et al., 2006; Ikuma et al., 2014; Roscoe and Ellis, 1990; Rubio et al., 2004; Zhang et al., 2015a; Dey and Mann, 2010; Colle and Reid, 1998)
Modified RTLX for automobile apps.	(Piechulla et al., 2003)
Subjective Reports of Effort and Perceived Task Difficulty	(Pierce, 2009)
Karolinska Sleepiness Scale (KSS)	(Roy et al., 2016; Elmenhorst et al., 2009)
Standard Sleepiness Scale	(Vera et al., 2017)
Workload Profile	(Moustafa et al., 2017; Rubio et al., 2004; Fréard et al., 2007; Moustafa and Longo, 2018; Longo and Dondio, 2015; Tsang and Velazquez, 1996)
Scale for subjective rating of task difficulty (SRTD)	(KAKIZAKI, 1987)
Crew Awareness rating Scale	(Bommer, 2013)
Job content questionnaire (JCQ)	(Collins et al., 2005)
Situation Awareness Global Assessment Technique (SAGAT)	(Saleem et al., 2009; Ikuma et al., 2014; Luz et al., 2014)
Modified Cooper-Harper scale	(Wierwille et al., 1985)
DSTAI (STAI variation)	(Brayda et al., 2015)
Situational Awareness	(Alexander et al., 2000; Saleem et al., 2009; Trujillo, 1998; Salmon et al., 2006)
Standard Sleepiness Scale	(Vera et al., 2017)

Scale for subjective rating of task difficulty (SRTD)	(KAKIZAKI, 1987)
Crew Awareness rating Scale	(Bommer, 2013)
Nine point symmetrical category scale	(Paas and Van Merriënboer, 1993)
Taylor Manifest Anxiety symptoms test	(Guastello et al., 2012)

Table S4: Physiological and neurophysiological measures of mental workload and the articles that used them

Physiological measure	Studies that used these measures
Muscular Sympathetic Nerve Activity	(Carter et al., 2005)
Electroencephalography	(Arico et al., 2015; Aricò et al., 2016; Berka et al., 2007; Brookings et al., 1996; Carswell et al., 2005; Cartocci et al., 2015; Chaouachi et al., 2011; Matthews et al., 2015; Fritz et al., 2014; Haapalainen et al., 2010; Dirican and Göktürk, 2011; Haga et al., 2002; Hou et al., 2015; Aghajani et al., 2017; Dussault et al., 2005) (Lim et al., 2015; Ling et al., 2001; Mak et al., 2013; Mazaeva et al., 2001; Mitchell, 2000; Riccio et al., 2011; Roy et al., 2015, 2016; Ryu and Myung, 2005; Tremoulet et al., 2009; Krol et al., 2016) (Wanyan et al., 2014; Wilson and Russell, 2003a; Yin and Zhang, 2017; Zhang et al., 2015b; Zhou et al., 2008; KAKIZAKI, 1987; Kim et al., 2014; Yin and Zhang, 2014; Kang et al., 2004; Liu et al., 2017; Zhang et al., 2017a; Plechawska-Wójcik and Borys, 2016) (Chen and Vertegaal, 2004; Hirshfield et al., 2009; Montgomery et al., 1995; Lan et al., 2010; Fairclough et al., 2005; De Bruin et al., 2002; Sammer et al., 2007; Venables and Fairclough, 2009; Marshall, 2002; Putze et al., 2010; Mathan et al., 2010) (Besson et al., 2012a; Hwang et al., 2014a; Zhang et al., 2014; Zarjam et al., 2015; Wang et al., 2016; Rozado and Dunser, 2015; Blanco et al., 2018; Chang et al., 2016; Mallick et al., 2016; Magnusdottir et al., 2017; Almogbel et al., 2018; Kim et al., 2014) (Hwang et al., 2014b; Gentili et al., 2014; Borghini et al., 2015; Putze et al., 2015; Ke et al., 2014, 2015; Lim et al., 2016; Kraft et al., 2017; Durkee et al., 2015; Oyama et al., 2013; Walter et al., 2013; Bodala et al., 2014) (Kothe and Makeig, 2011; Kramer et al., 1987; Laine et al., 2002; Zhang et al., 2017b; Haapalainen et al., 2010; Herff et al., 2015; Bodala et al., 2015; Klosterman et al., 2016; Dimitrakopoulos et al., 2017; Liang et al., 2018; Ling et al., 2001; Wang et al., 2011) Hernández-Sabaté et al. (2022); Kutafina et al. (2021); Pei et al. (2020); Diaz-Piedra et al. (2020); Kakkos et al. (2021); Guan et al. (2022); Raufi and Longo (2022)
Magnetic Resonance Imaging	(Ryu and Myung, 2005; Wilson and Russell, 2003a)
Pupillary responses	(Tungare and Pérez-Quiñones, 2009; Haapalainen et al., 2010; Elkins and Hossain, 2015)
Pupil size	(Cegarra and Chevalier, 2007; Dirican and Göktürk, 2011; de Greef et al., 2009; He et al., 2012; Iqbal et al., 2004; Kawakita et al., 2010; Schultheis and Jameson, 2004; Di Stasi et al., 2013a; Wierwille et al., 1985; Zhang et al., 2004; Marquart and de Winter, 2015; Bailey and Iqbal, 2008) (Palinko et al., 2010; Iqbal et al., 2005; Chen et al., 2011b; Xu et al., 2011; Lin et al., 2003; Marshall, 2002; Mallick et al., 2016; Wang et al., 2014; Plechawska-Wójcik and Borys, 2016; Gavas et al., 2017)
Eye movements	(Dirican and Göktürk, 2011; Di Stasi et al., 2013a; Svensson et al., 1997; Kataoka et al., 2011; Marshall, 2002; Bodala et al., 2015; Bedziouk et al., 2006)
Blink rate	(Brookings et al., 1996; Carswell et al., 2005; Ryu and Myung, 2005; Davis, 1994; Marquart and de Winter, 2015; Elmenhorst et al., 2009; Chen et al., 2011b)
Blink closure duration	(Ryu and Myung, 2005)
Blink duration	(Hwang et al., 2007)
Interocular pressure	(Vera et al., 2017)
Respiration rate	(Brookings et al., 1996; Cegarra and Chevalier, 2007; Dirican and Göktürk, 2011; Haga et al., 2002; Veltman and Gaillard, 1998; Wierwille et al., 1985; Wilson and Russell, 2003a; Lin et al., 2007; Zheng et al., 2012; Putze et al., 2010; Besson et al., 2012a, 2013, 2012b; Mehler et al., 2009)
Respiratory volume	(Ohsuga et al., 2001)
Electrocardiography	(Brookhuis et al., 2009; Cinaz et al., 2010; Mahmoud et al., 2017; Matthews et al., 2015; Kumar et al., 2007; Piechulla et al., 2003; Wanyan et al., 2014; Wilson and Russell, 2003a; Zhang et al., 2015b; Liu et al., 2017; Montgomery et al., 1995; Lan et al., 2010; Fairclough et al., 2005; Heine et al., 2017) (Cinaz et al., 2013; Collins et al., 2005; Engelmann et al., 2011; Hjortskov et al., 2004; Sammer et al., 2007; Venables and Fairclough, 2009; Zhang et al., 2014; Chang et al., 2016; Besson et al., 2013; Gentili et al., 2014; Itoh, 2009; Besson et al., 2012b; Durkee et al., 2015; Zhang et al., 2017a) (Stuiver et al., 2014; Boucsein and Thum, 1997; Schellekens et al., 2000; Kraft et al., 2017; Haapalainen et al., 2010)
Pulse rate	(Liang et al., 2009)

Heart rate	(Cárdenas-Vélez et al., 2013; Davis et al., 2009; Galy et al., 2012; Murai et al., 2004; Pierce, 2009; Svensson et al., 1997; Twisk et al., 2013; Wanyan et al., 2014; Son et al., 2011; Elmenhorst et al., 2009; Ward and Marsden, 2003; Kataoka et al., 2011; Nickel and Nachreiner, 2003) (Luz et al., 2014; Son et al., 2013; Usui and Egawa, 2002; Zheng et al., 2012; Besson et al., 2012a; Reimer et al., 2008; Mehler et al., 2009)
Heart rate variability	(Brookhuis et al., 2009; Brookings et al., 1996; Hoover et al., 2012; Hwang et al., 2007; Jahn et al., 2005; Keller et al., 2011; Kum et al., 2007; Kumar et al., 2007; Mehler et al., 2011; Miyake, 2001; Murai and Hayashi, 2008,?) (Ryu and Myung, 2005; Vera et al., 2017; Davis, 1994; Knaepen et al., 2015; Green, 1994; Schneegass et al., 2013; Basahel et al., 2010; Rowe et al., 1998; Haapalainen et al., 2010)
Oxygen consumption	(Cárdenas-Vélez et al., 2013)
Salivary cortisol levels	(Keller et al., 2011; Hankins and Wilson, 1998)
Salivary amylase activity value	(Hama et al., 2009)
Saccade rate	(Brookings et al., 1996; Pierce, 2009)
Saccade distance	(de Greef et al., 2009)
Saccade speed	(de Greef et al., 2009; Chen et al., 2011b)
Head and body movements	(Twisk et al., 2013)
Fixation time	(de Greef et al., 2009; He et al., 2012; Chen et al., 2011b; Di Nocera et al., 2006)
Electrooculography	(Haga et al., 2002; Kothe and Makeig, 2011; Kramer et al., 1987; Roy et al., 2015, 2016; Ryu and Myung, 2005; Wilson and Russell, 2003a; Zhang et al., 2015b; Yin and Zhang, 2014; Elmenhorst et al., 2009; Chen and Vertegaal, 2004; Fairclough et al., 2005) (Mahmoud et al., 2017; Klosterman et al., 2016; Zhang et al., 2017a)
Core temperature variation	(Hancock, 1988)
Blood rate	(Hwang et al., 2007)
Blood volume	(Ryu and Myung, 2005; Ward and Marsden, 2003)
Blood pressure	(Ohsuga et al., 2001; Veltman and Gaillard, 1998; Elmenhorst et al., 2009; Basahel et al., 2010; Luz et al., 2014; Van Roon et al., 2004; Usui and Egawa, 2002)
Blood flow	(Wilson and Russell, 2003a)
Para/Sympathetic ratio	(Hwang et al., 2007)
Gaze angle	(Kawakita et al., 2010)
Head rotation angle	(Kawakita et al., 2010)
Finger plethysmogram amplitude	(Miyake, 2001)
Perspiration	(Miyake, 2001)
Nasal-Forehead temperature	(Murai and Hayashi, 2008)
Skin temperature	(Ohsuga et al., 2001; Trujillo, 1998; Knaepen et al., 2015; Schneegass et al., 2013)
Skin conductance	(Pierce, 2009; Knaepen et al., 2015; Son et al., 2011; Schneegass et al., 2013; Kajiwara, 2014; Venables and Fairclough, 2009; Zheng et al., 2012; Putze et al., 2010; Besson et al., 2012a, 2013, 2012b; Mehler et al., 2009)
Skin response	(Nourbakhsh et al., 2012; Zhang et al., 2014)
Electromyography	(Piechulla et al., 2003; Tanaka et al., 2000; Wilson and Russell, 2003a; Boucsein and Thum, 1997; Hubert et al., 2013; Zarjam et al., 2015; Rozado and Dunser, 2015; Besson et al., 2013, 2012b; Zhang et al., 2016)
Electrodermal activity	(Baldauf et al., 2009; Tanaka et al., 2000; Wilson and Russell, 2003a; Fritz et al., 2014; Boucsein and Thum, 1997; Rani et al., 2006; Chang et al., 2016)
Actigraphy	(Wilson and Russell, 2003a)
Oxymetry	(Wilson and Russell, 2003a)
Body temperature	(Lin et al., 2007)
Functional near-infrared spectroscopy	(Unni et al., 2015; Ung et al., 2017; Herff et al., 2015; Berivanlou et al., 2016) Durantin et al. (2014); Sibi et al. (2016); Karim et al. (2012); Sassaroli et al. (2008); Li et al. (2019); Parshi et al. (2019); Galoyan et al. (2021)
Purdue Pegboard test	(Zhang et al., 2016)
Urinary adrenaline	(Hankins and Wilson, 1998)

Table S5: Criteria to assess mental workload measures and the articles that used them

Criteria	Used	Discussed
Reliability	(Paas and Van Merriënboer, 1993; Tsang and Velazquez, 1996; Yeung et al., 2000; Lin et al., 2011; Zhang et al., 2015a; Zarjam et al., 2015; Bertram et al., 1990) (Bertram et al., 1992; Lin and Hwang, 1998; Fisher and Ford, 1998; Martens and Van Winsum, 2000; Matthews et al., 2015; Lin et al., 2003; Byrne et al., 2014) (Piechulla et al., 2003; Wastlund et al., 2005; Kum et al., 2008b; Lan et al., 2010; Weigl et al., 2014; Azadeh et al., 2013; Mangen et al., 2013)	(Roscoe and Ellis, 1990; Stanton et al., 2004; Pretorius and Cilliers, 2007; Singh et al., 2010; Zhao et al., 2016; Moustafa et al., 2017) (Shingledecker, 1983; Lysaght et al., 1989; Rowe et al., 1998; Parasuraman et al., 2000; Paas et al., 2003; Longo, 2016) (Carswell et al., 2005; Hart, 2006; Salmon et al., 2006; Young et al., 2008; Gørges and Staggers, 2008; Wiebe et al., 2010) (Antonenko et al., 2010; Tavares and Eva, 2013; Dey and Mann, 2010; Frey et al., 2013; Borghini et al., 2014; Longo and Dondio, 2015; Hoc, 2001)
Validity	(Tsang and Velazquez, 1996; Yeung et al., 2000; Lin et al., 2011; Bertram et al., 1990, 1992; Lin and Hwang, 1998; Moustafa and Longo, 2018) (Rubio et al., 2004,?; Stefanidis et al., 2007; Schneider et al., 2011; Azadeh et al., 2013; Liang et al., 2014; Matthews et al., 2015)	(Stanton et al., 2004; Pretorius and Cilliers, 2007; Rizzo et al., 2016; Moustafa et al., 2017; Shingledecker, 1983; Lysaght et al., 1989) (Morrison, 1993; Boucsein and Thum, 1997; Rowe et al., 1998; Colle and Reid, 1998; Schellekens et al., 2000; Annett, 2002; Dekker and Hollnagel, 2004; Carswell et al., 2005) (Pickup et al., 2005; Zhang and Luximon, 2005; Segall et al., 2005; Hart, 2006; Salmon et al., 2006; Newell and Mansfield, 2008) (Gørges and Staggers, 2008; Wiebe et al., 2010; Tavares and Eva, 2013; Dey and Mann, 2010; Wilson et al., 2011; Fritz et al., 2014) (de Winter, 2014; Nakagawa et al., 2014; Marquart and de Winter, 2015; Longo, 2016)
Sensitivity	(Tsang and Velazquez, 1996; Lin et al., 2011; Wierwille et al., 1985; Verwey and Veltman, 1996; Luximon and Goonetilleke, 2001; Rubio et al., 2004) (Rubio et al., 2004; Fréard et al., 2007; Wiebe et al., 2010; Dey and Mann, 2010; Azadeh et al., 2013; Matthews et al., 2015) (Knaepen et al., 2015; Zarjam et al., 2015; Tsang and Velazquez, 1996; Lin et al., 2011; Wierwille et al., 1985; Verwey and Veltman, 1996) (Luximon and Goonetilleke, 2001; Rubio et al., 2004,?; Fréard et al., 2007; Wiebe et al., 2010; Dey and Mann, 2010) (Azadeh et al., 2013; Matthews et al., 2015; Knaepen et al., 2015; Zarjam et al., 2015)	(Hart and Staveland, 1988; Shinohara et al., 2002; Ryu and Myung, 2005; Pretorius and Cilliers, 2007; Whelan, 2007; Stuiver et al., 2014) (Rizzo et al., 2016; Moustafa et al., 2017; Hoc, 2001; Shingledecker, 1983; Lysaght et al., 1989; Morrison, 1993) (Rowe et al., 1998; Paas et al., 2003; Staal, 2004; Caggiano and Parasuraman, 2004; Carswell et al., 2005; Pickup et al., 2005) (Zhang and Luximon, 2005; Fuller, 2005; Fairclough et al., 2005; Hart, 2006,?; Wittmann et al., 2006; Colligan et al., 2015) (Salmon et al., 2006; Jones, 2009; Miyake et al., 2009; Lodree Jr et al., 2009; Mehler et al., 2009, 2011) (Son et al., 2011; Wilson et al., 2011; Bommer, 2013; da Silva, 2014; Borghini et al., 2014; De Winter et al., 2014; Longo, 2016)
Diagnosticity	(Tsang and Velazquez, 1996; Verwey and Veltman, 1996; Rubio et al., 2004; Dey and Mann, 2010; Zhang et al., 2015a)	(Roscoe and Ellis, 1990; Pretorius and Cilliers, 2007; Stuiver et al., 2014; Moustafa et al., 2017; Shingledecker, 1983; Wierwille et al., 1985) (Lysaght et al., 1989; Morrison, 1993; Rowe et al., 1998; Zhang and Luximon, 2005; Fairclough et al., 2005; Mehler et al., 2009) (Wiebe et al., 2010; Wilson et al., 2011; Bommer, 2013; da Silva, 2014; Matthews et al., 2015; Longo, 2016)

Table S6: Triangulation of measures and the articles that used them

Type of measure	Artefacts that used these measures
Physiological measures only	(Albers, 2011; Arico et al., 2015; Aricò et al., 2016; Bailey and Iqbal, 2008; Cárdenas-Vélez et al., 2013; Cartocci et al., 2015; Dirican and Göktürk, 2011; Hancock et al., 1985; He et al., 2012; Hou et al., 2015; Ke et al., 2014; Ling et al., 2001; Mak et al., 2013; May et al., 1990) (Ke et al., 2015; Kothe and Makeig, 2011; Kum et al., 2007; Kumar et al., 2007; Laine et al., 2002; Lim et al., 2015; Mazaeva et al., 2001; Mehler et al., 2011; Miller, 2001; Murai and Hayashi, 2008) (Roy et al., 2015; Murai and Hayashi, 2008; Ryu and Myung, 2005; Schultheis and Jameson, 2004; Smiley, 1989; Di Stasi et al., 2013a; Tanaka et al., 2000; Wanyan et al., 2014; Davis, 1994; Wilson and Russell, 2003a; Yin and Zhang, 2017) (Zhang et al., 2004, 2015b; Zhou et al., 2008; Lin et al., 2007; Hama et al., 2009; Kim et al., 2014; Yin and Zhang, 2014; Son et al., 2011; Liu et al., 2017; Nourbakhsh et al., 2012; Chen and Vertegaal, 2004) (Xu et al., 2011; Hirshfield et al., 2009; Ward and Marsden, 2003; Hwang et al., 2014a; Tokuda and Obinata, 2012; Rozado and Dunser, 2015; Blanco et al., 2018; Chang et al., 2016; Ung et al., 2017; Magnusdottir et al., 2017; Almogbel et al., 2018; Trujillo, 1998) (Dimitrakopoulos et al., 2017; Ling et al., 2001; Itoh, 2009; Wang et al., 2011; Oyama et al., 2013; Herff et al., 2015; Bodala et al., 2015; Zhang et al., 2017a; Gavas et al., 2017; Hankins and Wilson, 1998) (Karatas et al., 2016; Wang et al., 2014; Klosterman et al., 2016) Kakkos et al. (2019); Foy and Chapman (2018); Fan et al. (2020); Li et al. (2019); Delliaux et al. (2019); Chakladar et al. (2020); Rosanne et al. (2021); Kakkos et al. (2019); Yang et al. (2020); Islam et al. (2020); Ho et al. (2019); Morales et al. (2019); Van Acker et al. (2020); Putze et al. (2019); Di Flumeri et al. (2019) Durantin et al. (2014); Sibi et al. (2016); Karim et al. (2012); Sassaroli et al. (2008); Li et al. (2019); Parshi et al. (2019)
Self-report measures only	(Alexander et al., 2000; Athènes et al., 2002; Ayaz et al., 2012; Bertram et al., 1990, 1992; Brouwer et al., 2012; Cha and Park, 2001; Czaja and Sharit, 1998; Darvishi et al., 2016; Gómez-Gómez et al., 2015; Harris et al., 1995; Kiselev and Loutfi, 2012; Kokini et al., 2012; Chen, 1996) (Longo and Dondio, 2015; Luximon and Goonetilleke, 1998; Mangen et al., 2013; Marcora et al., 2009; Mayser et al., 2003; Moroney et al., 1992; Noyes and Bruneau, 2007; Omolayo and Omole, 2013; Pickup et al., 2005; Rubio et al., 2004; Safari et al., 2013) (Singh et al., 2010; Schmutz et al., 2010; Sheridan and Simpson, 1979; Bellotti et al., 2005; Bradley and Dunlop, 2005; Kuijer et al., 2005; Ruiz-Rabelo et al., 2015; Di Stasi et al., 2013b; Wilson et al., 2011; Tarola et al., 2018; Yanghua and Fansen, 2011) (Chin et al., 2004; Tsang and Velazquez, 1996; Vidulich and Wickens, 1986; Vitório et al., 2012; Wiebe et al., 2010; Wu et al., 2008; Liang et al., 2014; Chen et al., 2011a; Morrison, 1993; Lukanov et al., 2016; Colligan et al., 2015) (Rusnock and Geiger, 2017; Fréard et al., 2007; Moustafa and Longo, 2018) Shan et al. (2021); Pourteimour et al. (2021); Zhang et al. (2020); Mansikka et al. (2019); Qiao et al. (2021); Jansen et al. (2020); Abe et al. (2019); Galy et al. (2018)
Task performance measures only	(Alexander et al., 2000; Moustafa and Longo, 2018; Ayaz et al., 2012; Bertram et al., 1990, 1992; Brouwer et al., 2012; Cha and Park, 2001; Czaja and Sharit, 1998; Darvishi et al., 2016; Gómez-Gómez et al., 2015; Harris et al., 1995; Kiselev and Loutfi, 2012; Kokini et al., 2012) (Longo and Dondio, 2015; Luximon and Goonetilleke, 1998; Mandrick et al., 2016; Mangen et al., 2013; Marcora et al., 2009; Mayser et al., 2003; Moroney et al., 1992; Noyes and Bruneau, 2007; Omolayo and Omole, 2013; Pickup et al., 2005; Rubio et al., 2004)
Physiological measures + Self-report measures	(Carswell et al., 2005; Carter et al., 2005; Cinaz et al., 2013; Hoover et al., 2012; Hwang et al., 2007; Jones, 2009; Kawakita et al., 2010; Keller et al., 2011; Mitchell, 2000; Miyake, 2001; Ohsuga et al., 2001) (Pierce, 2009; Piechulla et al., 2003; Takada and Shimoyama, 2001; Tremoulet et al., 2009; Trujillo, 1998; Wu and Liu, 2007; Knaepen et al., 2015; KAKIZAKI, 1987; Bommer, 2013; Elmenhorst et al., 2009; Basahel et al., 2010) (Rowe et al., 1998; Fritz et al., 2014; Lan et al., 2010; Young et al., 2009; Fairclough et al., 2005; Stuiver et al., 2014; Boucsein and Thum, 1997; De Bruin et al., 2002; Collins et al., 2005; Engelmann et al., 2011; Hubert et al., 2013; Kajiwara, 2014) (Kataoka et al., 2011; Mathan et al., 2010; Besson et al., 2012a; Zhang et al., 2014; Makhtar et al., 2011; Chang et al., 2016; Wang et al., 2014; Putze et al., 2015; Liang et al., 2018; Besson et al., 2012b; Bodala et al., 2014; Brayda et al., 2015; Jimenez-Molina et al., 2018) Wang et al. (2019); Spinelli et al. (2020); Midha et al. (2021); Luong et al. (2020); Bao et al. (2021); Das et al. (2020); Yan et al. (2019); Rad¨untz et al. (2020); Rainieri et al. (2021); Geissler et al. (2021); Marinescu et al. (2018); Jimenez-Molina et al. (2018); Wang et al. (2019) Galoyan et al. (2021)
Physiological measures + Task performance measures	(Berguer and Smith, 2006; Berka et al., 2007; Olsson and Burns, 2000; Cegarra and Chevalier, 2007; Davis et al., 2009; Faure et al., 2016; Galy et al., 2012; Kramer et al., 1987; Tang et al., 2009; Twisk et al., 2013; Vera et al., 2017) (Bailey and Iqbal, 2008; Palinko et al., 2010; Iqbal et al., 2005; Nakagawa et al., 2014; Montgomery et al., 1995; Iani et al., 2004; Koo et al., 2009; Van Roon et al., 2004; Sammer et al., 2007; Son et al., 2013; Lim et al., 2016) (Marshall, 2002; Wang et al., 2016; Unni et al., 2015; Mallick et al., 2016; Hwang et al., 2014b; Elkins and Hossain, 2015; Meier et al., 2016; Itoh, 2009; Walter et al., 2013; Plechawska-Wójcik and Borys, 2016; Krol et al., 2016) (Berivanlou et al., 2016; Kraft et al., 2017; Zhang et al., 2017b; Mehler et al., 2009; Liao et al., 2018)

Task performance measures + Self-report measures	(Bailey and Konstan, 2006; Baldauf et al., 2009; Banerjee et al., 2011; Britt et al., 2015; Byrne et al., 2010; Carayon and Gürses, 2005; Colle and Reid, 2005; Cook and Salvendy, 1999; Dunlop and Davidson, 2000; Guastello et al., 2012; Johnson and Widyanti, 2011; Kjeldskov and Stage, 2004) (Hancock, 1989; Hancock et al., 1995; Kataoka et al., 2011; Liang et al., 2009; Luximon and Goonetilleke, 2001; MacMillan et al., 2004; Mayes et al., 2001; Miyake et al., 2009; Moray et al., 1988; Patten et al., 2004) (Saleem et al., 2009; Schmutz et al., 2009; Shinohara et al., 2002; Singh et al., 2009; Stefanidis et al., 2007; Tørnros and Bolling, 2005; Veltman and Gaillard, 1998; Warm et al., 1991; Wierwille et al., 1985; Xie and Salvendy, 2000b) (Jou et al., 2009; Yeung et al., 2000; Harbluk et al., 2007; Wittmann et al., 2006; Ikuma et al., 2014; Huber et al., 2006; Roscoe and Ellis, 1990; Tokunaga et al., 2001; Moustafa et al., 2017; Barnard et al., 2007; Mark et al., 2008) (Mizobuchi et al., 2005; Adamczyk and Bailey, 2004; Nielsen et al., 2006; Villa and Halvey, 2013; Helton and Warm, 2008; Horrey et al., 2009; Segall et al., 2005; Newell and Mansfield, 2008; Wastlund et al., 2005; Arguel and Jamet, 2009; Wang and Dunston, 2006; France et al., 2005) (Rani et al., 2007; Baulk et al., 2007; Byrne et al., 2014; Caggiano and Parasuraman, 2004; Epling et al., 2016; Guznov et al., 2011; Helton et al., 2005; Hu et al., 2016; Fisher and Ford, 1998; Leung et al., 2010; Lin et al., 2011) (Rubio et al., 2004; Lin et al., 1998; Rebetez et al., 2010; Rose et al., 2002; Sandrock et al., 2009; Shinohara et al., 2002; Iwata et al., 2010; Bertolo et al., 2014; Zhang et al., 2015a; Çapalar and Olaverri-Monreal, 2017; Entin et al., 1998) (Stefanidis et al., 2010; Zheng et al., 2010; Lin and Wu, 2011; Kim et al., 2014; Wu et al., 2008; Meza-Kubo et al., 2009; Won et al., 2011; Krausman, 2017; Dey and Mann, 2010; Colle and Reid, 1998; Paas and Van Merriënboer, 1993; Li et al., 2020)
Subjective measures + Physiological measures + Task performance measures	(Brookhuis et al., 2009; Brookings et al., 1996; Chaouachi et al., 2011; Matthews et al., 2015; de Greef et al., 2009; Haga et al., 2002; Hancock, 1988; Hancock and Caird, 1993; Jahn et al., 2005; Jou et al., 2009; Riccio et al., 2011) (Roy et al., 2016; Svensson et al., 1997; Green, 1994; van Gent et al., 2018; Kang et al., 2004; Marquart and de Winter, 2015; Harriott et al., 2015; Lukanov et al., 2016; Tungare and Pérez-Quiñones, 2009; Schneegass et al., 2013) (Schellekens et al., 2000; Chen et al., 2011b; Haapalainen et al., 2010; Lin et al., 2003; Cinaz et al., 2013; Hjortskov et al., 2004; Liang et al., 2009; Luz et al., 2014; Rani et al., 2006; Usui and Egawa, 2002; Venables and Fairclough, 2009; Zheng et al., 2012) (Putze et al., 2010; Zarjam et al., 2015; Besson et al., 2013; Gentili et al., 2014; Borghini et al., 2015; Reimer et al., 2008; Durkee et al., 2015; Zhang et al., 2016; Haapalainen et al., 2010) Ding et al. (2020); Solís-Marcos and Kircher (2019); Chen et al. (2019); Bommer and Fendley (2018); Orlandi and Brooks (2018)

Table S7: Sensitivity and the ways it was measured by the articles found in the literature

Measure	Example of articles that used these measures
ANOVA	(Tsang and Velazquez, 1996; Wierwille et al., 1985; Rubio et al., 2004,?)
t-test + hierarchical regression analysis	(Lin et al., 2011)
z-transformation + ANOVA	(Verwey and Veltman, 1996)
Factor loading sensitivity analysis	(Luximon and Goonetilleke, 2001)
Discriminant and variance analyses	(Fréard et al., 2007)
Secondary task analysis	(Wiebe et al., 2010)
Factor loading analysis	(Dey and Mann, 2010)
Regression	(Azadeh et al., 2013)
Differences in means	(Matthews et al., 2015)
ANOVA + post-hoc analysis	(Knaepen et al., 2015; Luximon and Goonetilleke, 2001)
Friedman test	(Zarjam et al., 2015)

Table S8: Diagnosticity and the ways it was measured by the articles found in the literature

Measure	Example of articles that used these measures
Canonical discriminant analysis	(Tsang and Velazquez, 1996; Verwey and Veltman, 1996; Rubio et al., 2004)
ANOVA	(Dey and Mann, 2010)
Spearman's correlation	(Zhang et al., 2015a)

Table S9: Reliability and the ways it was measured by the articles found in the literature

Measure	Example of articles that used these measures
Cronbach's alpha)	(Paas and Van Merriënboer, 1993; Lin et al., 2011; Bertram et al., 1990, 1992; Yeung et al., 2000; Kum et al., 2008b; Lan et al., 2010; Azadeh et al., 2013; Byrne et al., 2014; Matthews et al., 2015; Zhang et al., 2015a)
Spearman's correlation test	(Lin and Hwang, 1998)
Kuder–Richardson-20	(Fisher and Ford, 1998)
ANOVA	(Martens and Van Winsum, 2000; Lin et al., 2003; Zarjam et al., 2015)
Intraclass correlation coefficient	(Piechulla et al., 2003)
Interjudge reliability	(Wastlund et al., 2005)
Kappa-coefficient	(Weigl et al., 2014)

Table S10: Validity and the ways it was measured by the articles found in the literature

Measure	Example of articles that used these measures
Cronbach's alpha	(Paas and Van Merriënboer, 1993; Lin et al., 2011; Bertram et al., 1990, 1992; Yeung et al., 2000; Kum et al., 2008b; Lan et al., 2010; Azadeh et al., 2013; Byrne et al., 2014; Matthews et al., 2015; Zhang et al., 2015a; Mangen et al., 2013)
Spearman's correlation test	(Lin and Hwang, 1998)
Kuder–Richardson-20	(Fisher and Ford, 1998)
ANOVA	(Martens and Van Winsum, 2000; Tsang and Velazquez, 1996; Lin et al., 2003; Zarjam et al., 2015)
Intraclass correlation coefficient	(Piechulla et al., 2003)
Interjudge reliability	(Wastlund et al., 2005)
Kappa-coefficient	(Weigl et al., 2014)

Table S11: Approaches and modeling techniques for mental workload applied by the articles found in the literature

Aggregation strategy	Example of articles that used these measures
Average	(Sheridan and Simpson, 1979)
Mathematical modelling and simulation	(Hancock and Chignell, 1988; Chen et al., 2011a)
Weighted average	(Hart and Staveland, 1988)
Summation	(North and Riley, 1989; Reid and Nygren, 1988; Ismail and Grivard, 2015)
Fourier and inverse Fourier transforms	(Rencken and Durrant-Whyte, 1989)
Decision tree	(Roscoe and Ellis, 1990)
Linear functions	(Rouse et al., 1993)
Standardization through z-scores	(Paas and Van Merriënboer, 1993)
Average	(Entin et al., 1998)
Confirmatory factor analysis	(Yeung et al., 2000)
Neural networks	(Ling et al., 2001; Zhang et al., 2018)
Regression analysis	(Wu and Liu, 2007; Her and Hwang, 1989; Montgomery et al., 1995)
Expert systems	(Rizzo et al., 2016)
Machine learning algorithms	(Moustafa and Longo, 2018; Wilson and Russell, 2003b)

REFERENCES

Salmon P, Stanton N, Walker G, Green D. Situation awareness measurement: A review of applicability for c4i environments. *Applied ergonomics* **37** (2006) 225–238.

Jones R. *Physical Ergonomic And Mental Workload Factors Of Mobile Learning Affecting Performance Of Adult Distance Learners: Student Pers*. Ph.D. thesis, College of Engineering and Computer Science (2009).

Vygotsky LS. *Mind in society: The development of higher psychological processes* (Harvard university press) (1980).

Staal MA. Stress, cognition, and human performance: A literature review and conceptual framework. Tech. rep., Ames Research Center, Moflett Field, California (2004).

Cohen RA. Yerkes–dodson law. *Encyclopedia of clinical neuropsychology* (2011) 2737–2738.

Besson P, Bourdin C, Bringoux L, Dousset E, Maïano C, Marqueste T, et al. Effectiveness of physiological and psychological features to estimate helicopter pilots' workload: A bayesian network approach. *IEEE Transactions on Intelligent Transportation Systems* **14** (2013) 1872–1881.

Zajonc RB. The interaction of affect and cognition. *Approaches to emotion* **239** (1984) 246.

Albers MJ. Tapping as a measure of cognitive load and website usability. *Proceedings of the 29th ACM international conference on Design of communication* (ACM) (2011), 25–32.

Byrne A. Measurement of mental workload in clinical medicine: a review study. *Anesthesiology and pain medicine* **1** (2011) 90.

Dirican AC, Göktürk M. Psychophysiological measures of human cognitive states applied in human computer interaction. *Procedia Computer Science* **3** (2011) 1361–1367.

Galy E, Cariou M, Mélan C. What is the relationship between mental workload factors and cognitive load types? *International Journal of Psychophysiology* **83** (2012) 269–275.

Gwizdka J. Distribution of cognitive load in web search. *Journal of the American Society for Information Science and Technology* **61** (2010) 2167–2187.

Paas F, Tuovinen JE, Tabbers H, Van Gerven PW. Cognitive load measurement as a means to advance cognitive load theory. *Educational psychologist* **38** (2003) 63–71.

Schmutz P, Heinz S, Métrailler Y, Opwis K. Cognitive load in ecommerce applications: measurement and effects on user satisfaction. *Advances in Human-Computer Interaction* **2009** (2009) 3.

Schmutz P, Roth SP, Seckler M, Opwis K. Designing product listing pages—effects on sales and users' cognitive workload. *International journal of human-computer studies* **68** (2010) 423–431.

Tracy JP, Albers MJ. Measuring cognitive load to test the usability of web sites. *Annual Conference-society for technical communication* (2006), vol. 53, 256.

Wiebe EN, Roberts E, Behrend TS. An examination of two mental workload measurement approaches to understanding multimedia learning. *Computers in Human Behavior* **26** (2010) 474–481.

Whelan RR. Neuroimaging of cognitive load in instructional multimedia. *Educational Research Review* **2** (2007) 1–12.

Arguel A, Jamet E. Using video and static pictures to improve learning of procedural contents. *Computers in human behavior* **25** (2009) 354–359.

Antonenko P, Paas F, Grabner R, Van Gog T. Using electroencephalography to measure cognitive load. *Educational Psychology Review* **22** (2010) 425–438.

Byrne A, Tweed N, Halligan C. A pilot study of the mental workload of objective structured clinical examination examiners. *Medical education* **48** (2014) 262–267.

Neßelrath R. Towards a cognitive load aware multimodal dialogue framework for the automotive domain. *2013 9th International Conference on Intelligent Environments* (IEEE) (2013), 266–269.

Walter C, Schmidt S, Rosenstiel W, Gerjets P, Bogdan M. Using cross-task classification for classifying workload levels in complex learning tasks. *2013 Humaine Association Conference on Affective Computing and Intelligent Interaction* (IEEE) (2013), 876–881.

Camp G, Paas F, Rikers R, van Merrienboer J. Dynamic problem selection in air traffic control training: A comparison between performance, mental effort and mental efficiency. *Computers in Human Behavior* **17** (2001) 575–595.

Gavas R, Chatterjee D, Sinha A. Estimation of cognitive load based on the pupil size dilation. *2017 IEEE International Conference on Systems, Man, and Cybernetics (SMC)* (IEEE) (2017), 1499–1504.

Tavares W, Eva KW. Exploring the impact of mental workload on rater-based assessments. *Advances in Health Sciences Education* **18** (2013) 291–303.

Sweller J. Cognitive load theory. *Psychology of learning and motivation* (Elsevier), vol. 55 (2011), 37–76.

Young MS, Stanton NA. Automotive automation: Investigating the impact on drivers' mental workload. *International Journal of Cognitive Ergonomics* **1** (1997) 325–336.

Stanton N. Ecological ergonomics: understanding human action in context. *Contemporary ergonomics* (1995) 62–62.

Saleem JJ, Russ AL, Sanderson P, Johnson TR, Zhang J, Sittig DF. Current challenges and opportunities for better integration of human factors research with development of clinical information systems. *Yearbook of medical informatics* **18** (2009) 48–58.

Hollan J, Hutchins E, Kirsh D. Distributed cognition: toward a new foundation for human-computer interaction research. *ACM Transactions on Computer-Human Interaction (TOCHI)* **7** (2000) 174–196.

Adamczyk PD, Iqbal ST, Bailey BP. A method, system, and tools for intelligent interruption management. *Proceedings of the 4th international workshop on Task models and diagrams* (ACM) (2005), 123–126.

Bailey BP, Konstan JA. On the need for attention-aware systems: Measuring effects of interruption on task performance, error rate, and affective state. *Computers in human behavior* **22** (2006) 685–708.

Johansson G, Hofsten CvH, Jansson G. Event perception. *Annual review of psychology* **31** (1980) 27–63.

Rose CL, Murphy LB, Byard L, Nikzad K. The role of the big five personality factors in vigilance performance and workload. *European Journal of Personality* **16** (2002) 185–200.

Gray JA. A critique of eysenck's theory of personality. *A model for personality* (Springer) (1981), 246–276.

Keller J, Bless H, Blomann F, Kleinböhl D. Physiological aspects of flow experiences: Skills-demand-compatibility effects on heart rate variability and salivary cortisol. *Journal of Experimental Social Psychology* **47** (2011) 849–852.

Csikszentmihalyi M. *Beyond boredom and anxiety.* (Jossey-Bass) (2000).

Li Z, Liu Q, Zhao L. Multi-antenna cognitive uplink design for spectrum reuse. *2011 18th International Conference on Telecommunications* (IEEE) (2011), 44–48.

Bakr O, Johnson M, Wild B, Ramchandran K. A multi-antenna framework for spectrum reuse based on primary-secondary cooperation. *2008 3rd IEEE Symposium on New Frontiers in Dynamic Spectrum Access Networks* (IEEE) (2008), 1–5.

Simon HA. Information-processing theory of human problem solving. *Handbook of learning and cognitive processes* **5** (1978) 271–295.

Herzberg FI. Work and the nature of man. *World* (1966).

Athènes S, Averty P, Puechmorel S, Delahaye D, Collet C. Atc complexity and controller workload: Trying to bridge the gap. *Proceedings of the International Conference on HCI in Aeronautics* (AAAI Press Cambridge, MA) (2002), 56–60.

Cook JR, Salvendy G. Job enrichment and mental workload in computer-based work: Implications for adaptive job design. *International Journal of Industrial Ergonomics* **24** (1999) 13–23.

Hackman JR, Oldham GR. Motivation through the design of work: Test of a theory. *publisheral behavior and human performance* **16** (1976) 250–279.

Basahel AM, Young MS, Ajovalasit M. Impacts of physical and mental workload interaction on human attentional resources performance. *Proceedings of the 28th Annual European Conference on Cognitive Ergonomics* (ACM) (2010), 215–217.

Young MS, Stanton NA. Malleable attentional resources theory: a new explanation for the effects of mental underload on performance. *Human factors* **44** (2002a) 365–375.

Venables L, Fairclough SH. The influence of performance feedback on goal-setting and mental effort regulation. *Motivation and Emotion* **33** (2009) 63–74.

Richter M, Gendolla GH, Wright RA. Three decades of research on motivational intensity theory: What we have learned about effort and what we still don't know. *Advances in motivation science* (Elsevier), vol. 3 (2016), 149–186.

Longo L. A defeasible reasoning framework for human mental workload representation and assessment. *Behaviour & Information Technology* **34** (2015) 758–786.

MacDonald W. Train controller interface design: factors influencing mental workload. *International Conference on People in Control (Human Interfaces in Control Rooms, Cockpits and Command Centres)* (IET) (1999), 31–36.

Mitchell DK. Mental workload and arl workload modeling tools. Tech. rep., Army Research Lab Aberdeen Proving Ground MD (2000).

Prewett MS, Johnson RC, Saboe KN, Elliott LR, Coovert MD. Managing workload in human–robot interaction: A review of empirical studies. *Computers in Human Behavior* **26** (2010) 840–856.

Schulte A, Donath D, Honecker F. Human-system interaction analysis for military pilot activity and mental workload determination. *Systems, Man, and Cybernetics (SMC), 2015 IEEE International Conference on* (IEEE) (2015), 1375–1380.

Verwey WB. Adaptable driver-car interfacing and mental workload: a review of the literature. Tech. rep., Institute of perception RVO-TNO Soesterberg (Netherlands) (1990).

Wickens CD. Multiple resources and mental workload. *Human factors* **50** (2008) 449–455.

Wu C, Tsimhoni O, Liu Y. Development of an adaptive workload management system using the queueing network-model human processor (qn-mhp). *IEEE Transactions on Intelligent Transportation Systems* **9** (2008) 463–475.

Wickens CD. Multiple resources and performance prediction. *Theoretical issues in ergonomics science* **3** (2002) 159–177.

Stanton NA, Hedge A, Brookhuis K, Salas E, Hendrick HW. *Handbook of human factors and ergonomics methods* (CRC press) (2004).

Bommer SC. *Assessing the Effects of Multi-Modal Communications on Mental Workload During the Supervision of Multiple Unmanned Aerial Vehicles*. Master's thesis, Engineering (2013).

Morrison JG. The adaptive function allocation for intelligent cockpits (afaic) program: Interim research and guidelines for the application of adaptive automation. Tech. rep., Naval Air Warfare Center Aircraft DIV Warminster PA (1993).

Moustafa K, Luz S, Longo L. Assessment of mental workload: a comparison of machine learning methods and subjective assessment techniques. *International symposium on human mental workload: Models and applications* (Springer) (2017), 30–50.

Lukanov K, Maior HA, Wilson ML. Using fnirs in usability testing: understanding the effect of web form layout on mental workload. *Proceedings of the 2016 CHI Conference on Human Factors in Computing Systems* (ACM) (2016), 4011–4016.

Mehler B, Reimer B, Zec M. Defining workload in the context of driver state detection and hmi evaluation. *Proceedings of the 4th International Conference on Automotive User Interfaces and Interactive Vehicular Applications* (ACM) (2012), 187–191.

Brown SW. Attentional resources in timing: Interference effects in concurrent temporal and nontemporal working memory tasks. *Perception & psychophysics* **59** (1997) 1118–1140.

Caggiano DM, Parasuraman R. The role of memory representation in the vigilance decrement. *Psychonomic bulletin & review* **11** (2004) 932–937.

Colombi JM, Miller ME, Schneider M, McGrogan MJ, Long CDS, Plaga J. Predictive mental workload modeling for semiautonomous system design: Implications for systems of systems. *Systems Engineering* **15** (2012) 448–460.

Schneider M, McGrogan J, Colombi JM, Miller ME, Long DS. 7.1. 1 modeling pilot workload for multi-aircraft control of an unmanned aircraft system. *INCOSE International Symposium* (Wiley Online Library) (2011), vol. 21, 796–810.

Usui S, Egawa Y. Psycho-physiological analysis of mental workload at an elevated work place. *Japanese Psychological Research* **44** (2002) 152–161.

Zheng B, Cassera MA, Martinec DV, Spaun GO, Swanstrøm LL. Measuring mental workload during the performance of advanced laparoscopic tasks. *Surgical endoscopy* **24** (2010) 45.

Iwata T, Yamabe T, Nakajima T. Towards a mobility enhanced user interface design for multi-task environments: An experimental study on cognitive workload measurement. *2010 Sixth International Conference on Intelligent Environments* (IEEE) (2010), 106–111.

Rusnock CF, Geiger CD. Simulation-based evaluation of adaptive automation revoking strategies on cognitive workload and situation awareness. *IEEE Transactions on Human-Machine Systems* **47** (2017) 927–938.

Brand Y, Schulte A. Model-based prediction of workload for adaptive associate systems. *2017 IEEE International Conference on Systems, Man, and Cybernetics (SMC)* (IEEE) (2017), 1722–1727.

Britt RC, Scerbo MW, Montano M, Kennedy RA, Prytz E, Stefanidis D. Intracorporeal suturing: transfer from fundamentals of laparoscopic surgery to cadavers results in substantial increase in mental workload. *Surgery* **158** (2015) 1428–1433.

Young MS, Stanton NA. Attention and automation: new perspectives on mental underload and performance. *Theoretical Issues in Ergonomics Science* **3** (2002b) 178–194.

Mandrick K, Peysakhovich V, Rémy F, Lepron E, Causse M. Neural and psychophysiological correlates of human performance under stress and high mental workload. *Biological psychology* **121** (2016) 62–73.

Eysenck MW, Calvo MG. Anxiety and performance: The processing efficiency theory. *Cognition & Emotion* **6** (1992) 409–434.

Morris R, MacNeela P, Scott A, Treacy P, Hyde A. Reconsidering the conceptualization of nursing workload: literature review. *Journal of advanced Nursing* **57** (2007) 463–471.

Rasmussen J. Skills, rules, and knowledge; signals, signs, and symbols, and other distinctions in human performance models. *IEEE transactions on systems, man, and cybernetics* **SMC-13** (1983) 257–266.

Ismail DKB, Grivard O. A model-driven approach to the a priori estimation of operator workload. *Cognitive Methods in Situation Awareness and Decision Support (CogSIMA), 2015 IEEE International Inter-Disciplinary Conference on* (IEEE) (2015), 1–7.

Smolka J, Pirker B. International law, pragmatics and the distinction between conceptual and procedural meaning. *Pragmatics and the Distinction between Conceptual and Procedural Meaning (August 14, 2018)* (2018).

Reimer B, Mehler B, Coughlin JF, Wang Y, D'Ambrosio LA, Roy N, et al. A comparison of the effect of a low to moderately demanding cognitive task on simulated driving performance and heart rate in middle aged and young adult drivers. *2008 International Conference on Cyberworlds* (IEEE) (2008), 493–500.

Salthouse T. *A theory of cognitive aging*, vol. 28 (Elsevier) (2000).

Byrne A. Mental workload as a key factor in clinical decision making. *Advances in Health Sciences Education* **18** (2013) 537–545.

Oldfield R. Memory mechanisms and the theory of schemata. *British Journal of Psychology* **45** (1954) 14.

Ma R, Kaber DB. Situation awareness and workload in driving while using adaptive cruise control and a cell phone. *International Journal of Industrial Ergonomics* **35** (2005) 939–953.

Haapalainen E, Kim S, Forlizzi JF, Dey AK. Psycho-physiological measures for assessing cognitive load. *Proceedings of the 12th ACM international conference on Ubiquitous computing* (ACM) (2010), 301–310.

Hancock P, Caird JK. Experimental evaluation of a model of mental workload. *Human factors* **35** (1993) 413–429.

Marquart G, de Winter J. Workload assessment for mental arithmetic tasks using the task-evoked pupillary response. *PeerJ Computer Science* **1** (2015) e16.

Haga S, Shinoda H, Kokubun M. Effects of task difficulty and time-on-task on mental workload. *Japanese Psychological Research* **44** (2002) 134–143.

Liang SFM, Rau CL, Tsai PF, Chen WS. Validation of a task demand measure for predicting mental workloads of physical therapists. *International Journal of Industrial Ergonomics* **44** (2014) 747–752.

Leung GT, Yucel G, Duffy VG. The effects of virtual industrial training on mental workload during task performance. *Human Factors and Ergonomics in Manufacturing & Service Industries* **20** (2010) 567–578.

Mizobuchi S, Chignell M, Newton D. Mobile text entry: relationship between walking speed and text input task difficulty. *Proceedings of the 7th international conference on Human computer interaction with mobile devices & services* (ACM) (2005), 122–128.

Palinko O, Kun AL, Shyrokov A, Heeman P. Estimating cognitive load using remote eye tracking in a driving simulator. *Proceedings of the 2010 symposium on eye-tracking research & applications* (ACM) (2010), 141–144.

Cain B. A review of the mental workload literature. Tech. rep., Defence Research And Development Toronto (Canada) (2007).

Di Stasi LL, Antolí A, Cañas JJ. Evaluating mental workload while interacting with computer-generated artificial environments. *Entertainment Computing* **4** (2013a) 63–69.

Wästlund E. *Experimental studies of human-computer interaction: Working memory and mental workload in complex cognition* (Department of Psychology) (2007).

Annett J. Subjective rating scales: science or art? *Ergonomics* **45** (2002) 966–987.

Stuiver A, Brookhuis KA, de Waard D, Mulder B. Short-term cardiovascular measures for driver support: increasing sensitivity for detecting changes in mental workload. *International Journal of Psychophysiology* **92** (2014) 35–41.

Wilson GF, Eggemeier FT. Mental workload measurement. *International encyclopedia of ergonomics and human factors* **1** (2006).

Byrne A, Oliver M, Bodger O, Barnett W, Williams D, Jones H, et al. Novel method of measuring the mental workload of anaesthetists during clinical practice. *British journal of anaesthesia* **105** (2010) 767–771.

Baldauf D, Burgard E, Wittmann M. Time perception as a workload measure in simulated car driving. *Applied ergonomics* **40** (2009) 929–935.

Hou X, Liu Y, Sourina O, Mueller-Wittig W. Cognimeter: Eeg-based emotion, mental workload and stress visual monitoring. *Cyberworlds (CW), 2015 International Conference on* (IEEE) (2015), 153–160.

Paas FG, Van Merriënboer JJ. The efficiency of instructional conditions: An approach to combine mental effort and performance measures. *Human factors* **35** (1993) 737–743.

Chen J, Song X, Lin Z. Revealing the "invisible gorilla" in construction: Estimating construction safety through mental workload assessment. *Automation in Construction* **63** (2016) 173–183.

Stock AK, Riegler L, Chmielewski WX, Beste C. Paradox effects of binge drinking on response inhibition processes depending on mental workload. *Archives of toxicology* **90** (2016) 1429–1436.

Fréard D, Jamet E, Le Bohec O, Poulain G, Botherel V. Subjective measurement of workload related to a multimodal interaction task: Nasa-tlx vs. workload profile. *International Conference on Human-Computer Interaction* (Springer) (2007), 60–69.

Weinger MB, Reddy SB, Slagle JM. Multiple measures of anesthesia workload during teaching and nonteaching cases. *Anesthesia & Analgesia* **98** (2004) 1419–1425.

Lim WL, Sourina O, Liu Y, Wang L. Eeg-based mental workload recognition related to multitasking. *Information, Communications and Signal Processing (ICICS), 2015 10th International Conference on* (IEEE) (2015), 1–4.

Miller S. Workload measures. *National Advanced Driving Simulator. Iowa City, United States* (2001).

Wang S, Gwizdka J, Chaovalitwongse WA. Using wireless eeg signals to assess memory workload in the n-back task. *IEEE Transactions on Human-Machine Systems* **46** (2016) 424–435.

Young G, Zavelina L, Hooper V. Assessment of workload using nasa task load index in perianesthesia nursing. *Journal of PeriAnesthesia Nursing* **23** (2008) 102–110.

Lin CJ, Hsieh TL, Tsai PJ, Yang CW, Yenn TC. Development of a team workload assessment technique for the main control room of advanced nuclear power plants. *Human Factors and Ergonomics in Manufacturing & Service Industries* **21** (2011) 397–411.

Kum S, Furusho M, Duru O, Satir T. Mental workload of the vts operators by utilising heart rate. *TransNav, International Journal on Marine Navigation and Safety od Sea Transportation* **1** (2007).

Harriott CE, Buford GL, Adams JA, Zhang T. Mental workload and task performance in peer-based human-robot teams. *Journal of Human-Robot Interaction* **4** (2015) 61–96.

Omolayo O, Omole C. Influence of mental workload on job performance. *International Journal of Humanities and Social Science* **3** (2013) 238–246.

Pierce ET. *Mental workload measurement using the intersaccadic interval*. Ph.D. thesis, Biomedical Engineering (2009).

Kim HS, Hwang Y, Yoon D, Choi W, Park CH. Driver workload characteristics analysis using eeg data from an urban road. *IEEE Transactions on Intelligent Transportation Systems* **15** (2014) 1844–1849.

Alexander AL, Nygren TE, Vidulich MA. Examining the relationship between mental workload and situation awareness in a simulated air combat task. Tech. rep., Ohio State Univ Columbus Dept of Psychology (2000).

Borghini G, Astolfi L, Vecchiato G, Mattia D, Babiloni F. Measuring neurophysiological signals in aircraft pilots and car drivers for the assessment of mental workload, fatigue and drowsiness. *Neuroscience & Biobehavioral Reviews* **44** (2014) 58–75.

Brouwer AM, Hogervorst MA, Van Erp JB, Heffelaar T, Zimmerman PH, Oostenveld R. Estimating workload using eeg spectral power and erps in the n-back task. *Journal of neural engineering* **9** (2012) 045008.

Lodree Jr EJ, Geiger CD, Jiang X. Taxonomy for integrating scheduling theory and human factors: Review and research opportunities. *International Journal of Industrial Ergonomics* **39** (2009) 39–51.

Frey J, Mühl C, Lotte F, Hachet M. Review of the use of electroencephalography as an evaluation method for human-computer interaction. *arXiv preprint arXiv:1311.2222* (2013).

Hu JS, Lu J, Tan WB, Lomanto D. Training improves laparoscopic tasks performance and decreases operator workload. *Surgical endoscopy* **30** (2016) 1742–1746.

Rizzo L, Dondio P, Delany SJ, Longo L. Modeling mental workload via rule-based expert system: a comparison with nasa-tlx and workload profile. *IFIP International Conference on Artificial Intelligence Applications and Innovations* (Springer) (2016), 215–229.

Longo L. Mental workload in medicine: foundations, applications, open problems, challenges and future perspectives. *2016 IEEE 29th International Symposium on Computer-Based Medical Systems (CBMS)* (IEEE) (2016), 106–111.

Longo L, Dondio P. On the relationship between perception of usability and subjective mental workload of web interfaces. *Web Intelligence and Intelligent Agent Technology (WI-IAT), 2015 IEEE/WIC/ACM International Conference on* (IEEE) (2015), vol. 1, 345–352.

Kum S, Furusho M, Fuchi M. Assessment of vts operators' mental workload by using nasa task load index. *the Journal of Japan Institute of Navigation* **118** (2008a) 307–314.

Carswell CM, Clarke D, Seales WB. Assessing mental workload during laparoscopic surgery. *Surgical innovation* **12** (2005) 80–90.

Sheridan TB, Simpson R. Toward the definition and measurement of the mental workload of transport pilots. Tech. rep., Cambridge, Mass.: Massachusetts Institute of Technology, Dept. of Aeronautics and Astronautics, Flight Transportation Laboratory,[1979] (1979).

Xie B, Salvendy G. Review and reappraisal of modelling and predicting mental workload in single-and multi-task environments. *Work & stress* **14** (2000a) 74–99.

Smiley A. Mental workload and information management. *Vehicle Navigation and Information Systems Conference, 1989. Conference Record* (IEEE) (1989), 435–438.

Young MS, Brookhuis KA, Wickens CD, Hancock PA. State of science: mental workload in ergonomics. *Ergonomics* **58** (2015) 1–17.

Young M, Stanton N. Mental workload: theory, measurement, and application. *International encyclopedia of ergonomics and human factors* **1** (2001) 507–509.

Neill D. Nursing workload and the changing health care environment: a review of the literature. *Administrative Issues Journal* **1** (2011) 13.

Potter SS, Bressler JR. Subjective workload assessment technique (swat): a user's guide. Tech. rep., Systems Research Labs Inc Dayton OH (1989).

Carter JR, Kupiers NT, Ray CA. Neurovascular responses to mental stress. *The Journal of physiology* **564** (2005) 321–327.

Olsson S, Burns P. Measuring driver visual distraction with a peripheral detection task. *Obtained from August* (2000).

Brookhuis KA, van Driel CJ, Hof T, van Arem B, Hoedemaeker M. Driving with a congestion assistant; mental workload and acceptance. *Applied ergonomics* **40** (2009) 1019–1025.

Johnson A, Widyanti A. Cultural influences on the measurement of subjective mental workload. *Ergonomics* **54** (2011) 509–518.

Tørnros JE, Bolling AK. Mobile phone use—effects of handheld and handsfree phones on driving performance. *Accident Analysis & Prevention* **37** (2005) 902–909.

Veltman J, Gaillard A. Physiological workload reactions to increasing levels of task difficulty. *Ergonomics* **41** (1998) 656–669.

Lin Y, Zhang W, Watson LG. Using eye movement parameters for evaluating human–machine interface frameworks under normal control operation and fault detection situations. *International Journal of Human-Computer Studies* **59** (2003) 837–873.

Banerjee J, Majumdar D, Pal MS, Majumdar D. Readability, subjective preference and mental workload studies on young indian adults for selection of optimum font type and size during onscreen reading. *Al Ameen Journal of Medical Sciences* **4** (2011) 131–143.

Chaouachi M, Jraidi I, Frasson C. Modeling mental workload using eeg features for intelligent systems. *International Conference on User Modeling, Adaptation, and Personalization* (Springer) (2011), 50–61.

Cinaz B, Arnrich B, Marca R, Tröster G. Monitoring of mental workload levels during an everyday life office-work scenario. *Personal and ubiquitous computing* **17** (2013) 229–239.

Matthews G, Reinerman-Jones LE, Barber DJ, Abich IV J. The psychometrics of mental workload: multiple measures are sensitive but divergent. *Human Factors* **57** (2015) 125–143.

Darvishi E, Maleki A, Giahi O, Akbarzadeh A. Subjective mental workload and its correlation with musculoskeletal disorders in bank staff. *Journal of manipulative and physiological therapeutics* **39** (2016) 420–426.

Gómez-Gómez E, Carrasco-Valiente J, Valero-Rosa J, Campos-Hernandez J, Anglada-Curado F, Carazo-Carazo J, et al. Impact of 3d vision on mental workload and laparoscopic performance in inexperienced subjects. *Actas Urológicas Españolas (English Edition)* **39** (2015) 229–235.

Hancock P. The effect of gender and time of day upon the subjective estimate of mental workload during the performance of a simple task. *Advances in Psychology* (Elsevier), vol. 52 (1988), 239–250.

Hancock P. The effect of performance failure and task demand on the perception of mental workload. *Applied Ergonomics* **20** (1989) 197–205.

Hancock P, Williams G, Manning C. Influence of task demand characteristics on workload and performance. *The International Journal of Aviation Psychology* **5** (1995) 63–86.
Harris WC, Hancock PA, Arthur EJ, Caird JK. Performance, workload, and fatigue changes associated with automation. *The International Journal of Aviation Psychology* **5** (1995) 169–185.
Hoover A, Singh A, Fishel-Brown S, Muth E. Real-time detection of workload changes using heart rate variability. *Biomedical Signal Processing and Control* **7** (2012) 333–341.
Hwang SL, Yau YJ, Lin YT, Chen JH, Huang TH, Yenn TC, et al. A mental workload predicator model for the design of pre alarm systems. *International Conference on Engineering Psychology and Cognitive Ergonomics* (Springer) (2007), 316–323.
Jahn G, Oehme A, Krems JF, Gelau C. Peripheral detection as a workload measure in driving: Effects of traffic complexity and route guidance system use in a driving study. *Transportation Research Part F: Traffic Psychology and Behaviour* **8** (2005) 255–275.
Jou YT, Yenn TC, Lin CJ, Yang CW, Chiang CC. Evaluation of operators' mental workload of human–system interface automation in the advanced nuclear power plants. *Nuclear Engineering and Design* **239** (2009) 2537–2542.
Kataoka J, Sasaki M, Kanda K. Effects of mental workload on nurses' visual behaviors during infusion pump operation. *Japan Journal of Nursing Science* **8** (2011) 47–56.
Kawakita E, Itoh M, Oguri K. Estimation of driver's mental workload using visual information and heart rate variability. *Intelligent Transportation Systems (ITSC), 2010 13th International IEEE Conference on* (IEEE) (2010), 765–769.
Kiselev A, Loutfi A. Using a mental workload index as a measure of usability of a user interface for social robotic telepresence. *2nd Workshop of Social Robotic Telepresence in Conjunction with IEEE International Symposium on Robot and Human Interactive Communication 2012* (IEEE) (2012).
Kjeldskov J, Stage J. New techniques for usability evaluation of mobile systems. *International journal of human-computer studies* **60** (2004) 599–620.
Kokini CM, Lee S, Koubek RJ, Moon SK. Considering context: The role of mental workload and operator control in users' perceptions of usability. *International Journal of Human-Computer Interaction* **28** (2012) 543–559.
Mayes DK, Sims VK, Koonce JM. Comprehension and workload differences for vdt and paper-based reading. *International Journal of Industrial Ergonomics* **28** (2001) 367–378.
Mayser C, Piechulla W, Weiss KE, Kønig W. Driver workload monitoring. *Proceedings of the Internationale Ergonomie-Konferenz der GfA, ISOES und FEES* (2003), 7–9.
Miyake S. Multivariate workload evaluation combining physiological and subjective measures. *International journal of psychophysiology* **40** (2001) 233–238.
Miyake S, Yamada S, Shoji T, Takae Y, Kuge N, Yamamura T. Physiological responses to workload change. a test/retest examination. *Applied ergonomics* **40** (2009) 987–996.
Moroney WF, Biers DW, Eggemeier FT, Mitchell JA. A comparison of two scoring procedures with the nasa task load index in a simulated flight task. *Aerospace and electronics conference, 1992. NAECON 1992., proceedings of the IEEE 1992 national* (IEEE) (1992), 734–740.
Noyes JM, Bruneau DP. A self-analysis of the nasa-tlx workload measure. *Ergonomics* **50** (2007) 514–519.
Tremoulet PD, Craven PL, Regli SH, Wilcox S, Barton J, Stibler K, et al. Workload-based assessment of a user interface design. *International Conference on Digital Human Modeling* (Springer) (2009), 333–342.
Trujillo AC. Pilot mental workload with predictive system status information. *Human Interaction with Complex Systems, 1998. Proceedings., Fourth Annual Symposium on* (IEEE) (1998), 73–80.
Vera J, Jiménez R, García JA, Cárdenas D. Intraocular pressure is sensitive to cumulative and instantaneous mental workload. *Applied ergonomics* **60** (2017) 313–319.
Vitório DM, Masculo FS, Melo MO. Analysis of mental workload of electrical power plant operators of control and operation centers. *Work* **41** (2012) 2831–2839.
Wu C, Liu Y. Queuing network modeling of driver workload and performance. *IEEE Transactions on Intelligent Transportation Systems* **8** (2007) 528–537.
Piechulla W, Mayser C, Gehrke H, Kønig W. Reducing drivers' mental workload by means of an adaptive man–machine interface. *Transportation Research Part F: Traffic Psychology and Behaviour* **6** (2003) 233–248.
Riccio A, Leotta F, Bianchi L, Aloise F, Zickler C, Hoogerwerf E, et al. Workload measurement in a communication application operated through a p300-based brain–computer interface. *Journal of Neural Engineering* **8** (2011) 025028.

Safari S, Akbari J, Kazemi M, Mououdi MA, Mahaki B. Personnel's health surveillance at work: effect of age, body mass index, and shift work on mental workload and work ability index. *Journal of environmental and public health* **2013** (2013).

Shinohara K, Miura T, Usui S. Tapping task as an index of mental workload in a time sharing task 1. *Japanese psychological research* **44** (2002) 144–151.

Singh AL, Tiwari T, Singh IL. Effects of automation reliability and training on automation-induced complacency and perceived mental workload. *Journal of the Indian Academy of Applied Psychology* **35** (2009) 9–22.

Singh AL, Tiwari T, Singh IL. Performance feedback, mental workload and monitoring efficiency. *J Indian Acad Appl Psychol* **36** (2010) 151–8.

Stefanidis D, Haluck R, Pham T, Dunne JB, Reinke T, Markley S, et al. Construct and face validity and task workload for laparoscopic camera navigation: virtual reality versus videotrainer systems at the sages learning center. *Surgical endoscopy* **21** (2007) 1158–1164.

Svensson E, Angelborg-Thanderez M, Sjøberg L, Olsson S. Information complexity-mental workload and performance in combat aircraft. *Ergonomics* **40** (1997) 362–380.

Xie B, Salvendy G. Prediction of mental workload in single and multiple tasks environments. *International journal of cognitive ergonomics* **4** (2000b) 213–242.

Harbluk JL, Noy YI, Trbovich PL, Eizenman M. An on-road assessment of cognitive distraction: Impacts on drivers' visual behavior and braking performance. *Accident Analysis & Prevention* **39** (2007) 372–379.

Ikuma LH, Harvey C, Taylor CF, Handal C. A guide for assessing control room operator performance using speed and accuracy, perceived workload, situation awareness, and eye tracking. *Journal of loss prevention in the process industries* **32** (2014) 454–465.

Knaepen K, Marusic U, Crea S, Guerrero CDR, Vitiello N, Pattyn N, et al. Psychophysiological response to cognitive workload during symmetrical, asymmetrical and dual-task walking. *Human movement science* **40** (2015) 248–263.

Huber RK, Eggenhofer P, Romer J, Schafer S, Titze K. Coalition command and control in the networked era. *11th ICCRTS* (2006).

Chen JY, Barnes MJ, Harper-Sciarini M. Supervisory control of multiple robots: Human-performance issues and user-interface design. *IEEE transactions on systems, man and cybernetics, part C: applications and reviews* **41** (2011a) 435–454.

Kang Y, Wang M, Lin R. A study of e-book operation in usability and mental workload. *HAAMAHA–2004.–9th* (2004) 489–496.

Tokunaga RA, Shimojo A, Hagiwara T, Kagaya S, Uchida Ke. Effects of cellular telephone use while driving based on objective and subjective mental workload assessment. *Driving Assessment 2001: The First International Driving Symposium on Human Factors in Driver Assessment, Training and Vehicle Design Location* (University of Iowa) (2001), 112–117.

Barnard L, Yi JS, Jacko JA, Sears A. Capturing the effects of context on human performance in mobile computing systems. *Personal and Ubiquitous Computing* **11** (2007) 81–96.

Dey A, Mann DD. Sensitivity and diagnosticity of nasa-tlx and simplified swat to assess the mental workload associated with operating an agricultural sprayer. *Ergonomics* **53** (2010) 848–857.

Colle HA, Reid GB. Context effects in subjective mental workload ratings. *Human factors* **40** (1998) 591–600.

Moustafa K, Longo L. Analysing the impact of machine learning to model subjective mental workload: A case study in third-level education. *International Symposium on Human Mental Workload: Models and Applications* (Springer) (2018), 92–111.

Tungare M, Pérez-Quiñones MA. Mental workload in multi-device personal information management. *CHI'09 Extended Abstracts on Human Factors in Computing Systems* (ACM) (2009), 3431–3436.

Mark G, Gudith D, Klocke U. The cost of interrupted work: more speed and stress. *Proceedings of the SIGCHI conference on Human Factors in Computing Systems* (ACM) (2008), 107–110.

Schneegass S, Pfleging B, Broy N, Heinrich F, Schmidt A. A data set of real world driving to assess driver workload. *Proceedings of the 5th international conference on automotive user interfaces and interactive vehicular applications* (ACM) (2013), 150–157.

Adamczyk PD, Bailey BP. If not now, when?: the effects of interruption at different moments within task execution. *Proceedings of the SIGCHI conference on Human factors in computing systems* (ACM) (2004), 271–278.

Nielsen CM, Overgaard M, Pedersen MB, Stage J, Stenild S. It's worth the hassle!: the added value of evaluating the usability of mobile systems in the field. *Proceedings of the 4th Nordic conference on Human-computer interaction: changing roles* (ACM) (2006), 272–280.

Fritz T, Begel A, Müller SC, Yigit-Elliott S, Züger M. Using psycho-physiological measures to assess task difficulty in software development. *Proceedings of the 36th International Conference on Software Engineering* (ACM) (2014), 402–413.

Fairclough SH, Venables L, Tattersall A. The influence of task demand and learning on the psychophysiological response. *International Journal of Psychophysiology* **56** (2005) 171–184.

Newell GS, Mansfield NJ. Evaluation of reaction time performance and subjective workload during whole-body vibration exposure while seated in upright and twisted postures with and without armrests. *International Journal of Industrial Ergonomics* **38** (2008) 499–508.

Wang X, Dunston PS. Compatibility issues in augmented reality systems for aec: An experimental prototype study. *Automation in construction* **15** (2006) 314–326.

France DJ, Levin S, Hemphill R, Chen K, Rickard D, Makowski R, et al. Emergency physicians' behaviors and workload in the presence of an electronic whiteboard. *International journal of medical informatics* **74** (2005) 827–837.

Colligan L, Potts HW, Finn CT, Sinkin RA. Cognitive workload changes for nurses transitioning from a legacy system with paper documentation to a commercial electronic health record. *International journal of medical informatics* **84** (2015) 469–476.

Rani P, Sarkar N, Adams J. Anxiety-based affective communication for implicit human–machine interaction. *Advanced Engineering Informatics* **21** (2007) 323–334.

Baulk SD, Kandelaars KJ, Lamond N, Roach GD, Dawson D, Fletcher A. Does variation in workload affect fatigue in a regular 12-hour shift system? *Sleep and Biological Rhythms* **5** (2007) 74–77.

Bradley NA, Dunlop MD. An experimental investigation into wayfinding directions for visually impaired people. *Personal and Ubiquitous Computing* **9** (2005) 395–403.

Engelmann C, Schneider M, Kirschbaum C, Grote G, Dingemann J, Schoof S, et al. Effects of intraoperative breaks on mental and somatic operator fatigue: a randomized clinical trial. *Surgical endoscopy* **25** (2011) 1245–1250.

Epling SL, Russell PN, Helton WS. A new semantic vigilance task: vigilance decrement, workload, and sensitivity to dual-task costs. *Experimental brain research* **234** (2016) 133–139.

Guznov S, Matthews G, Funke G, Dukes A. Use of the roboflag synthetic task environment to investigate workload and stress responses in uav operation. *Behavior research methods* **43** (2011) 771–780.

Helton WS, Hollander TD, Warm JS, Matthews G, Dember WN, Wallaart M, et al. Signal regularity and the mindlessness model of vigilance. *British Journal of Psychology* **96** (2005) 249–261.

Hubert N, Gilles M, Desbrosses K, Meyer J, Felblinger J, Hubert J. Ergonomic assessment of the surgeon's physical workload during standard and robotic assisted laparoscopic procedures. *The International Journal of Medical Robotics and Computer Assisted Surgery* **9** (2013) 142–147.

Fisher SL, Ford JK. Differential effects of learner effort and goal orientation on two learning outcomes. *Personnel Psychology* **51** (1998) 397–420.

Kajiwara S. Evaluation of driver's mental workload by facial temperature and electrodermal activity under simulated driving conditions. *International Journal of Automotive Technology* **15** (2014) 65–70.

Liang GF, Lin JT, Hwang SL, Huang Fh, Yenn TC, Hsu CC. Evaluation and prediction of on-line maintenance workload in nuclear power plants. *Human Factors and Ergonomics in Manufacturing & Service Industries* **19** (2009) 64–77.

Lin L, Isla R, Doniz K, Harkness H, Vicente KJ, Doyle DJ. Applying human factors to the design of medical equipment: patient-controlled analgesia. *Journal of clinical monitoring and computing* **14** (1998) 253–263.

Luz M, Manzey D, Mueller S, Dietz A, Meixensberger J, Strauss G. Impact of navigated-control assistance on performance, workload and situation awareness of experienced surgeons performing a simulated mastoidectomy. *The International Journal of Medical Robotics and Computer Assisted Surgery* **10** (2014) 187–195.

Rebetez C, Bétrancourt M, Sangin M, Dillenbourg P. Learning from animation enabled by collaboration. *Instructional science* **38** (2010) 471–485.

Rubio S, Díaz E, Martín J, Puente JM. Evaluation of subjective mental workload: A comparison of swat, nasa-tlx, and workload profile methods. *Applied Psychology* **53** (2004) 61–86.

Ruiz-Rabelo JF, Navarro-Rodriguez E, Di-Stasi LL, Diaz-Jimenez N, Cabrera-Bermon J, Diaz-Iglesias C, et al. Validation of the nasa-tlx score in ongoing assessment of mental workload during a laparoscopic learning curve in bariatric surgery. *Obesity surgery* **25** (2015) 2451–2456.

Di Stasi LL, McCamy MB, Catena A, Macknik SL, Canas JJ, Martinez-Conde S. Microsaccade and drift dynamics reflect mental fatigue. *European Journal of Neuroscience* **38** (2013b) 2389–2398.

Zheng B, Jiang X, Tien G, Meneghetti A, Panton ONM, Atkins MS. Workload assessment of surgeons: correlation between nasa tlx and blinks. *Surgical endoscopy* **26** (2012) 2746–2750.

Putze F, Jarvis JP, Schultz T. Multimodal recognition of cognitive workload for multitasking in the car. *Pattern Recognition (ICPR), 2010 20th International Conference on* (IEEE) (2010), 3748–3751.

Lin B, Wu C. Mathematical modeling of the human cognitive system in two serial processing stages with its applications in adaptive workload-management systems. *IEEE Transactions on Intelligent Transportation Systems* **12** (2011) 221–231.

Zhang H, Zhu Y, Maniyeri J, Guan C. Detection of variations in cognitive workload using multi-modality physiological sensors and a large margin unbiased regression machine. *Engineering in Medicine and Biology Society (EMBC), 2014 36th Annual International Conference of the IEEE* (IEEE) (2014), 2985–2988.

Makhtar AK, Ab Patar MNAB, Ramli MHM, Mahat MM, Zubair AF, Lukman H. A study on the effects of the cognitive workload on the driver's blood pulse wave. *Computer Applications and Industrial Electronics (ICCAIE), 2011 IEEE International Conference on* (IEEE) (2011), 300–302.

Besson P, Maïano C, Bringoux L, Marqueste T, Mestre DR, Bourdin C, et al. Cognitive workload and affective state: A computational study using bayesian networks. *2012 6th IEEE International Conference Intelligent Systems* (IEEE) (2012a), 140–145.

Gentili RJ, Rietschel JC, Jaquess KJ, Lo LC, Prevost CM, Miller MW, et al. Brain biomarkers based assessment of cognitive workload in pilots under various task demands. *2014 36th Annual International Conference of the IEEE Engineering in Medicine and Biology Society* (IEEE) (2014), 5860–5863.

Borghini G, Aricò P, Di Flumeri G, Salinari S, Colosimo A, Bonelli S, et al. Avionic technology testing by using a cognitive neurometric index: a study with professional helicopter pilots. *2015 37th Annual International Conference of the IEEE Engineering in Medicine and Biology Society (EMBC)* (IEEE) (2015), 6182–6185.

Zhang Y, Zheng H, Duan Y, Meng L, Zhang L. An integrated approach to subjective measuring commercial aviation pilot workload. *2015 IEEE 10th Conference on Industrial Electronics and Applications (ICIEA)* (IEEE) (2015a), 1093–1098.

Liang Y, Liang W, Qu J, Yang J. Experimental study on eeg with different cognitive load. *2018 IEEE International Conference on Systems, Man, and Cybernetics (SMC)* (IEEE) (2018), 4351–4356.

Entin EE, MacMillan J, Serfaty D. Analysis of publisheral processes in adaptive command and control architectures. *SMC'98 Conference Proceedings. 1998 IEEE International Conference on Systems, Man, and Cybernetics (Cat. No. 98CH36218)* (IEEE) (1998), vol. 4, 3664–3668.

Won JC, Condon GR, Landon BR, Wang AR, Hannon DJ. Assessing team workload and situational awareness in an intelligence, surveillance, and reconnaissance (isr) simulation exercise. *2011 IEEE International Multi-Disciplinary Conference on Cognitive Methods in Situation Awareness and Decision Support (CogSIMA)* (IEEE) (2011), 163–167.

Yanghua C, Fansen K. A forecast model about average load of cognitive work based on bp network. *Proceedings 2011 International Conference on Transportation, Mechanical, and Electrical Engineering (TMEE)* (IEEE) (2011), 347–350.

Besson P, Dousset E, Bourdin C, Bringoux L, Marqueste T, Mestre D, et al. Bayesian network classifiers inferring workload from physiological features: Compared performance. *2012 IEEE Intelligent Vehicles Symposium* (IEEE) (2012b), 282–287.

Bodala IP, Ke Y, Mir H, Thakor NV, Al-Nashash H. Cognitive workload estimation due to vague visual stimuli using saccadic eye movements. *2014 36th Annual International Conference of the IEEE Engineering in Medicine and Biology Society* (IEEE) (2014), 2993–2996.

Durkee KT, Pappada SM, Ortiz AE, Feeney JJ, Galster SM. System decision framework for augmenting human performance using real-time workload classifiers. *2015 IEEE International Multi-Disciplinary Conference on Cognitive Methods in Situation Awareness and Decision* (IEEE) (2015), 8–13.

Krausman AS. Understanding audio communication delay in distributed team interaction: Impact on trust, shared understanding, and workload. *2017 IEEE Conference on Cognitive and Computational Aspects of Situation Management (CogSIMA)* (IEEE) (2017), 1–3.

Villa R, Halvey M. Is relevance hard work?: evaluating the effort of making relevant assessments. *Proceedings of the 36th international ACM SIGIR conference on Research and development in information retrieval* (ACM) (2013), 765–768.
Lan L, Lian Z, Pan L. The effects of air temperature on office workers' well-being, workload and productivity-evaluated with subjective ratings. *Applied ergonomics* **42** (2010) 29–36.
Young MS, Mahfoud JM, Stanton NA, Salmon PM, Jenkins DP, Walker GH. Conflicts of interest: the implications of roadside advertising for driver attention. *Transportation research part F: traffic psychology and behaviour* **12** (2009) 381–388.
Stefanidis D, Wang F, Korndorffer JR, Dunne JB, Scott DJ. Robotic assistance improves intracorporeal suturing performance and safety in the operating room while decreasing operator workload. *Surgical endoscopy* **24** (2010) 377–382.
Carayon P, Gürses AP. A human factors engineering conceptual framework of nursing workload and patient safety in intensive care units. *Intensive and Critical Care Nursing* **21** (2005) 284–301.
Colle HA, Reid GB. Estimating a mental workload redline in a simulated air-to-ground combat mission. *The International Journal of Aviation Psychology* **15** (2005) 303–319.
Luximon A, Goonetilleke RS. Continuous subjective workload assessment technique. *First World Congress on Ergonomics for Global Quality and Productivity, Hong Kong* (1998).
Luximon A, Goonetilleke RS. Simplified subjective workload assessment technique. *Ergonomics* **44** (2001) 229–243.
Pickup L, Wilson JR, Norris BJ, Mitchell L, Morrisroe G. The integrated workload scale (iws): a new self-report tool to assess railway signaller workload. *Applied Ergonomics* **36** (2005) 681–693.
Wittmann M, Kiss M, Gugg P, Steffen A, Fink M, Pøppel E, et al. Effects of display position of a visual in-vehicle task on simulated driving. *Applied Ergonomics* **37** (2006) 187–199.
Roscoe AH, Ellis GA. A subjective rating scale for assessing pilot workload in flight: A decade of practical use. Tech. rep., Royal Aerospace Establishment Farnborough (United Kingdom) (1990).
Roy RN, Charbonnier S, Campagne A, Bonnet S. Efficient mental workload estimation using task-independent eeg features. *Journal of neural engineering* **13** (2016) 026019.
Elmenhorst EM, Vejvoda M, Maass H, Wenzel J, Plath G, Schubert E, et al. Pilot workload during approaches: comparison of simulated standard and noise-abatement profiles. *Aviation, space, and environmental medicine* **80** (2009) 364–370.
Tsang PS, Velazquez VL. Diagnosticity and multidimensional subjective workload ratings. *Ergonomics* **39** (1996) 358–381.
KAKIZAKI T. Sex differences in mental workload during performance of mental tasks. *Industrial health* **25** (1987) 183–194.
Collins SM, Karasek RA, Costas K. Job strain and autonomic indices of cardiovascular disease risk. *American journal of industrial medicine* **48** (2005) 182–193.
Wierwille WW, Rahimi M, Casali JG. Evaluation of 16 measures of mental workload using a simulated flight task emphasizing mediational activity. *Human Factors* **27** (1985) 489–502.
Brayda L, Campus C, Memeo M, Lucagrossi L. The importance of visual experience, gender, and emotion in the assessment of an assistive tactile mouse. *IEEE transactions on haptics* **8** (2015) 279–286.
Guastello SJ, Boeh H, Shumaker C, Schimmels M. Catastrophe models for cognitive workload and fatigue. *Theoretical Issues in Ergonomics Science* **13** (2012) 586–602.
Arico P, Borghini G, Di Flumeri G, Colosimo A, Graziani I, Imbert JP, et al. Reliability over time of eeg-based mental workload evaluation during air traffic management (atm) tasks. *2015 37th Annual International Conference of the IEEE Engineering in Medicine and Biology Society (EMBC)* (IEEE) (2015), 7242–7245.
Aricò P, Borghini G, Di Flumeri G, Colosimo A, Bonelli S, Golfetti A, et al. Adaptive automation triggered by eeg-based mental workload index: a passive brain-computer interface application in realistic air traffic control environment. *Frontiers in human neuroscience* **10** (2016) 539.
Berka C, Levendowski DJ, Lumicao MN, Yau A, Davis G, Zivkovic VT, et al. Eeg correlates of task engagement and mental workload in vigilance, learning, and memory tasks. *Aviation, space, and environmental medicine* **78** (2007) B231–B244.
Brookings JB, Wilson GF, Swain CR. Psychophysiological responses to changes in workload during simulated air traffic control. *Biological psychology* **42** (1996) 361–377.
Cartocci G, Maglione AG, Vecchiato G, Di Flumeri G, Colosimo A, Scorpecci A, et al. Mental workload estimations in unilateral deafened children. *Engineering in Medicine and Biology Society (EMBC), 2015 37th Annual International Conference of the IEEE* (IEEE) (2015), 1654–1657.

Aghajani H, Garbey M, Omurtag A. Measuring mental workload with eeg+ fnirs. *Frontiers in human neuroscience* **11** (2017) 359.

Dussault C, Jouanin JC, Philippe M, Guezennec CY. Eeg and ecg changes during simulator operation reflect mental workload and vigilance. *Aviation, space, and environmental medicine* **76** (2005) 344–351.

Ling C, Goins H, Ntuen A, Li R. Eeg signal analysis for human workload classification. *SoutheastCon 2001. Proceedings. IEEE* (IEEE) (2001), 123–130.

Mak JN, Chan RH, Wong SW. Evaluation of mental workload in visual-motor task: Spectral analysis of single-channel frontal eeg. *Industrial Electronics Society, IECON 2013-39th Annual Conference of the IEEE* (IEEE) (2013), 8426–8430.

Mazaeva N, Ntuen C, Lebby G. Self-organizing map (som) model for mental workload classification. *IFSA World Congress and 20th NAFIPS International Conference, 2001. Joint 9th* (IEEE) (2001), vol. 3, 1822–1825.

Roy RN, Bonnet S, Charbonnier S, Jallon P, Campagne A. A comparison of erp spatial filtering methods for optimal mental workload estimation. *Engineering in Medicine and Biology Society (EMBC), 2015 37th Annual International Conference of the IEEE* (IEEE) (2015), 7254–7257.

Ryu K, Myung R. Evaluation of mental workload with a combined measure based on physiological indices during a dual task of tracking and mental arithmetic. *International Journal of Industrial Ergonomics* **35** (2005) 991–1009.

Krol LR, Freytag SC, Fleck M, Gramann K, Zander TO. A task-independent workload classifier for neuroadaptive technology: Preliminary data. *2016 IEEE International Conference on Systems, Man, and Cybernetics (SMC)* (IEEE) (2016), 003171–003174.

Wanyan X, Zhuang D, Zhang H. Improving pilot mental workload evaluation with combined measures. *Bio-medical materials and engineering* **24** (2014) 2283–2290.

Wilson GF, Russell CA. Operator functional state classification using multiple psychophysiological features in an air traffic control task. *Human Factors* **45** (2003a) 381–389.

Yin Z, Zhang J. Cross-session classification of mental workload levels using eeg and an adaptive deep learning model. *Biomedical Signal Processing and Control* **33** (2017) 30–47.

Zhang J, Yin Z, Wang R. Recognition of mental workload levels under complex human–machine collaboration by using physiological features and adaptive support vector machines. *IEEE Transactions on Human-Machine Systems* **45** (2015b) 200–214.

Zhou SM, Gan JQ, Sepulveda F. Classifying mental tasks based on features of higher-order statistics from eeg signals in brain–computer interface. *Information Sciences* **178** (2008) 1629–1640.

Yin Z, Zhang J. Recognition of mental workload levels by combining adaptive exponential feature smoothing and locality preservation projection techniques. *Control Conference (CCC), 2014 33rd Chinese* (IEEE) (2014), 4700–4705.

Liu Y, Subramaniam SCH, Sourina O, Liew SHP, Krishnan G, Konovessis D, et al. Eeg-based mental workload and stress recognition of crew members in maritime virtual simulator: a case study. *Cyberworlds (CW), 2017 International Conference on* (IEEE) (2017), 64–71.

Zhang J, Yin Z, Wang R. Pattern classification of instantaneous cognitive task-load through gmm clustering, laplacian eigenmap, and ensemble svms. *IEEE/ACM transactions on computational biology and bioinformatics* **14** (2017a) 947–965.

Plechawska-Wójcik M, Borys M. An analysis of eeg signal combined with pupillary response in the dynamics of human cognitive processing. *2016 9th International Conference on Human System Interactions (HSI)* (IEEE) (2016), 378–385.

Chen D, Vertegaal R. Using mental load for managing interruptions in physiologically attentive user interfaces. *CHI'04 extended abstracts on Human factors in computing systems* (ACM) (2004), 1513–1516.

Hirshfield LM, Solovey ET, Girouard A, Kebinger J, Jacob RJ, Sassaroli A, et al. Brain measurement for usability testing and adaptive interfaces: an example of uncovering syntactic workload with functional near infrared spectroscopy. *Proceedings of the SIGCHI Conference on Human Factors in Computing Systems* (ACM) (2009), 2185–2194.

Montgomery LD, Montgomery RW, Guisado R. Rheoencephalographic and electroencephalographic measures of cognitive workload: analytical procedures. *Biological Psychology* **40** (1995) 143–159.

De Bruin EA, Beersma DG, Daan S. Sustained mental workload does not affect subsequent sleep intensity. *Journal of sleep research* **11** (2002) 113–121.

Sammer G, Blecker C, Gebhardt H, Bischoff M, Stark R, Morgen K, et al. Relationship between regional hemodynamic activity and simultaneously recorded eeg-theta associated with mental arithmetic-induced workload. *Human brain mapping* **28** (2007) 793–803.

Marshall SP. The index of cognitive activity: Measuring cognitive workload. *Human factors and power plants, 2002. proceedings of the 2002 IEEE 7th conference on* (IEEE) (2002), 7–7.

Mathan S, Smart A, Ververs T, Feuerstein M. Towards an index of cognitive efficacy eeg-based estimation of cognitive load among individuals experiencing cancer-related cognitive decline. *Engineering in Medicine and Biology Society (EMBC), 2010 Annual International Conference of the IEEE* (IEEE) (2010), 6595–6598.

Hwang T, Kim M, Hwangbo M, Oh E. Optimal set of eeg electrodes for real-time cognitive workload monitoring. *Consumer Electronics (ISCE 2014), The 18th IEEE International Symposium on* (IEEE) (2014a), 1–2.

Zarjam P, Epps J, Lovell NH. Beyond subjective self-rating: Eeg signal classification of cognitive workload. *IEEE Transactions on Autonomous Mental Development* **7** (2015) 301–310.

Rozado D, Dunser A. Combining eeg with pupillometry to improve cognitive workload detection. *Computer* **48** (2015) 18–25.

Blanco JA, Johnson MK, Jaquess KJ, Oh H, Lo LC, Gentili RJ, et al. Quantifying cognitive workload in simulated flight using passive, dry eeg measurements. *IEEE Transactions on Cognitive and Developmental Systems* **10** (2018) 373–383.

Chang HC, Hung IC, Chew SW, Chen NS. Yet another objective approach for measuring cognitive load using eeg-based workload. *Advanced Learning Technologies (ICALT), 2016 IEEE 16th International Conference on* (IEEE) (2016), 501–502.

Mallick R, Slayback D, Touryan J, Ries AJ, Lance BJ. The use of eye metrics to index cognitive workload in video games. *2016 IEEE Second Workshop on Eye Tracking and Visualization (ETVIS)* (IEEE) (2016), 60–64.

Magnusdottir EH, Johannsdottir KR, Bean C, Olafsson B, Gudnason J. Cognitive workload classification using cardiovascular measures and dynamic features. *Cognitive Infocommunications (CogInfoCom), 2017 8th IEEE International Conference on* (IEEE) (2017), 000351–000356.

Almogbel MA, Dang AH, Kameyama W. Eeg-signals based cognitive workload detection of vehicle driver using deep learning. *Advanced Communication Technology (ICACT), 2018 20th International Conference on* (IEEE) (2018), 256–259.

Hwang T, Kim M, Hwangbo M, Oh E. Comparative analysis of cognitive tasks for modeling mental workload with electroencephalogram. *2014 36th Annual International Conference of the IEEE Engineering in Medicine and Biology Society* (IEEE) (2014b), 2661–2665.

Putze F, Schultz T, Prøpper R. Dummy model based workload modeling. *2015 IEEE International Conference on Systems, Man, and Cybernetics* (IEEE) (2015), 935–940.

Ke Y, Qi H, He F, Liu S, Zhao X, Zhou P, et al. An eeg-based mental workload estimator trained on working memory task can work well under simulated multi-attribute task. *Frontiers in human neuroscience* **8** (2014) 703.

Ke Y, Qi H, Zhang L, Chen S, Jiao X, Zhou P, et al. Towards an effective cross-task mental workload recognition model using electroencephalography based on feature selection and support vector machine regression. *International Journal of Psychophysiology* **98** (2015) 157–166.

Lim WL, Sourina O, Wang L, Liu Y. Individual alpha peak frequency based features for subject dependent eeg workload classification. *2016 IEEE International Conference on Systems, Man, and Cybernetics (SMC)* (IEEE) (2016), 003329–003333.

Kraft AE, Russo J, Krein M, Russell B, Casebeer W, Ziegler M. A systematic approach to developing near real-time performance predictions based on physiological measures. *2017 IEEE Conference on Cognitive and Computational Aspects of Situation Management (CogSIMA)* (IEEE) (2017), 1–7.

Oyama K, Takeuchi A, Chang CK. Brain lattice: concept lattice based causal analysis of changes in mental workload. *2013 IEEE International Multi-Disciplinary Conference on Cognitive Methods in Situation Awareness and Decision Support (CogSIMA)* (IEEE) (2013), 59–66.

Kothe CA, Makeig S. Estimation of task workload from eeg data: new and current tools and perspectives. *Engineering in Medicine and Biology Society, EMBC, 2011 Annual International Conference of the IEEE* (IEEE) (2011), 6547–6551.

Kramer AF, Sirevaag EJ, Braune R. A psychophysiological assessment of operator workload during simulated flight missions. *Human factors* **29** (1987) 145–160.

Laine TI, Bauer K, Lanning JW, Russell CA, Wilson GF. Selection of input features across subjects for classifying crewmember workload using artificial neural networks. *IEEE Transactions on Systems, Man, and Cybernetics-Part A: Systems and Humans* **32** (2002) 691–704.

Zhang J, Yin Z, Wang R. Nonlinear dynamic classification of momentary mental workload using physiological features and narx-model-based least-squares support vector machines. *IEEE Transactions on Human-Machine Systems* **47** (2017b) 536–549.

Herff C, Fortmann O, Tse CY, Cheng X, Putze F, Heger D, et al. Hybrid fnirs-eeg based discrimination of 5 levels of memory load. *2015 7th International IEEE/EMBS Conference on Neural Engineering (NER)* (IEEE) (2015), 5–8.

Bodala IP, Kukreja S, Li J, Thakor NV, Al-Nashash H. Eye tracking and eeg synchronization to analyze microsaccades during a workload task. *2015 37th Annual International Conference of the IEEE Engineering in Medicine and Biology Society (EMBC)* (IEEE) (2015), 7994–7997.

Klosterman SL, Estepp JR, Monnin JW, Christensen JC. Day-to-day variability in hybrid, passive brain-computer interfaces: Comparing two studies assessing cognitive workload. *2016 38th Annual International Conference of the IEEE Engineering in Medicine and Biology Society (EMBC)* (IEEE) (2016), 1584–1590.

Dimitrakopoulos GN, Kakkos I, Dai Z, Lim J, Bezerianos A, Sun Y, et al. Task-independent mental workload classification based upon common multiband eeg cortical connectivity. *IEEE Transactions on Neural Systems and Rehabilitation Engineering* **25** (2017) 1940–1949.

Wang Z, Hope RM, Wang Z, Ji Q, Gray WD. An eeg workload classifier for multiple subjects. *2011 Annual International Conference of the IEEE Engineering in Medicine and Biology Society* (IEEE) (2011), 6534–6537.

Hernández-Sabaté A, Yauri J, Folch P, Piera MÀ, Gil D. Recognition of the mental workloads of pilots in the cockpit using eeg signals. *Applied Sciences* **12** (2022) 2298.

Kutafina E, Heiligers A, Popovic R, Brenner A, Hankammer B, Jonas SM, et al. Tracking of mental workload with a mobile eeg sensor. *Sensors* **21** (2021) 5205.

Pei Z, Wang H, Bezerianos A, Li J. Eeg-based multiclass workload identification using feature fusion and selection. *IEEE Transactions on Instrumentation and Measurement* **70** (2020) 1–8.

Diaz-Piedra C, Sebastián MV, Di Stasi LL. Eeg theta power activity reflects workload among army combat drivers: an experimental study. *Brain sciences* **10** (2020) 199.

Kakkos I, Dimitrakopoulos GN, Sun Y, Yuan J, Matsopoulos GK, Bezerianos A, et al. Eeg fingerprints of task-independent mental workload discrimination. *IEEE Journal of Biomedical and Health Informatics* **25** (2021) 3824–3833.

Guan K, Zhang Z, Chai X, Tian Z, Liu T, Niu H. Eeg based dynamic functional connectivity analysis in mental workload tasks with different types of information. *IEEE Transactions on Neural Systems and Rehabilitation Engineering* **30** (2022) 632–642.

Raufi B, Longo L. An evaluation of the eeg alpha-to-theta and theta-to-alpha band ratios as indexes of mental workload. *Neuroinformatics* (2022). doi:10.3389/fninf.2022.861967.

Elkins JD, Hossain G. Multinomial processing models in visual cognitive effort diagnostics. *Proceedings of the IEEE Conference on Computer Vision and Pattern Recognition Workshops* (IEEE) (2015), 9–15.

Cegarra J, Chevalier A. Theoretical and methodological considerations in the comparison of performance and physiological measures of mental workload. *International Conference on Engineering Psychology and Cognitive Ergonomics* (Springer) (2007), 264–268.

de Greef T, Lafeber H, van Oostendorp H, Lindenberg J. Eye movement as indicators of mental workload to trigger adaptive automation. *International Conference on Foundations of Augmented Cognition* (Springer) (2009), 219–228.

He X, Wang L, Gao X, Chen Y. The eye activity measurement of mental workload based on basic flight task. *Industrial Informatics (INDIN), 2012 10th IEEE International Conference on* (IEEE) (2012), 502–507.

Iqbal ST, Zheng XS, Bailey BP. Task-evoked pupillary response to mental workload in human-computer interaction. *CHI'04 extended abstracts on Human factors in computing systems* (ACM) (2004), 1477–1480.

Schultheis H, Jameson A. Assessing cognitive load in adaptive hypermedia systems: Physiological and behavioral methods. *International Conference on Adaptive Hypermedia and Adaptive Web-Based Systems* (Springer) (2004), 225–234.

Zhang Y, Owechko Y, Zhang J. Driver cognitive workload estimation: A data-driven perspective. *Intelligent Transportation Systems* (Citeseer) (2004), 642–647.

Bailey BP, Iqbal ST. Understanding changes in mental workload during execution of goal-directed tasks and its application for interruption management. *ACM Transactions on Computer-Human Interaction (TOCHI)* **14** (2008) 21.

Iqbal ST, Adamczyk PD, Zheng XS, Bailey BP. Towards an index of opportunity: understanding changes in mental workload during task execution. *Proceedings of the SIGCHI conference on Human factors in computing systems* (ACM) (2005), 311–320.

Chen S, Epps J, Ruiz N, Chen F. Eye activity as a measure of human mental effort in hci. *Proceedings of the 16th international conference on Intelligent user interfaces* (ACM) (2011b), 315–318.

Xu J, Wang Y, Chen F, Choi H, Li G, Chen S, et al. Pupillary response based cognitive workload index under luminance and emotional changes. *CHI'11 Extended Abstracts on Human Factors in Computing Systems* (ACM) (2011), 1627–1632.

Wang L, He X, Chen Y. Distinguishing analysis on workload peak and overload under time pressure with pupil diameter. *2014 IEEE International Inter-Disciplinary Conference on Cognitive Methods in Situation Awareness and Decision Support (CogSIMA)* (IEEE) (2014), 151–155.

[Dataset] Bedziouk S, Kostin A, Golikov Y. System and method for mental workload measurement based on rapid eye movement (2006).

Davis I. Evoked potential, cardiac, blink, and respiration measures of pilot workload in air-to-ground missions. *Aviation, space, and environmental medicine* (1994).

Lin Y, Leng H, Yang G, Cai H. An intelligent noninvasive sensor for driver pulse wave measurement. *IEEE Sensors Journal* **7** (2007) 790–799.

Mehler B, Reimer B, Coughlin JF, Dusek JA. Impact of incremental increases in cognitive workload on physiological arousal and performance in young adult drivers. *Transportation Research Record* **2138** (2009) 6–12.

Ohsuga M, Shimono F, Genno H. Assessment of phasic work stress using autonomic indices. *International Journal of Psychophysiology* **40** (2001) 211–220.

Cinaz B, La Marca R, Arnrich B, Trøster G. Towards continuous monitoring of mental workload. *ACM UbiComp* (ACM) (2010).

Mahmoud R, Shanableh T, Bodala IP, Thakor NV, Al-Nashash H. Novel classification system for classifying cognitive workload levels under vague visual stimulation. *IEEE Sensors Journal* **17** (2017) 7019–7028.

Kumar M, Weippert M, Vilbrandt R, Kreuzfeld S, Stoll R. Fuzzy evaluation of heart rate signals for mental stress assessment. *IEEE Transactions on fuzzy systems* **15** (2007) 791–808.

Heine T, Lenis G, Reichensperger P, Beran T, Doessel O, Deml B. Electrocardiographic features for the measurement of drivers' mental workload. *Applied ergonomics* **61** (2017) 31–43.

Hjortskov N, Rissén D, Blangsted AK, Fallentin N, Lundberg U, Søgaard K. The effect of mental stress on heart rate variability and blood pressure during computer work. *European journal of applied physiology* **92** (2004) 84–89.

Itoh M. Individual differences in effects of secondary cognitive activity during driving on temperature at the nose tip. *2009 International Conference on Mechatronics and Automation* (IEEE) (2009), 7–11.

Boucsein W, Thum M. Design of work/rest schedules for computer work based on psychophysiological recovery measures. *International Journal of Industrial Ergonomics* **20** (1997) 51–57.

Schellekens JM, Sijtsma GJ, Vegter E, Meijman TF. Immediate and delayed after-effects of long lasting mentally demanding work. *Biological Psychology* **53** (2000) 37–56.

Cárdenas-Vélez D, Perales JC, Chirosa LJ, Conde-González J, Aguilar-Martínez D, Araya S. The effect of mental workload on the intensity and emotional dynamics of perceived exertion. *Anales de Psicología/Annals of Psychology* **29** (2013) 662–673.

Davis D, Oliver M, Byrne A. A novel method of measuring the mental workload of anaesthetists during simulated practice. *British journal of anaesthesia* **103** (2009) 665–669.

Murai K, Okazaki T, Hayashi Y. Measurement for mental workload of bridge team on leaving/entering port. *Position Location and Navigation Symposium, 2004. PLANS 2004* (IEEE) (2004), 746–751.

Twisk D, Boele M, Vlakveld W, Christoph M, Sikkema R, Remij R, et al. Preliminary results from a field experiment on e-bike safety: speed choice and mental workload for middle-aged and elderly cyclists. *Proceedings of the International Cycling Safety Conference 2013, ICSC2013, Helmond, The Netherlands, 20-21 November 2013* (ICSC) (2013).

Son J, Mehler B, Lee T, Park Y, Coughlin J, Reimer B. Impact of cognitive workload on physiological arousal and performance in younger and older drivers. *Proceedings of the Sixth International Driving*

Symposium on Human Factors in Driver Assessment, Training and Vehicle Design (University of Iowa) (2011), 87–94.

Ward RD, Marsden PH. Physiological responses to different web page designs. *International Journal of Human-Computer Studies* **59** (2003) 199–212.

Nickel P, Nachreiner F. Sensitivity and diagnosticity of the 0.1-hz component of heart rate variability as an indicator of mental workload. *Human factors* **45** (2003) 575–590.

Son J, Oh H, Park M. Identification of driver cognitive workload using support vector machines with driving performance, physiology and eye movement in a driving simulator. *International Journal of Precision Engineering and Manufacturing* **14** (2013) 1321–1327.

Mehler B, Reimer B, Wang Y. A comparison of heart rate and heart rate variability indices in distinguishing single-task driving and driving under secondary cognitive workload. *Proceedings of the Sixth International Driving Symposium on Human Factors in Driver Assessment, Training and Vehicle Design* (University of Iowa) (2011), 590–597.

Murai K, Hayashi Y. An evaluation of mental workload for effective navigation. *Interactive Technology and Smart Education* **5** (2008) 29–38.

Green P. Driver workload as a function of road geometry: A pilot experiment. Tech. rep., Michigan State University (1994).

Rowe DW, Sibert J, Irwin D. Heart rate variability: Indicator of user state as an aid to human-computer interaction. *Proceedings of the SIGCHI conference on Human factors in computing systems* (ACM Press/Addison-Wesley Publishing Co.) (1998), 480–487.

Hankins TC, Wilson GF. A comparison of heart rate, eye activity, eeg and subjective measures of pilot mental workload during flight. *Aviation, space, and environmental medicine* **69** (1998) 360–367.

Hama K, Murai K, Hayashi Y, Stone LC. Evaluation of ship navigator's mental workload for ship handling based on physiological indices. *Systems, Man and Cybernetics, 2009. SMC 2009. IEEE International Conference on* (IEEE) (2009), 228–232.

Di Nocera F, Terenzi M, Camilli M. Another look at scanpath: distance to nearest neighbour as a measure of mental workload. *Developments in human factors in transportation, design, and evaluation* (2006) 295–303.

Van Roon AM, Mulder LJ, Althaus M, Mulder G. Introducing a baroreflex model for studying cardiovascular effects of mental workload. *Psychophysiology* **41** (2004) 961–981.

Nourbakhsh N, Wang Y, Chen F, Calvo RA. Using galvanic skin response for cognitive load measurement in arithmetic and reading tasks. *Proceedings of the 24th Australian Computer-Human Interaction Conference* (ACM) (2012), 420–423.

Tanaka J, Ishida S, Kawagoe H, Kondo S. Workload of using a driver assistance system. *Intelligent Transportation Systems, 2000. Proceedings. 2000 IEEE* (IEEE) (2000), 382–386.

Zhang W, White M, Zahabi M, Winslow AT, Zhang F, Huang H, et al. Cognitive workload in conventional direct control vs. pattern recognition control of an upper-limb prosthesis. *2016 IEEE International Conference on Systems, Man, and Cybernetics (SMC)* (IEEE) (2016), 002335–002340.

Rani P, Liu C, Sarkar N, Vanman E. An empirical study of machine learning techniques for affect recognition in human–robot interaction. *Pattern Analysis and Applications* **9** (2006) 58–69.

Unni A, Ihme K, Surm H, Weber L, Lüdtke A, Nicklas D, et al. Brain activity measured with fnirs for the prediction of cognitive workload. *2015 6th IEEE International Conference on Cognitive Infocommunications (CogInfoCom)* (IEEE) (2015), 349–354.

Ung W, Tang T, Meriaudeau F, Ebenezer E. Dynamic optimization of mental workload in fnirs-bci system for cognitive rehabilitation. *2017 IEEE International Conference on Signal and Image Processing Applications (ICSIPA)* (IEEE) (2017), 320–323.

Berivanlou NH, Setarehdan SK, Noubari HA. Quantifying mental workload of operators performing n-back working memory task: Toward fnirs based passive bci system. *2016 23rd Iranian Conference on Biomedical Engineering and 2016 1st International Iranian Conference on Biomedical Engineering (ICBME)* (IEEE) (2016), 140–145.

Durantin G, Gagnon JF, Tremblay S, Dehais F. Using near infrared spectroscopy and heart rate variability to detect mental overload. *Behavioural brain research* **259** (2014) 16–23.

Sibi S, Ayaz H, Kuhns DP, Sirkin DM, Ju W. Monitoring driver cognitive load using functional near infrared spectroscopy in partially autonomous cars. *2016 IEEE Intelligent Vehicles Symposium (IV)* (IEEE) (2016), 419–425.

Karim H, Schmidt B, Dart D, Beluk N, Huppert T. Functional near-infrared spectroscopy (fnirs) of brain function during active balancing using a video game system. *Gait & posture* **35** (2012) 367–372.

Sassaroli A, Zheng F, Hirshfield LM, Girouard A, Solovey ET, Jacob RJ, et al. Discrimination of mental workload levels in human subjects with functional near-infrared spectroscopy. *Journal of Innovative Optical Health Sciences* **1** (2008) 227–237.

Li Lp, Liu Zg, Zhu Hy, Zhu L, Huang Yc. Functional near-infrared spectroscopy in the evaluation of urban rail transit drivers' mental workload under simulated driving conditions. *Ergonomics* **62** (2019) 406–419.

Parshi S, Amin R, Azgomi HF, Faghih RT. Mental workload classification via hierarchical latent dictionary learning: A functional near infrared spectroscopy study. *2019 IEEE EMBS International Conference on Biomedical & Health Informatics (BHI)* (IEEE) (2019), 1–4.

Galoyan T, Betts K, Abramian H, Reddy P, Izzetoglu K, Shewokis PA. Examining mental workload in a spatial navigation transfer game via functional near infrared spectroscopy. *Brain Sciences* **11** (2021) 45.

Yeung AS, Lee CFK, Pena IM, Ryde J. Toward a subjective mental workload measure. *International Congress for School Effectiveness and Improvement* (ERIC) (2000).

Bertram DA, Hershey CO, Opila DA, Quirin O. A measure of physician mental work load in internal medicine ambulatory care clinics. *Medical care* (1990) 458–467.

Bertram DA, Opila DA, Brown JL, Gallagher SJ, Schifeling RW, Snow IS, et al. Measuring physician mental workload: reliability and validity assessment of a brief instrument. *Medical Care* (1992) 95–104.

Lin DY, Hwang SL. The development of mental workload measurement in flexible manufacturing systems. *Human Factors and Ergonomics in Manufacturing & Service Industries* **8** (1998) 41–62.

Martens M, Van Winsum W. Measuring distraction: the peripheral detection task. *TNO Human Factors, Soesterberg, Netherlands* (2000).

Wastlund E, Reinikka H, Norlander T, Archer T. Effects of vdt and paper presentation on consumption and production of information: Psychological and physiological factors. *Computers in human behavior* **21** (2005) 377–394.

Kum S, Furusho M, Iwasaki H. Investigation on the factors of vts operators' mental workload: case of turkish operators. *International Maritime Lecturers Association 16th Conference, Izmir, October* (2008b), 14–17.

Weigl M, Muller A, Angerer P, Hoffmann F. Workflow interruptions and mental workload in hospital pediatricians: an observational study. *BMC health services research* **14** (2014) 433.

Azadeh A, Rouzbahman M, Saberi M, Valianpour F, Keramati A. Improved prediction of mental workload versus hse and ergonomics factors by an adaptive intelligent algorithm. *Safety science* **58** (2013) 59–75.

Mangen A, Walgermo BR, Brønnick K. Reading linear texts on paper versus computer screen: Effects on reading comprehension. *International journal of educational research* **58** (2013) 61–68.

Pretorius A, Cilliers P. Development of a mental workload index: A systems approach. *Ergonomics* **50** (2007) 1503–1515.

Zhao X, Hsu CY, Chang PC, Li L. A genetic algorithm for the multi-objective optimization of mixed-model assembly line based on the mental workload. *Engineering Applications of Artificial Intelligence* **47** (2016) 140–146.

Shingledecker CA. Behavioral and subjective workload metrics for operational environments. Tech. rep., Air Force Aerospace Medical Research Lab Wright-Patterson AFB OH (1983).

Lysaght RJ, Hill SG, Dick A, Plamondon BD, Linton PM. Operator workload: Comprehensive review and evaluation of operator workload methodologies. Tech. rep., Analytics Inc Willow Grove PA (1989).

Parasuraman R, Sheridan TB, Wickens CD. A model for types and levels of human interaction with automation. *IEEE Transactions on systems, man, and cybernetics-Part A: Systems and Humans* **30** (2000) 286–297.

Hart SG. Nasa-task load index (nasa-tlx); 20 years later. *Proceedings of the human factors and ergonomics society annual meeting* **50** (2006) 904–908.

Gørges M, Staggers N. Evaluations of physiological monitoring displays: a systematic review. *Journal of clinical monitoring and computing* **22** (2008) 45.

Hoc JM. Towards a cognitive approach to human–machine cooperation in dynamic situations. *International journal of human-computer studies* **54** (2001) 509–540.

Dekker S, Hollnagel E. Human factors and folk models. *Cognition, Technology & Work* **6** (2004) 79–86.

Zhang Y, Luximon A. Subjective mental workload measures. *Ergonomia* **3** (2005).

Segall N, Doolen TL, Porter JD. A usability comparison of pda-based quizzes and paper-and-pencil quizzes. *Computers & Education* **45** (2005) 417–432.

Wilson MR, Poolton JM, Malhotra N, Ngo K, Bright E, Masters RS. Development and validation of a surgical workload measure: the surgery task load index (surg-tlx). *World journal of surgery* **35** (2011) 1961.

de Winter JC. Controversy in human factors constructs and the explosive use of the nasa-tlx: a measurement perspective. *Cognition, technology & work* **16** (2014) 289–297.

Nakagawa T, Kamei Y, Uwano H, Monden A, Matsumoto K, German DM. Quantifying programmers' mental workload during program comprehension based on cerebral blood flow measurement: a controlled experiment. *Companion Proceedings of the 36th International Conference on Software Engineering* (ACM) (2014), 448–451.

Verwey WB, Veltman HA. Detecting short periods of elevated workload: A comparison of nine workload assessment techniques. *Journal of experimental psychology: Applied* **2** (1996) 270.

Hart SG, Staveland LE. Development of nasa-tlx (task load index): Results of empirical and theoretical research. *Advances in psychology* (Elsevier), vol. 52 (1988), 139–183.

Fuller R. Towards a general theory of driver behaviour. *Accident Analysis & Prevention* **37** (2005) 461–472.

da Silva FP. Mental workload, task demand and driving performance: What relation. *Procedia-Social and Behavioral Sciences* **162** (2014) 310–319.

De Winter JC, Happee R, Martens MH, Stanton NA. Effects of adaptive cruise control and highly automated driving on workload and situation awareness: A review of the empirical evidence. *Transportation research part F: traffic psychology and behaviour* **27** (2014) 196–217.

Hancock PA, Meshkati N, Robertson M. Physiological reflections of mental workload. *Aviation, space, and environmental medicine* (1985).

May JG, Kennedy RS, Williams MC, Dunlap WP, Brannan JR. Eye movement indices of mental workload. *Acta psychologica* **75** (1990) 75–89.

Tokuda S, Obinata G. Development of an algorithm to detect saccadic intrusions as an index of mental workload. *SICE Annual Conference (SICE), 2012 Proceedings of* (IEEE) (2012), 1369–1372.

Karatas N, Yoshikawa S, De Silva PR, Okada M. Namida: How to reduce the cognitive workload of driver. *The Eleventh ACM/IEEE International Conference on Human Robot Interaction* (IEEE Press) (2016), 449–450.

Kakkos I, Dimitrakopoulos GN, Gao L, Zhang Y, Qi P, Matsopoulos GK, et al. Mental workload drives different reorganizations of functional cortical connectivity between 2d and 3d simulated flight experiments. *IEEE Transactions on Neural Systems and Rehabilitation Engineering* **27** (2019) 1704–1713.

Foy HJ, Chapman P. Mental workload is reflected in driver behaviour, physiology, eye movements and prefrontal cortex activation. *Applied ergonomics* **73** (2018) 90–99.

Fan X, Zhao C, Zhang X, Luo H, Zhang W. Assessment of mental workload based on multi-physiological signals. *Technology and Health Care* **28** (2020) 67–80.

Delliaux S, Delaforge A, Deharo JC, Chaumet G. Mental workload alters heart rate variability, lowering non-linear dynamics. *Frontiers in physiology* **10** (2019) 565.

Chakladar DD, Dey S, Roy PP, Dogra DP. Eeg-based mental workload estimation using deep blstm-lstm network and evolutionary algorithm. *Biomedical Signal Processing and Control* **60** (2020) 101989.

Rosanne O, Albuquerque I, Cassani R, Gagnon JF, Tremblay S, Falk TH. Adaptive filtering for improved eeg-based mental workload assessment of ambulant users. *Frontiers in Neuroscience* **15** (2021) 341.

Yang Y, Chen Y, Wu C, Easa SM, Lin W, Zheng X. Effect of highway directional signs on driver mental workload and behavior using eye movement and brain wave. *Accident Analysis & Prevention* **146** (2020) 105705.

Islam MR, Barua S, Ahmed MU, Begum S, Aricò P, Borghini G, et al. A novel mutual information based feature set for drivers' mental workload evaluation using machine learning. *Brain Sciences* **10** (2020) 551.

Ho TKK, Gwak J, Park CM, Song JI. Discrimination of mental workload levels from multi-channel fnirs using deep leaning-based approaches. *IEEE Access* **7** (2019) 24392–24403.

Morales JM, Ruiz-Rabelo JF, Diaz-Piedra C, Di Stasi LL. Detecting mental workload in surgical teams using a wearable single-channel electroencephalographic device. *Journal of surgical education* **76** (2019) 1107–1115.

Van Acker BB, Bombeke K, Durnez W, Parmentier DD, Mateus JC, Biondi A, et al. Mobile pupillometry in manual assembly: A pilot study exploring the wearability and external validity of a renowned mental workload lab measure. *International Journal of Industrial Ergonomics* **75** (2020) 102891.

Putze F, Herff C, Tremmel C, Schultz T, Krusienski DJ. Decoding mental workload in virtual environments: a fnirs study using an immersive n-back task. *2019 41st Annual International Conference of the IEEE Engineering in Medicine and Biology Society (EMBC)* (IEEE) (2019), 3103–3106.

Di Flumeri G, Borghini G, Aricò P, Sciaraffa N, Lanzi P, Pozzi S, et al. Eeg-based mental workload assessment during real driving: A taxonomic tool for neuroergonomics in highly automated environments. *Neuroergonomics* (Elsevier) (2019), 121–126.

Ayaz H, Shewokis PA, Bunce S, Izzetoglu K, Willems B, Onaral B. Optical brain monitoring for operator training and mental workload assessment. *Neuroimage* **59** (2012) 36–47.

Cha DW, Park P. Comparative study of subjective mental workload assessment techniques for the evaluation of its-oriented human-machine interface systems. *Journal of Korean Society of Transportation* **19** (2001) 45–58.

Czaja SJ, Sharit J. Age differences in attitudes toward computers. *The Journals of Gerontology Series B: Psychological Sciences and Social Sciences* **53** (1998) P329–P340.

Chen SM. New methods for subjective mental workload assessment and fuzzy risk analysis. *Cybernetics & Systems* **27** (1996) 449–472.

Marcora SM, Staiano W, Manning V. Mental fatigue impairs physical performance in humans. *Journal of applied physiology* **106** (2009) 857–864.

Bellotti F, De Gloria A, Montanari R, Dosio N, Morreale D. Comunicar: designing a multimedia, context-aware human-machine interface for cars. *Cognition, Technology & Work* **7** (2005) 36–45.

Kuijer PPF, van der Beek AJ, van Dieën JH, Visser B, Frings-Dresen MH. Effect of job rotation on need for recovery, musculoskeletal complaints, and sick leave due to musculoskeletal complaints: a prospective study among refuse collectors. *American journal of industrial medicine* **47** (2005) 394–402.

Tarola CL, Hirji S, Yule SJ, Gabany JM, Zenati A, Dias RD, et al. Cognitive support to promote shared mental models during safety-critical situations in cardiac surgery (late breaking report). *2018 IEEE Conference on Cognitive and Computational Aspects of Situation Management (CogSIMA)* (IEEE) (2018), 165–167.

Chin E, Nathan F, Pauzie A, Manzano J, Nodari E, Cherri C, et al. Subjective assessment methods for workload. *AIDE Deliverable* **2** (2004).

Vidulich MA, Wickens CD. Causes of dissociation between subjective workload measures and performance: Caveats for the use of subjective assessments. *Applied Ergonomics* **17** (1986) 291–296.

Shan Y, Shang J, Yan Y, Lu G, Hu D, Ye X. Mental workload of frontline nurses aiding in the covid-19 pandemic: A latent profile analysis. *Journal of Advanced Nursing* **77** (2021) 2374–2385.

Pourteimour S, Yaghmaei S, Babamohamadi H. The relationship between mental workload and job performance among iranian nurses providing care to covid-19 patients: A cross-sectional study. *Journal of Nursing Management* **29** (2021) 1723–1732.

Zhang J, Pang L, Cao X, Wanyan X, Wang X, Liang J, et al. The effects of elevated carbon dioxide concentration and mental workload on task performance in an enclosed environmental chamber. *Building and Environment* **178** (2020) 106938.

Mansikka H, Virtanen K, Harris D. Comparison of nasa-tlx scale, modified cooper–harper scale and mean inter-beat interval as measures of pilot mental workload during simulated flight tasks. *Ergonomics* **62** (2019) 246–254.

Qiao H, Zhang J, Zhang L, Li Y, Loft S. Exploring the peak-end effects in air traffic controllers' mental workload ratings. *Human factors* (2021) 0018720821994355.

Jansen EC, Peterson KE, O'Brien L, Hershner S, Boolani A. Associations between mental workload and sleep quality in a sample of young adults recruited from a us college town. *Behavioral sleep medicine* **18** (2020) 513–522.

Abe T, Dar F, Amnattrakul P, Aydin A, Raison N, Shinohara N, et al. The effect of repeated full immersion simulation training in ureterorenoscopy on mental workload of novice operators. *BMC medical education* **19** (2019) 1–9.

Galy E, Paxion J, Berthelon C. Measuring mental workload with the nasa-tlx needs to examine each dimension rather than relying on the global score: an example with driving. *Ergonomics* **61** (2018) 517–527.

Takada Y, Shimoyama O. Evaluation of driving-assistance systems based on drivers' workload. *First International Driving Symposium on Human Factors in Driver Assessment, Training and Vehicle Design* (University of Iowa) (2001), 208–213.

Jimenez-Molina A, Retamal C, Lira H. Using psychophysiological sensors to assess mental workload during web browsing. *Sensors* **18** (2018) 458.

Wang X, Li D, Menassa CC, Kamat VR. Investigating the effect of indoor thermal environment on occupants' mental workload and task performance using electroencephalogram. *Building and Environment* **158** (2019) 120–132.

Spinelli R, Magagnotti N, Labelle ER. The effect of new silvicultural trends on mental workload of harvester operators. *Croatian Journal of Forest Engineering: Journal for Theory and Application of Forestry Engineering* **41** (2020) 1–13.

Midha S, Maior HA, Wilson ML, Sharples S. Measuring mental workload variations in office work tasks using fnirs. *International Journal of Human-Computer Studies* **147** (2021) 102580.

Luong T, Martin N, Raison A, Argelaguet F, Diverrez JM, Lécuyer A. Towards real-time recognition of users mental workload using integrated physiological sensors into a vr hmd. *2020 IEEE International Symposium on Mixed and Augmented Reality (ISMAR)* (IEEE) (2020), 425–437.

Bao J, Song X, Li Y, Bai Y, Zhou Q. Effect of lighting illuminance and colour temperature on mental workload in an office setting. *Scientific reports* **11** (2021) 1–10.

Das S, Maiti J, Krishna O. Assessing mental workload in virtual reality based eot crane operations: A multi-measure approach. *International Journal of Industrial Ergonomics* **80** (2020) 103017.

Yan S, Wei Y, Tran CC. Evaluation and prediction mental workload in user interface of maritime operations using eye response. *International Journal of Industrial Ergonomics* **71** (2019) 117–127.

Rad"untz T, F"urstenau N, M"uhlhausen T, Meffert B. Indexing mental workload during simulated air traffic control tasks by means of dual frequency head maps. *Frontiers in physiology* **11** (2020) 300.

Rainieri G, Fraboni F, Russo G, Tul M, Pingitore A, Tessari A, et al. Visual scanning techniques and mental workload of helicopter pilots during simulated flight. *Aerospace medicine and human performance* **92** (2021) 11–19.

Geissler CF, Schneider J, Frings C. Shedding light on the prefrontal correlates of mental workload in simulated driving: a functional near-infrared spectroscopy study. *Scientific reports* **11** (2021) 1–10.

Marinescu AC, Sharples S, Ritchie AC, Sanchez Lopez T, McDowell M, Morvan HP. Physiological parameter response to variation of mental workload. *Human factors* **60** (2018) 31–56.

Berguer R, Smith W. An ergonomic comparison of robotic and laparoscopic technique: the influence of surgeon experience and task complexity. *Journal of Surgical Research* **134** (2006) 87–92.

Faure V, Lobjois R, Benguigui N. The effects of driving environment complexity and dual tasking on drivers' mental workload and eye blink behavior. *Transportation research part F: traffic psychology and behaviour* **40** (2016) 78–90.

Tang Y, Zeng Y, et al. Quantifying designer's mental stress in the conceptual design process using kinesics study. *DS 58-9: Proceedings of ICED 09, the 17th International Conference on Engineering Design, Vol. 9, Human Behavior in Design, Palo Alto, CA, USA, 24.-27.08. 2009* (2009), 211–220.

Iani C, Gopher D, Lavie P. Effects of task difficulty and invested mental effort on peripheral vasoconstriction. *Psychophysiology* **41** (2004) 789–798.

Koo TY, Park KJ, Kim BY, Kim HJ, Suh MW. A study on driver's workload of telematics using a driving simulator: A comparison among information modalities. *International journal of precision engineering and manufacturing* **10** (2009) 59.

Meier M, Borsky M, Magnusdottir EH, Johannsdottir KR, Gudnason J. Vocal tract and voice source features for monitoring cognitive workload. *2016 7th IEEE International Conference on Cognitive Infocommunications (CogInfoCom)* (IEEE) (2016), 000097–000102.

Liao Y, Li G, Li SE, Cheng B, Green P. Understanding driver response patterns to mental workload increase in typical driving scenarios. *IEEE Access* **6** (2018) 35890–35900.

Dunlop MD, Davidson N. Visual information seeking on palmtop devices. *People and Computers XIV - Usability or Else, HCI 2000* (Springer) (2000).

MacMillan J, Entin EE, Serfaty D. Communication overhead: The hidden cost of team cognition. *Team cognition: Process and performance at the interand intra-individual level. American Psychological Association, Washington, DC. Available at http://www. aptima. com/publications/2004_MacMillan_EntinEE_Serfaty. pdf* (2004).

Moray N, Eisen P, Money L, Turksen I. Fuzzy analysis of skill and rule-based mental workload. *Advances in Psychology* (Elsevier), vol. 52 (1988), 289–304.

Patten CJ, Kircher A, Østlund J, Nilsson L. Using mobile telephones: cognitive workload and attention resource allocation. *Accident analysis & prevention* **36** (2004) 341–350.

Warm JS, Dember WN, Parasuraman R. Effects of olfactory stimulation on performance and stress. *Journal of the Society Cosmetic Chemists* **42** (1991) 199–210.

Helton WS, Warm JS. Signal salience and the mindlessness theory of vigilance. *Acta psychologica* **129** (2008) 18–25.
Horrey WJ, Lesch MF, Garabet A. Dissociation between driving performance and drivers' subjective estimates of performance and workload in dual-task conditions. *Journal of safety research* **40** (2009) 7–12.
Sandrock S, Schutte M, Griefahn B. Impairing effects of noise in high and low noise sensitive persons working on different mental tasks. *International archives of occupational and environmental health* **82** (2009) 779.
Bertolo D, Dinet J, Vivian R. Reducing cognitive workload during 3d geometry problem solving with an app on ipad. *2014 Science and Information Conference* (IEEE) (2014), 896–900.
Çapalar J, Olaverri-Monreal C. Hypovigilance in limited self-driving automation: Peripheral visual stimulus for a balanced level of automation and cognitive workload. *2017 IEEE 20th International Conference on Intelligent Transportation Systems (ITSC)* (IEEE) (2017), 27–31.
Meza-Kubo V, Gonzalez-Fraga A, Morán AL, Tentori M. Augmenting cognitive stimulation activities in a nursing home through pervasive computing. *2009 Latin American Web Congress* (IEEE) (2009), 8–15.
Li X, Vaezipour A, Rakotonirainy A, Demmel S, Oviedo-Trespalacios O. Exploring drivers' mental workload and visual demand while using an in-vehicle hmi for eco-safe driving. *Accident Analysis & Prevention* **146** (2020) 105756.
van Gent P, Melman T, Farah H, Van Nes N, van Arem B. Multi-level driver workload prediction using machine learning and off-the-shelf sensors. *Transportation Research Record* (2018) 0361198118790372.
Ding Y, Cao Y, Duffy VG, Wang Y, Zhang X. Measurement and identification of mental workload during simulated computer tasks with multimodal methods and machine learning. *Ergonomics* **63** (2020) 896–908.
Solís-Marcos I, Kircher K. Event-related potentials as indices of mental workload while using an in-vehicle information system. *Cognition, Technology & Work* **21** (2019) 55–67.
Chen Y, Yan S, Tran CC. Comprehensive evaluation method for user interface design in nuclear power plant based on mental workload. *Nuclear Engineering and Technology* **51** (2019) 453–462.
Bommer SC, Fendley M. A theoretical framework for evaluating mental workload resources in human systems design for manufacturing operations. *International Journal of Industrial Ergonomics* **63** (2018) 7–17.
Orlandi L, Brooks B. Measuring mental workload and physiological reactions in marine pilots: Building bridges towards redlines of performance. *Applied ergonomics* **69** (2018) 74–92.
Hancock PA, Chignell MH. Mental workload dynamics in adaptive interface design. *IEEE transactions on Systems, Man, and Cybernetics* **18** (1988) 647–658.
North RA, Riley VA. W/index: A predictive model of operator workload. *Applications of human performance models to system design* (Springer) (1989), 81–89.
Reid GB, Nygren TE. The subjective workload assessment technique: A scaling procedure for measuring mental workload. *Advances in psychology* (Elsevier), vol. 52 (1988), 185–218.
Rencken W, Durrant-Whyte H. On-line estimation of human operator workload. *Decision and Control, 1989., Proceedings of the 28th IEEE Conference on* (IEEE) (1989), 785–786.
Rouse WB, Edwards SL, Hammer JM. Modeling the dynamics of mental workload and human performance in complex systems. *IEEE transactions on systems, man, and cybernetics* **23** (1993) 1662–1671.
Zhang P, Wang X, Zhang W, Chen J. Learning spatial–spectral–temporal eeg features with recurrent 3d convolutional neural networks for cross-task mental workload assessment. *IEEE Transactions on neural systems and rehabilitation engineering* **27** (2018) 31–42.
Her CC, Hwang SL. Application of queueing theory to quantify information workload in supervisory control systems. *International Journal of Industrial Ergonomics* **4** (1989) 51–60.
Wilson GF, Russell CA. Real-time assessment of mental workload using psychophysiological measures and artificial neural networks. *Human factors* **45** (2003b) 635–644.
